# TraitTrainR: accelerating large-scale simulation under models of continuous trait evolution

**DOI:** 10.1093/bioadv/vbae196

**Published:** 2024-12-09

**Authors:** Jenniffer Roa Lozano, Mataya Duncan, Duane D McKenna, Todd A Castoe, Michael DeGiorgio, Richard Adams

**Affiliations:** Center for Agricultural Data Analytics, University of Arkansas, Fayetteville, AR 72701, United States; Department of Entomology and Plant Pathology, University of Arkansas, Fayetteville, AR 72701, United States; Center for Agricultural Data Analytics, University of Arkansas, Fayetteville, AR 72701, United States; Department of Entomology and Plant Pathology, University of Arkansas, Fayetteville, AR 72701, United States; Department of Biological Sciences, University of Memphis, Memphis, TN 38152, United States; Center for Biodiversity Research, University of Memphis, Memphis, TN 38152, United States; Department of Biology, University of Texas at Arlington, Arlington, TX 76010, United States; Department of Electrical Engineering and Computer Science, Florida Atlantic University, Boca Raton, FL 33431, United States; Center for Agricultural Data Analytics, University of Arkansas, Fayetteville, AR 72701, United States; Department of Entomology and Plant Pathology, University of Arkansas, Fayetteville, AR 72701, United States

## Abstract

**Motivation:**

The scale and scope of comparative trait data are expanding at unprecedented rates, and recent advances in evolutionary modeling and simulation sometimes struggle to match this pace. Well-organized and flexible applications for conducting large-scale simulations of evolution hold promise in this context for understanding models and more so our ability to confidently estimate them with real trait data sampled from nature.

**Results:**

We introduce *TraitTrainR*, an R package designed to facilitate efficient, large-scale simulations under complex models of continuous trait evolution. *TraitTrainR* employs several output formats, supports popular trait data transformations, accommodates multi-trait evolution, and exhibits flexibility in defining input parameter space and model stacking. Moreover, *TraitTrainR* permits measurement error, allowing for investigation of its potential impacts on evolutionary inference. We envision a wealth of applications of *TraitTrainR*, and we demonstrate one such example by examining the problem of evolutionary model selection in three empirical phylogenetic case studies. Collectively, these demonstrations of applying *TraitTrainR* to explore problems in model selection underscores its utility and broader promise for addressing key questions, including those related to experimental design and statistical power, in comparative biology.

**Availability and implementation:**

*TraitTrainR* is developed in R 4.4.0 and is freely available at https://github.com/radamsRHA/TraitTrainR/, which includes detailed documentation, quick-start guides, and a step-by-step tutorial.

## 1 Introduction

Modern comparative studies are flooded with biological trait data of varying scope, scale, and complexity. This deluge is due in part to advances in high-throughput phenotyping and sequencing for generating trait data across levels of biological organization—from single cells (Church *et al.* 2024) and tissues (Tirosh *et al.* 2007) to entire organisms (Ellegren 2014), populations (Hoban *et al.* 2012), and species (Hudson 2008). New large-scale trait databases are also rapidly coming online to curate a great deal of biodiversity (e.g. Tobias *et al.* 2022, Blackburn and Hughes 2024). The types of traits that can be measured and the questions that can be assessed with this information seem almost endless. However, as the complexity and breadth of comparative data continue to expand, so do the computational demands for analyzing them (De Los Campos *et al.* 2018, Adams *et al.* 2021, Pennell and Harmon 2013; Dimayacyac *et al.* 2023).

In the wake of these advances, the last few decades have seen a resurgence in the sophistication of probabilistic models for studying trait evolution. A number of software tools exist for simulating and fitting models of continuous trait evolution according to Brownian motion (BM) and related processes, including the popular packages ape, geiger, and phytools (Revell 2013, Pennell *et al.* 2014, Paradis and Schliep 2019). These approaches represent marked progress in simulation, inference, and mathematical modeling of evolution, which have been extended to incorporate additional considerations, features, and processes of evolution (e.g. Jhwueng and Maroulas 2016, Mazel *et al.* 2016, Ho and Dinh 2022, Jhwueng 2023, Vu *et al.* 2023), including extensions of BM, such as Ornstein-Uhlenbeck (OU; Hansen 1997, Beaulieu *et al.* 2012, Rohlfs *et al.* 2014, Blomberg *et al.* 2020), Early-Burst (EB; Harmon *et al.* 2010, Ingram *et al.* 2012), and Pagel's Lambda, Delta, and Kappa models (Gould and Eldredge 1977, Pagel 1999, Lepage *et al.* 2007, Ho and Dinh 2022). These models have been tailored to address a broad spectrum of biological questions (Bomprezzi *et al.* 2003, Sinoquet and Mourad 2014, Sukumaran and Knowles 2018), statistical challenges (Ho and Ané 2014), and data types (Butler and King 2004). Built upon the principles proposed in Felsenstein (1985), these models have emerged as a cornerstone of modern phylogenetic comparative methods (PCMs) central to comparative biology in the 21st century. While such advances hold great promise for improving evolutionary inference, a persistent question exists: how accurately do current models capture evolutionary processes in nature? Addressing this question requires a deeper understanding of current models and their alignment with empirical trait data.

Fortunately, a promising approach for learning about a model involves simulating many replicate datasets under that model (Arenas *et al.* 2012, Diniz-Filho *et al.* 2012, Hoban *et al.* 2012, Martin *et al.* 2023). Simulation-based strategies can help us better understand expected model outcomes, their predicted trait distributions, and other considerations for studying real trait data collected from nature (Kutsukake and Innan 2013, Boucher and Démery 2016). We can leverage large-scale simulations to understand theoretical and practical applications of model inference and the performance of statistical procedures in certain experimental and evolutionary conditions (e.g. Kutsukake and Innan 2013). Moreover, such strategies can be especially helpful when likelihood functions are expensive to compute or unavailable (Kutsukake and Innan 2013), and for methods that make use of simulations directly for inference, including machine learning techniques (Voznica *et al.* 2022), Bayesian approaches such as posterior prediction (Boettiger *et al.* 2012, Pennell *et al.* 2014) and approximate Bayesian computation (Bollback 2002, Gutmann *et al.* 2018), and maximum likelihood-based methods (Zhu *et al.* 2016).

Yet, the computational demands of conducting effective and well-organized simulations under complex evolutionary models can quickly become infeasible (e.g. Smith and Hahn 2023) or at least burdensome (e.g. Kutsukake and Innan 2013) as the scale of analysis increases, imposing a significant barrier. Moreover, it is often desirable (if not necessary) to incorporate variability in the evolutionary processes and parameters that affect trait distributions across replicates to accommodate uncertainty or limit conditions to an expected range, rather than fixing them to a constant value for all replicates (Freckleton 2009). For instance, many models of trait evolution are based on principles of BM (Felsenstein 1985, Hopkins and Lidgard 2012), which includes an ancestral state $z_{0}$ (i.e. trait value at the root node of a phylogeny) and evolutionary rate parameter $\sigma^{2}$. Conducting many replicate simulations with the same fixed values for $z_{0}\;$and $\sigma^{2}\;$may be neither helpful nor realistic. Instead, we may prefer sampling parameter values from a particular distribution to accommodate evolutionary variation across replicates. This can be accomplished, e.g. by sampling values of $\sigma^{2}$ from an exponential, uniform, or other applicable continuous distributions. Likewise, we can sample values of other relevant evolutionary parameters when conducting simulation under other models (e.g. sampling the α parameter of the OU model).

Clearly, probabilistic trait models thus provide valuable frameworks for understanding evolution. However, what is sometimes less clear or accessible is the expected trait distribution under some complex models (such as those incorporating non-Brownian processes), how large-scale simulations can be conducted efficiently with phylogenetic transformations, and perhaps how current approaches to model fit and inference behave in realistic conditions. What also remains uncertain is model inference performance for diverse phylogenetic backgrounds and in the presence of trait measurement error. Moreover, recent modeling efforts include complex evolutionary processes known to present statistical challenges, including an “ancestral shift model” (termed “AncShift” here), which prompted discussions about the need to reassess current models and assumptions (Uyeda et al. 2018), and yet, straightforward simulation frameworks under this model are lacking. This model incorporates instantaneous jumps in the mean trait value on ancestral branches of the phylogeny, which violates continuous trait evolution assumed by models based on BM (Uyeda et al. 2018; Adams *et al.* 2024). Additionally, local rates model (termed “lrates” here) refers to a model that allows for the evolution of traits at different rates across different branches of the phylogeny, which can further complicate model fitting and inference (Adams 2014, Castiglione *et al.* 2018). Because many canonical models of trait evolution are based on principles and extensions of BM, they can be reformulated as phylogenetic transformations, holding promise for incorporating more complex models and novel simulations that include multiple process levels, such as a “stacked” BM+AncShift model that integrates features of both processes. Regardless of whether model understanding, model inference, or both are the desired goals, the capability to conduct large-scale simulations under a set of target models is therefore imperative.

## 2 Methods

### 2.1 Streamlined simulations and model stacking with *TraitTrainR*

**Figure 1.**
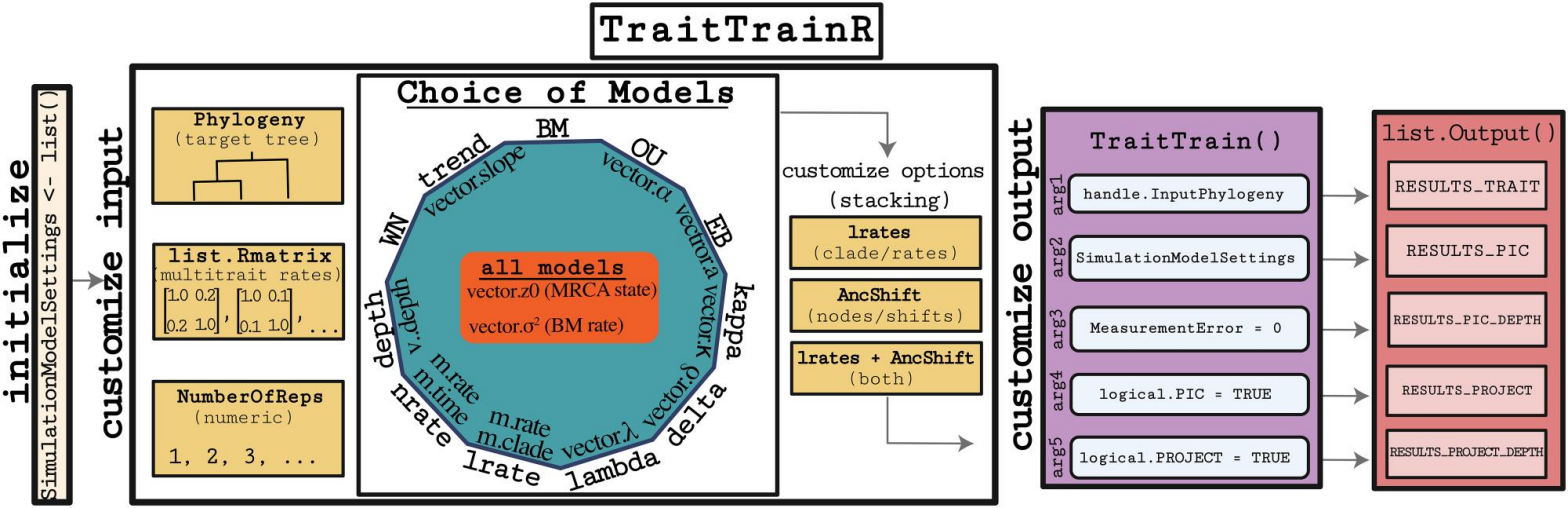
Overview of *TraitTrainR* highlighting its scope and implementation, and options for inputs and outputs. Boxes on the left represent the choice of target phylogeny, the potential for multi-trait simulations for list. Rmatrix, and the number of replicates. The polygon in the center depicts the choice of 11 potential primary models and their associated vectors for evolutionary parameter values. Customized options for the three stack models are shown in the right boxes, followed by output options and the list of output results.

Here, we introduce the package *TraitTrainR*, which is developed in R 4.4.0 and includes a comprehensive suite of functions trailed for organized, flexible, and large-scale simulations of trait evolution (Fig. 1). To facilitate effective and efficient simulation experiments, TraitTrainR incorporates great flexibility in experimental and evolutionary parameters chosen by users (see Section 2.2), automated computation of phylogenetic transformations, and incorporation of measurement error directly into the simulation process.

Models included in *TraitTrainR* (Fig. 1) represent extensions of the BM model, which can be reformulated as phylogenetic transformations that define the outcome of trait evolution as multivariate normal according to the ancestral states, evolutionary parameters, tree topology, and branch lengths, and *TraitTrainR* can also “stack” certain evolutionary models on top of a BM-based model. Specifically, *TraitTrainR* currently includes four different potential “stacking” options: “standard” (BM model or extension only), “lrates”, “AncShifts”, or both “lrates” and “AncShifts” combined. These variations of combined models have not been included within comparable simulation software packages, and thus, allow the user to explore novel modeling scenarios by combining processes. *TraitTrainR* first transforms the input phylogeny according to the primary model type, followed by any stacked model settings. This strategy allows users to simulate trait evolution under more complex evolutionary scenarios that are not available in current simulation software; e.g. a BM model with multiple ancestral shifts, an OU model with localized rate shifts, an EB model with both stacked processes, or perhaps some other combination.

Another advance of *TraitTrainR* is the extensive customization options for both input and output settings, enabling variability in evolutionary models across replicates as well as flexibility in returned output formats. For example, values of the $\sigma^{2}$ rate can be fixed for all replicates (e.g. $\sigma^{2}=1$), or sampled from any number of applicable continuous distributions, including a uniform (with some minimum and maximum), exponential (with some rate), gamma distribution (with some shape and scale), or most any other appropriate distribution, or set of user-specified values. For models that include distinct rate shifts, the user provides a matrix of rate values and shift locations (time intervals or lineages), which permits replicates generated with different shift locations and rates. Likewise, the AncShift model can be specified to include multiple shifts in the ancestral state across the tree, which can be varied or fixed across replicates. Variability in among-trait associations can be incorporated by using a custom among-trait covariance matrix for each replicate for multi-trait simulations. A key advantage of *TraitTrainR* is flexibility in output formats, including: raw trait measurements, phylogenetic independent contrasts (PICs; Felsenstein 1985) computed using the input tree, PICs computed using the input tree scaled to unit depth, phylogenetic transformations using phylogenetic generalized least squares (PGLS) principles (Garland 1992), and PGLS-based transformations using the depth-scaled input tree.

### 2.2 Scope and implementation

The scope of *TraitTrainR* currently includes a total of 44 models, spanning 11 primary models each with four options for model stacking (Fig. 1). The *TraitTrainR* package includes a detailed manual, quick-start guide, and tutorial (see Supplementary Material and the TraitTrainR website), and dependencies include ape (Paradis and Schliep 2019), geiger (Pennell *et al.* 2014), and phytools (Revell 2013) employed for various simulation functions and phylogenetic transformations. The primary inputs required by *TraitTrainR* are a phylogeny for simulation and a ModelSimulationSettings list object that encompasses all user-defined options, including the desired model(s), their parameter values (or vector of parameter values), ancestral states of replicates (or vector of ancestral states), among-trait covariance matrices (for multi-trait simulations), output formats, options for automatically simulating normally distributed measurement error, and information for model stacking if desired. Thus, *TraitTrainR* allows users to specify an array of experimental and evolutionary settings to define the scope of simulation sessions. The primary function that users interact with is termed TraitTrain which requires the input phylogeny and the list ModelSimulationSettings detailed below, alongside any additional options requested by the user.

## 3 Results

### 3.1 Demonstrating TraitTrainR: how well does model selection perform in the presence of trait measurement error?

Selecting a model of trait evolution is fundamental to phylogenetic comparative studies and provides insight into the mode and tempo of trait change expected on a phylogeny (Johnson and Omland 2004). Thus, correctly predicting the true model of evolution for a given studied trait is therefore a critical step toward our understanding of evolutionary and comparative biology (Johnson and Omland 2004, O’Meara 2012, Cornwell and Nakagawa 2017). We applied *TraitTrainR* to investigate the problem of model selection using three empirical phylogenetic case studies: (i) a phylogeny of 76 *Arthropods* (Thomas *et al.* 2020), (ii) 34 *Penicillium* fungi (Steenwyk et al. 2019), and (iii) nine eutherian mammals (Brawand *et al.* 2011). Varying the tree sizes allowed us to explore applications of *TraitTrainR* to large (Athropod), moderate (fungal), and small (primate) trees. We envision many potential applications of *TraitTrainR* (e.g. Bollback 2002, Kutsukake and Innan 2013, Pennell *et al.* 2014), and through these examples we aimed to demonstrate the use of *TraitTrainR* for tackling a critical question: how does model selection perform with and without trait measurement error, and how might that manifest in statistical power (or lack thereof) to find the true evolution model in empirical phylogenetic trait studies?

Each phylogeny was obtained from its respective publication and subsequently used as input by *TraitTrainR* to simulate 10^4^ replicates for each of seven primary focal models (model details provided in Table 1). Specifically, we downloaded the Newick formatted phylogeny from each respective study. Values for all parameters were sampled from probability distributions to incorporate variability in evolutionary processes across replicates and set to reflect the bounds of model parameter values used by the function fitContinuous in geiger (Pennell *et al.* 2014). Distributions for each parameter of the seven models are shown in Table 1. After simulation, maximum likelihood estimation was conducted using fitContinuous to fit the models and calculate Akaike information criterion (AIC; Akaike 1973). That is, for each replicate generated by *TraitTrainR*, a trait dataset was simulated according to one of seven models with varying parameter values (Table 1), and model selection was then conducted using AIC to find the best-fit model. This approach allows us to evaluate whether the true data-generating model would indeed be recovered as the lowest model AIC among the seven candidate models for each replicate. AIC is a gold standard in evolutionary studies for likelihood-based model selection that seeks to balance the goodness of fit (likelihood) with model complexity by penalizing the likelihood by the number of parameters. For example, many studies seek to compare the fit of a simple BM process, or alternatively, a more complex OU model that includes an attraction toward an optimum, or similar questions (e.g. Hansen 1997, Ho and Ané 2014, Rohlfs *et al.* 2014, Blomberg *et al.* 2020, Ho and Dinh 2022, Vu *et al.* 2023). By constructing confusion matrices, we summarized the accuracy of AIC model selection across replicates generated by *TraitTrainR.*

**Table 1.** Summary of the evolutionary models available in the *TraitTrainR*.

Model	Parameters	Evolutionary process	Distributions used in biological applications (Fig. 1)^a^
Brownian motion (BM)	*z*_0_; *σ*^2^	A random-walk model of trait change: $z_{0}$ mean trait value at the root; $\sigma^{2}$ is the rate of trait evolution (variance) (Felsenstein 1985)	*σ* ^2^ ∼ Exp(1); *z*_0_ ∼ *N*(0, 1); (standard normal)
Ornstein–Uhlenbeck (OU)	*z*_0_; *σ*^2^; *α*	Stabilizing selection: *α* is the strength of selection pulling the trait towards a stationary optimal value at the ancestral state *z*_0_ (Butler and King 2004)	*σ* ^2^ ∼ Exp(1); *z*_0_ ∼ *N*(0, 1); *α* ∼ *U*(exp(−500), exp(1))
Early-burst (EB)	*z*_0_; *σ*^2^; *a*	Adaptative radiation: *a* describes the rate at which evolutionary rates decline over time (Harmon *et al.* 2010)	*σ* ^2^ ∼ *Exp*(1); *z*_0_ ∼ *N*(0, 1); *a* ∼ *U*(−5/depth, 10^−6^)
Lambda	*z*_0_; *σ*^2^; *λ*	Phylogenetic signal scaling: *λ* measures the degree to which trait evolution follows the phylogeny (Pagel 1999)	*σ* ^2^ ∼ Exp(1); *z*_0_ ∼ *N*(0, 1); *λ* ∼ *U*(exp(−500), 1)
Delta	*z*_0_; *σ*^2^; δ	Time-dependent evolutionary rate: δ modifies the rate of evolution over time, allowing for acceleration or deceleration (Pagel 1999)	*σ* ^2^ ∼ *Exp*(1); *z*_0_ ∼ *N*(0, 1); δ ∼ *U*(exp(−500), 3)
Kappa	*z*_0_; *σ*^2^; κ	Branch length scaling in phylogeny: κ scales branch lengths, affecting the rate of trait change over evolutionary time (Pagel 1999)	*σ* ^2^ ∼ *Exp*(1); *z*_0_ ∼ *N*(0, 1); κ ∼ *U*(exp(−500), 1)
Rate trend	*z*_0_; *σ*^2^; slope	Linear change in trait evolution over time: slope indicates the direction and magnitude of change in trait evolution over time (Pennell *et al.* 2014)	σ^2^ ∼ *Exp*(1); *z*_0_ ∼ *N*(0, 1); slope ∼ *U*(−100, 100)
White noise	*z*_0_; *σ*^2^	Non-phylogenetic model	
Depth	*z*_0_; *σ*^2^; depth	Scaling tree to a specific evolutionary depth: depth scales the evolutionary time to a specific depth in the tree (Pennell *et al.* 2014)	
Lrates	*z*_0_; *σ*^2^; rates shifts; clades	Rate changes across local clades: rates shifts indicate changes in evolutionary rates across different clades or time intervals (Pennell *et al.* 2014)	
Nrates	*z*_0_; *σ*^2^; rates shifts; intervals	Rate shifts across different time intervals: rates shifts indicate the specific intervals where evolutionary rates change (Pennell *et al.* 2014)	

a The first seven listed models were assessed in our empirical phylogenetic case studies (bottom of Fig. 1) with parameters sampled from the distributions shown in the fourth column.

The framework of *TraitTrainR* incorporates flexibility for multiple trait simulations, and thus, we sought to apply TraitTrainR to also understand the performance of AIC-based model selection when two traits are analyzed using multivariate model selection (Ripplinger and Sullivan 2008, Arnold and Nunn 2010, Chakrabarti and Ghosh 2011, Clavel *et al.* 2015, Brewer *et al.* 2016, Adams and Collyer 2018). Specifically, we used *TraitTrainR* to simulate 10^4^ replicates for each of three models (BM, OU, and EB) for analyses of two traits based on the larger Arthropod phylogeny. As with our seven model applications described above, we also varied the amount of measurement error (variance), and AIC was used to assess the relative fit of each model using the R package mvMORPH (Clavel *et al.* 2015).

**Figure 2.**
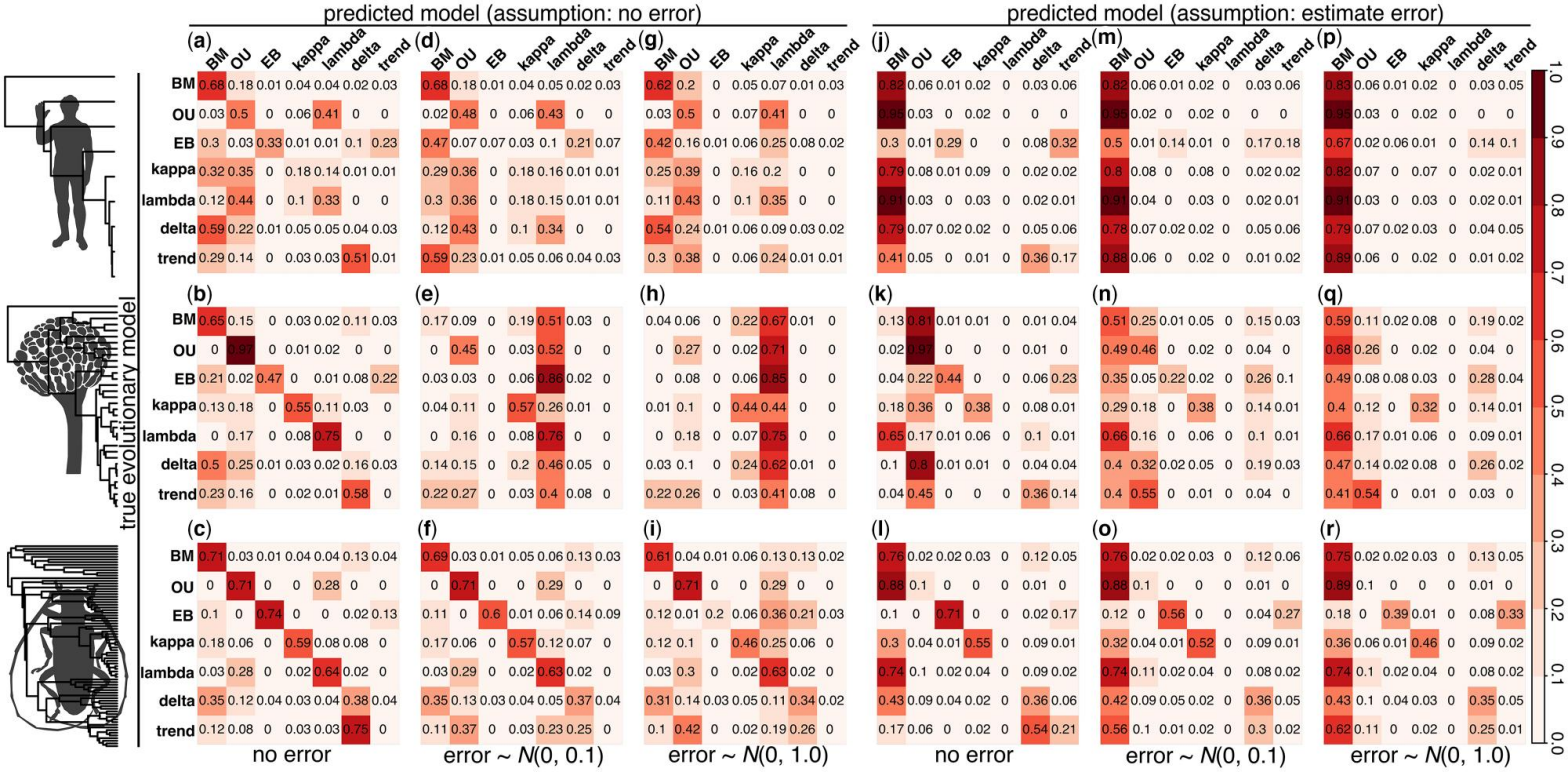
Demonstration an application of *TraitTrainR* to explore aspects of evolutionary model selection with three phylogenetic case studies. Confusion matrices illustrate results of AIC-based model selection for nine mammals (top row), 34 *Penicillium* fungi (middle row), and 74 *Arthropods* (bottom row). Within each case study, results are shown for *TraitTrainR* simulations with no measurement error (a–c; j–l), moderate measurement error (d–f; m–o), and high error (g–i; p–r) and for when error is assumed absent when computing AIC (a–i) and when error is estimated (j–r). Darker shades indicate a higher fraction of replicates under a true model (rows of confusion matrices) assigned to a particular predicted model (columns of confusion matrices).

**Figure 3.**
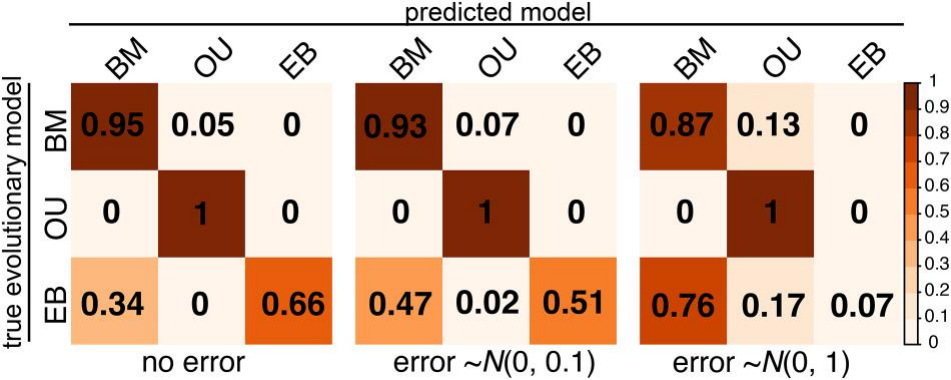
Exploring model selection for two-trait simulations on the *Arthropod* phylogeny. Confusion matrices illustrate results of AIC-based model selection (assuming no error) for *TraitTrainR* simulations without measurement error (left), moderate measurement error (middle), and high error (right). Darker shades indicate a higher fraction of replicates under a true model (rows of confusion matrices) assigned to a particular predicted model (columns of confusion matrices).

Our applications of *TraitTrainR* highlight challenges in selecting the correct model that generated the trait data in all three phylogenetic case studies; these findings are apparent for simulations both with and without measurement error (Fig. 2). Generally, we find the highest accuracy for the largest analyzed tree (bottom row; Fig. 2), which is expected given the increased sample size, followed by the fungal (middle row; Fig. 2) and primate (top row; Fig. 2) case studies, respectively. Yet, measurement error had a major effect on reducing model selection accuracy, and allowing standard error to be estimated during model fitting helped little in many cases (Fig. 2a–i versus j–r). This finding may result from elevated noise-to-signal ratios when introducing measurement error (Ives *et al.* 2007, Felsenstein 2008, Silvestro *et al.* 2015, Bartoszek *et al.* 2024). Estimation of error requires an additional parameter, which may explain why simpler models (i.e. BM) tended to be favored by lower AIC (Fig. 2j–r). However, accuracy to recover the OU model was highest for the medium-sized fungal phylogeny (Fig. 2b), suggesting that tree size is not the only determinant of model selection accuracy, and that model selection accuracy differs depending on the structure of the empirical tree. For this case study, measurement error influenced model selection toward the lambda model (Fig. 2e and h), whereas allowing the model to estimate error resulted in a preference for the simpler BM model (Fig. 2n and q). For these seven model demonstrations, all analyses and case studies struggled to recover the trend model for these single trait simulations. Our two-trait simulations also found evidence of relative reductions in model selection accuracy as measurement error increased (Fig. 3), which reflect similar patterns found in OU-based multivariate studies (Ives *et al.* 2007, Felsenstein 2008, Silvestro *et al.* 2015, Bartoszek *et al.* 2024).

Collectively, our applications reveal inherent challenges of evolutionary model selection and impacts of measurement error (and lack of robustness when such error is estimated), underscoring the applicability of *TraitTrainR* for investigating important statistical and evolutionary questions under realistic expectations of trait data quality. Our findings also highlight the value of simulation studies for investigating the feasibility and power for discerning trait models for any empirical system, which can be examined even prior to data collection. Finally, we also emphasize that our results reflect only a specific set of case studies and explored parameter values (Table 1). Though other studies have identified similar challenges with model selection and interpretation (e.g. Ives *et al.* 2007, Felsenstein 2008, Silvestro *et al.* 2015, Grabowski *et al*. 2023; Bartoszek *et al.* 2024), such findings may be relevant to other datasets, trees, and values of evolutionary parameters. Future studies will clarify the challenges of model selection under various evolutionary and experimental settings.

## Supplementary data

Supplementary data are available at *Bioinformatics Advances* online.

## Conflict of interest

None declared.

## Funding

This research was supported by startup funds from the University of Arkansas, the Arkansas High Performance Computing Center, and National Science Foundation grant IOS-2307044 to T.A.C. and R.A. M.D. was supported by National Institutes of Health grant R35GM128590, and National Science Foundation grants DBI-2130666 and DEB-2302258. D.D.M. was supported by National Science Foundation grant DEB-2110053. R.A. was also supported by funding from the Arkansas BioScience Institute.

## Supplementary Material

vbae196_Supplementary_Data

## Data Availability

The data underlying this article are available in at  https://github.com/radamsRHA/TraitTrainR.
