# Supplementary material for: TraitTrainR: accelerating large-scale simulation under models of continuous trait evolution
Source: Bioinform Adv. 2024 Dec 9;5(1):vbae196. doi: 10.1093/bioadv/vbae196 (PMC11696700; doi:10.1093/bioadv/vbae196)
Supplement: vbae196_Supplementary_Data [file vbae196_supplementary_data.pdf]

Package ‘TraitTrainR’

September 12, 2024

Title Accelerating large-scale simulation under models of continuous trait evolution

Version 0.0.0.9000

Description TraitTrainR is an integrative R package designed to facilitate efficient and organized simulations under phylogenetic models of continuous trait evolution. Please see the associated manual and help files for additional information. Please contact the authors for any and all questions.

License GPL (>= 3)

Encoding UTF-8

Roxygen list(markdown = TRUE)

RoxygenNote 7.3.2

Imports geiger (>= 2.0.11), phytools (>= 2.3.0)

Contents

Simulate.TraitTrain.BM_00 2
Simulate.TraitTrain.BM_STACKED_01 3
Simulate.TraitTrain.BM_STACKED_10 5
Simulate.TraitTrain.BM_STACKED_11 7
Simulate.TraitTrain.delta_00 9
Simulate.TraitTrain.delta_STACKED_01 10
Simulate.TraitTrain.delta_STACKED_10 12
Simulate.TraitTrain.delta_STACKED_11 14
Simulate.TraitTrain.depth_00 16
Simulate.TraitTrain.depth_STACKED_01 17
Simulate.TraitTrain.depth_STACKED_10 19
Simulate.TraitTrain.depth_STACKED_11 20
Simulate.TraitTrain.EB_00 22
Simulate.TraitTrain.EB_STACKED_01 24
Simulate.TraitTrain.EB_STACKED_10 26
Simulate.TraitTrain.EB_STACKED_11 27
Simulate.TraitTrain.kappa_00 29
Simulate.TraitTrain.kappa_STACKED_01 31
Simulate.TraitTrain.kappa_STACKED_10 32
Simulate.TraitTrain.kappa_STACKED_11 34
Simulate.TraitTrain.lambda_00 36
Simulate.TraitTrain.lambda_STACKED_01 38
Simulate.TraitTrain.lambda_STACKED_10 39
Simulate.TraitTrain.lambda_STACKED_11 41

Simulate.TraitTrain.lrate_00 43
Simulate.TraitTrain.lrate_STACKED_01 45
Simulate.TraitTrain.lrate_STACKED_10 47
Simulate.TraitTrain.lrate_STACKED_11 49
Simulate.TraitTrain.nrate_00 51
Simulate.TraitTrain.nrate_STACKED_01 52
Simulate.TraitTrain.nrate_STACKED_10 54
Simulate.TraitTrain.nrate_STACKED_11 56
Simulate.TraitTrain.OU_00 58
Simulate.TraitTrain.OU_STACKED_01 60
Simulate.TraitTrain.OU_STACKED_10 62
Simulate.TraitTrain.OU_STACKED_11 63
Simulate.TraitTrain.trend_00 65
Simulate.TraitTrain.trend_STACKED_01 67
Simulate.TraitTrain.trend_STACKED_10 68
Simulate.TraitTrain.trend_STACKED_11 70
Simulate.TraitTrain.white_00 72
Simulate.TraitTrain.white_STACKED_01 74
Simulate.TraitTrain.white_STACKED_10 75
Simulate.TraitTrain.white_STACKED_11 77
TraitTrain 78

Index **83**

`Simulate.TraitTrain.BM_00`

Simulate.TraitTrain.BM_00: Function to simulate trait data under the BM model

---

Description

This function returns a Matrix of simulated trait data. Species are rows, traits are columns in matrix.TraitData

Usage

```
Simulate.TraitTrain.BM_00(
  handle.Phylogeny,
  numeric.Sig2,
  numeric.AncestralState,
  matrix.R
)
```

Arguments

`handle.Phylogeny`
: Phylogeny used to simulate training data under BM model

`numeric.Sig2`
: Numeric Value of the sigsg (evolutionary rate) parmater of the BM model

`numeric.AncestralState`
: Numerical value of the z0 (ancestral state) parameter of the BM model

`matrix.R`
: Matrix specifying the among trait covariance for p traits. Can also be a single value (1) for a single trait

Value

matrix.TraitData Matrix of simulated trait data. Species are rows, traits are columns

Examples

```
#####################
# load dependencies #
#####################
library(geiger); library(phytools); library(TraitTrainR)

#########################
# get example phylogeny #
#########################
handle.PrimatePhylogeny <- read.tree(text = "((((((((human: 6, chimp:6): 1, gorilla: 7): 7, orangutan: 14): 11,

#############################
# Simulation Model Settings #
#############################
list.SimulationModelSettings <- list() # define an empty model list

##########################
# SET SIMULATION MODEL 1 #
##########################
numeric.NumberTrainingReps <- 2 # same number of replicates for all models in list.SimulationModelSettings
list.Rmatrix <- list(); for (i in 1:numeric.NumberTrainingReps){list.Rmatrix[[i]] <- matrix(c(1, 0, 0, 0, 1, 0,
#list.Rmatrix <- list(); for (i in 1:numeric.NumberTrainingReps){list.Rmatrix[[i]] <- 1} # single trait

#############################
# append to simulation list #
#############################
list.SimulationModelSettings[[1]] <- list(string.SimulationModel = "BM",
                             vector.Sig2 = rexp(n = numeric.NumberTrainingReps, rate = 1),
                            vector.AncestralState = runif(n = numeric.NumberTrainingReps, min = 0, max = 100),
                                          list.Rmatrix = list.Rmatrix)

###########################
# simulate trait under BM #
###########################
Simulate.TraitTrain.BM_00(handle.Phylogeny = handle.PrimatePhylogeny,
                        numeric.Sig2 = list.SimulationModelSettings[[1]]$vector.Sig2[1],
                 numeric.AncestralState = list.SimulationModelSettings[[1]]$vector.AncestralState[1],
                         matrix.R = list.SimulationModelSettings[[1]]$list.Rmatrix[[1]])
```

`Simulate.TraitTrain.BM_STACKED_01`
Simulate.TraitTrain.BM_STACKED_01: Function to simulate trait data under the STACK (AncShifted) BM model

---

Description

This function returns a Matrix of simulated trait data. Species are rows, traits are columns in matrix.TraitData

Usage

```
Simulate.TraitTrain.BM_STACKED_01(
  numeric.NumberOfSpecies,
  handle.Phylogeny,
  numeric.Sig2,
  numeric.AncestralState,
  vector.STACK_AncShiftNode,
  vector.STACK_AncShiftValue,
  matrix.R
)
```

Arguments

`numeric.NumberOfSpecies`
: Numeric counting the number of species

`handle.Phylogeny`
: Phylogeny used to simulate training data under BM model

`numeric.Sig2`
: Numeric Value of the sigsg (evolutionary rate) parmater of the BM model

`numeric.AncestralState`
: Numerical value of the z0 (ancestral state) parameter of the BM model

`vector.STACK_AncShiftNode`
: Vector that includes all nodes for a STACKED AncShift model (on top of BM)

`vector.STACK_AncShiftValue`
: Vector that includes all shift values for each node in vector.STACK_AncShiftNode for a STACKED AncShift model (on top of BM)

`matrix.R`
: Matrix specifying the among trait covariance for p traits. Can also be a single value (1) for a single trait

Value

matrix.TraitData Matrix of simulated trait data. Species are rows, traits are columns

Examples

```
#####################
# load dependencies #
#####################
library(geiger); library(phytools); library(TraitTrainR)

#########################
# get example phylogeny #
#########################
handle.PrimatePhylogeny <- read.tree(text = "((((((((human: 6, chimp:6): 1, gorilla: 7): 7, orangutan: 14): 11,

#############################
# Simulation Model Settings #
#############################
list.SimulationModelSettings <- list() # define an empty model list

##########################
# SET SIMULATION MODEL 1 #
##########################
numeric.NumberTrainingReps <- 1 # same number of replicates for all models in list.SimulationModelSettings
```

```
matrix.STACK_AncShiftNode <- replicate(n = numeric.NumberTrainingReps, sample(size = 2, x = 1:16, replace = F))
matrix.STACK_AncShiftValue <- replicate(n = numeric.NumberTrainingReps , rnorm(n = 2, mean = 10000))
list.Rmatrix <- list(); for (i in 1:numeric.NumberTrainingReps){list.Rmatrix[[i]] <- matrix(c(1, 0, 0, 0, 1, 0,

#############################
# append to simulation list #
#############################
list.SimulationModelSettings[[1]] <- list(string.SimulationModel = "BM",
                              vector.Sig2 = rexp(n = numeric.NumberTrainingReps, rate = 1),
                             vector.AncestralState = runif(n = numeric.NumberTrainingReps, min = 0, max = 100),
                                   matrix.STACK_AncShiftNode = matrix.STACK_AncShiftNode,
                                 matrix.STACK_AncShiftValue = matrix.STACK_AncShiftValue,
                                           list.Rmatrix = list.Rmatrix)

#########################################################
# simulate under BM model with STACKED (BM + AncSHift) #
#########################################################
Simulate.TraitTrain.BM_STACKED_01(numeric.NumberOfSpecies = length(handle.PrimatePhylogeny$tip.label),
                        handle.Phylogeny = handle.PrimatePhylogeny, numeric.Sig2 = list.SimulationModelSetting
                        numeric.AncestralState = list.SimulationModelSettings[[1]]$vector.AncestralState[1],
                        vector.STACK_AncShiftNode = list.SimulationModelSettings[[1]]$matrix.STACK_AncShiftNo
                        vector.STACK_AncShiftValue = list.SimulationModelSettings[[1]]$matrix.STACK_AncShift\
                           matrix.R = list.SimulationModelSettings[[1]]$list.Rmatrix[[1]])
```

`Simulate.TraitTrain.BM_STACKED_10`

Simulate.TraitTrain.BM_STACKED_10: Function to simulate trait data under the STACK (lrates) BM model

---

Description

This function returns a Matrix of simulated trait data. Species are rows, traits are columns in matrix.TraitData

Usage

```
Simulate.TraitTrain.BM_STACKED_10(
  handle.Phylogeny,
  numeric.Sig2,
  numeric.AncestralState,
  vector.STACK_lrate_nodes,
  vector.STACK_lrate_rates,
  matrix.R
)
```

Arguments

`handle.Phylogeny`
: Phylogeny used to simulate training data under BM model

`numeric.Sig2`
: Numeric Value of the sigsg (evolutionary rate) parmater of the BM model

`numeric.AncestralState`
: Numerical value of the z0 (ancestral state) parameter of the BM model

`vector.STACK_lrate_nodes`
: Vector that includes all nodes for a STACKED lrates model (on top of BM)

`vector.STACK_lrate_rates`
: Vector that includes all rate values for each node in vector.STACK_lrate_nodes for a STACKED lrates model (on top of BM)

`matrix.R`
: Matrix specifying the among trait covariance for p traits. Can also be a single value (1) for a single trait

Value

matrix.TraitData Matrix of simulated trait data. Species are rows, traits are columns

Examples

```
#####################
# load dependencies #
#####################
library(geiger); library(phytools); library(TraitTrainR)

#########################
# get example phylogeny #
#########################
handle.PrimatePhylogeny <- read.tree(text = "((((((((human: 6, chimp:6): 1, gorilla: 7): 7, orangutan: 14): 11,

#############################
# Simulation Model Settings #
#############################
list.SimulationModelSettings <- list() # define an empty model list

##########################
# SET SIMULATION MODEL 1 #
##########################
numeric.NumberTrainingReps <- 2 # same number of replicates for all models in list.SimulationModelSettings
matrix.STACK_lrate_nodes <- replicate(n = numeric.NumberTrainingReps, sample(size = 2, x = 1:16, replace = F))
matrix.STACK_lrate_rates <- replicate(n = numeric.NumberTrainingReps , runif(n = 2, min = 0, max = 100))
list.Rmatrix <- list(); for (i in 1:numeric.NumberTrainingReps){list.Rmatrix[[i]] <- matrix(c(1, 0, 0, 0, 1, 0,
#list.Rmatrix <- list(); for (i in 1:numeric.NumberTrainingReps){list.Rmatrix[[i]] <- 1} # single trait

#############################
# append to simulation list #
#############################
list.SimulationModelSettings[[1]] <- list(string.SimulationModel = "BM",
                             vector.Sig2 = rexp(n = numeric.NumberTrainingReps, rate = 1),
                           vector.AncestralState = runif(n = numeric.NumberTrainingReps, min = 0, max = 100),
                                   matrix.STACK_lrate_nodes = matrix.STACK_lrate_nodes,
                                   matrix.STACK_lrate_rates = matrix.STACK_lrate_rates,
                                        list.Rmatrix = list.Rmatrix)

#########################################################
# simulate trait under BM with STACKED 10 (BM + lrates) #
#########################################################
Simulate.TraitTrain.BM_STACKED_10(handle.Phylogeny = handle.PrimatePhylogeny,
                        numeric.Sig2 = list.SimulationModelSettings[[1]]$vector.Sig2[1],
                      numeric.AncestralState = list.SimulationModelSettings[[1]]$vector.AncestralState[1],
                      vector.STACK_lrate_nodes = list.SimulationModelSettings[[1]]$matrix.STACK_lrate_nodes
                      vector.STACK_lrate_rates = list.SimulationModelSettings[[1]]$matrix.STACK_lrate_rates
```

```
                    matrix.R = list.SimulationModelSettings[[1]]$list.Rmatrix[[1]])
```

Simulate.TraitTrain.BM_STACKED_11

Simulate.TraitTrain.BM_STACKED_11: Function to simulate trait data under the STACK (lrates + shifted) BM model

Description

This function returns a Matrix of simulated trait data. Species are rows, traits are columns in matrix.TraitData

Usage

```
Simulate.TraitTrain.BM_STACKED_11(
  numeric.NumberOfSpecies,
  handle.Phylogeny,
  numeric.Sig2,
  numeric.AncestralState,
  vector.STACK_lrate_nodes,
  vector.STACK_lrate_rates,
  vector.STACK_AncShiftNode,
  vector.STACK_AncShiftValue,
  matrix.R
)
```

Arguments

`numeric.NumberOfSpecies`
: Numeric counting the number of species

`handle.Phylogeny`
: Phylogeny used to simulate training data under BM model

`numeric.Sig2`
: Numeric Value of the sigsg (evolutionary rate) parmater of the BM model

`numeric.AncestralState`
: Numerical value of the z0 (ancestral state) parameter of the BM model

`vector.STACK_lrate_nodes`
: Vector that includes all nodes for a STACKED lrates model (on top of BM)

`vector.STACK_lrate_rates`
: Vector that includes all rate values for each node in vector.STACK_lrate_nodes for a STACKED lrates model (on top of BM)

`vector.STACK_AncShiftNode`
: Vector that includes all nodes for a STACKED AncShift model (on top of BM)

`vector.STACK_AncShiftValue`
: Vector that includes all shift values for each node in vector.STACK_AncShiftNode for a STACKED AncShift model (on top of BM)

`matrix.R`
: Matrix specifying the among trait covariance for p traits. Can also be a single value (1) for a single trait

Value

matrix.TraitData Matrix of simulated trait data. Species are rows, traits are columns

Examples

```
#####################
# load dependencies #
#####################
library(geiger); library(phytools); library(TraitTrainR)

#########################
# get example phylogeny #
#########################
handle.PrimatePhylogeny <- read.tree(text = "((((((((human: 6, chimp:6): 1, gorilla: 7): 7, orangutan: 14): 11,

#############################
# Simulation Model Settings #
#############################
list.SimulationModelSettings <- list() # define an empty model list

##########################
# SET SIMULATION MODEL 1 #
##########################
numeric.NumberTrainingReps <- 10 # same number of replicates for all models in list.SimulationModelSettings
matrix.STACK_lrate_nodes <- matrix(replicate(n = numeric.NumberTrainingReps, sample(size = 1, x = 1:16, replac
matrix.STACK_lrate_rates <- matrix(replicate(n = numeric.NumberTrainingReps , runif(n = 1, min = 0, max = 100))
matrix.STACK_AncShiftNode <- replicate(n = numeric.NumberTrainingReps, sample(size = 2, x = 1:16, replace = F))
matrix.STACK_AncShiftValue <- replicate(n = numeric.NumberTrainingReps , rnorm(n = 2, mean = 10000))
list.Rmatrix <- list(); for (i in 1:numeric.NumberTrainingReps){list.Rmatrix[[i]] <- matrix(c(1, 0, 0, 0, 1, 0,
#list.Rmatrix <- list(); for (i in 1:numeric.NumberTrainingReps){list.Rmatrix[[i]] <- 1} # single trait

#############################
# append to simulation list #
#############################
list.SimulationModelSettings[[1]] <- list(string.SimulationModel = "BM",
                              vector.Sig2 = rexp(n = numeric.NumberTrainingReps, rate = 1),
                             vector.AncestralState = runif(n = numeric.NumberTrainingReps, min = 0, max = 100),
                                     matrix.STACK_lrate_nodes = matrix.STACK_lrate_nodes,
                                     matrix.STACK_lrate_rates = matrix.STACK_lrate_rates,
                                   matrix.STACK_AncShiftNode = matrix.STACK_AncShiftNode,
                                 matrix.STACK_AncShiftValue = matrix.STACK_AncShiftValue,
                                           list.Rmatrix = list.Rmatrix)

####################################################################
# simulate under BM model with STACKED 11 (BM + lrates + AncShift) #
####################################################################
Simulate.TraitTrain.BM_STACKED_11(numeric.NumberOfSpecies = length(handle.PrimatePhylogeny$tip.label),
                                   handle.Phylogeny = handle.PrimatePhylogeny,
                         numeric.Sig2 = list.SimulationModelSettings[[1]]$vector.Sig2[1],
                       numeric.AncestralState = list.SimulationModelSettings[[1]]$vector.AncestralState[1],
                       vector.STACK_lrate_nodes = list.SimulationModelSettings[[1]]$matrix.STACK_lrate_nodes
                       vector.STACK_lrate_rates = list.SimulationModelSettings[[1]]$matrix.STACK_lrate_rates
                       vector.STACK_AncShiftNode = list.SimulationModelSettings[[1]]$matrix.STACK_AncShiftNo
                       vector.STACK_AncShiftValue = list.SimulationModelSettings[[1]]$matrix.STACK_AncShiftV
                          matrix.R = list.SimulationModelSettings[[1]]$list.Rmatrix[[1]])
```

Simulate.TraitTrain.delta_00

Simulate.TraitTrain.delta_00: Function to simulate trait data under the delta model

Description

This function returns a Matrix of simulated trait data. Species are rows, traits are columns in matrix.TraitData

Usage

```
Simulate.TraitTrain.delta_00(
  handle.Phylogeny,
  numeric.Sig2,
  numeric.delta,
  numeric.AncestralState,
  matrix.R
)
```

Arguments

`handle.Phylogeny`
: Phylogeny used to simulate training data under delta model

`numeric.Sig2`
: Numeric Value of the sigsg (evolutionary rate) parmater of the delta model

`numeric.delta`
: Numeric value of the delta parameter of the delta model

`numeric.AncestralState`
: Numerical value of the z0 (ancestral state) parameter of the delta model

`matrix.R`
: Matrix specifying the among trait covariance for p traits. Can also be a single value (1) for a single trait

`numeric.NumberOfSpecies`
: Numeric counting the number of species

Value

matrix.TraitData Matrix of simulated trait data. Species are rows, traits are columns

Examples

```
#####################
# load dependencies #
#####################
library(geiger); library(phytools); library(TraitTrainR)

#########################
# get example phylogeny #
#########################
handle.PrimatePhylogeny <- read.tree(text = "((((((((human: 6, chimp:6): 1, gorilla: 7): 7, orangutan: 14): 11,

#############################
# Simulation Model Settings #
```

```
##############################
list.SimulationModelSettings <- list() # define an empty model list

##########################
# SET SIMULATION MODEL 1 #
##########################
numeric.NumberTrainingReps <- 10 # same number of replicates for all models in list.SimulationModelSettings
list.Rmatrix <- list(); for (i in 1:numeric.NumberTrainingReps){list.Rmatrix[[i]] <- matrix(c(1, 0, 0, 0, 1, 0,
#list.Rmatrix <- list(); for (i in 1:numeric.NumberTrainingReps){list.Rmatrix[[i]] <- 1} # single trait

##############################
# append to simulation list #
##############################
list.SimulationModelSettings[[1]] <- list(string.SimulationModel = "delta",
                             vector.Sig2 = rexp(n = numeric.NumberTrainingReps, rate = 1),
                            vector.AncestralState = runif(n = numeric.NumberTrainingReps, min = 0, max = 100),
                            vector.delta = runif(n = numeric.NumberTrainingReps, min = exp(-500), max = 3),
                                          list.Rmatrix = list.Rmatrix)

##############################
# simulate under delta model #
##############################
Simulate.TraitTrain.delta_00(handle.Phylogeny = handle.PrimatePhylogeny,
                        numeric.Sig2 = list.SimulationModelSettings[[1]]$vector.Sig2[1],
                   numeric.AncestralState = list.SimulationModelSettings[[1]]$vector.AncestralState[1],
                       numeric.delta= list.SimulationModelSettings[[1]]$vector.delta[1],
                         matrix.R = list.SimulationModelSettings[[1]]$list.Rmatrix[[1]])
```

Simulate.TraitTrain.delta_STACKED_01 *Simulate.TraitTrain.delta_STACKED_01: Function to simulate trait data under the STACK (+ AncShift) delta model*

Description

This function returns a Matrix of simulated trait data. Species are rows, traits are columns in matrix.TraitData

Usage

```
Simulate.TraitTrain.delta_STACKED_01(
  numeric.NumberOfSpecies,
  handle.Phylogeny,
  numeric.Sig2,
  numeric.delta,
  numeric.AncestralState,
  vector.STACK_AncShiftNode,
  vector.STACK_AncShiftValue,
  matrix.R
)
```

Arguments

`numeric.NumberOfSpecies`
: Numeric counting the number of species

`handle.Phylogeny`
: Phylogeny used to simulate training data under delta model

`numeric.Sig2`
: Numeric Value of the sigsg (evolutionary rate) parmater of the delta model

`numeric.delta`
: Numeric value of the delta parameter of the delta model

`numeric.AncestralState`
: Numerical value of the z0 (ancestral state) parameter of the delta model

`vector.STACK_AncShiftNode`
: Vector that includes all nodes for a STACKED AncShift model (on top of delta)

`vector.STACK_AncShiftValue`
: Vector that includes all shift values for each node in vector.STACK_AncShiftNode for a STACKED AncShift model (on top of delta)

`matrix.R`
: Matrix specifying the among trait covariance for p traits. Can also be a single value (1) for a single trait

Value

matrix.TraitData Matrix of simulated trait data. Species are rows, traits are columns

Examples

```
#####################
# load dependencies #
#####################
library(geiger); library(phytools); library(TraitTrainR)

#########################
# get example phylogeny #
#########################
handle.PrimatePhylogeny <- read.tree(text = "((((((((human: 6, chimp:6): 1, gorilla: 7): 7, orangutan: 14): 11,

#############################
# Simulation Model Settings #
#############################
list.SimulationModelSettings <- list() # define an empty model list

##########################
# SET SIMULATION MODEL 1 #
##########################
numeric.NumberTrainingReps <- 10 # same number of replicates for all models in list.SimulationModelSettings
matrix.STACK_AncShiftNode <- replicate(n = numeric.NumberTrainingReps, sample(size = 2, x = 1:16, replace = F))
matrix.STACK_AncShiftValue <- replicate(n = numeric.NumberTrainingReps , rnorm(n = 2, mean = 10000))
list.Rmatrix <- list(); for (i in 1:numeric.NumberTrainingReps){list.Rmatrix[[i]] <- matrix(c(1, 0, 0, 0, 1, 0,
#list.Rmatrix <- list(); for (i in 1:numeric.NumberTrainingReps){list.Rmatrix[[i]] <- 1} # single trait

#############################
# append to simulation list #
#############################
list.SimulationModelSettings[[1]] <- list(string.SimulationModel = "delta",
                              vector.Sig2 = rexp(n = numeric.NumberTrainingReps, rate = 1),
                             vector.AncestralState = runif(n = numeric.NumberTrainingReps, min = 0, max = 100),
```

```
                                    matrix.STACK_AncShiftNode = matrix.STACK_AncShiftNode,
                                  matrix.STACK_AncShiftValue = matrix.STACK_AncShiftValue,
                              vector.delta = runif(n = numeric.NumberTrainingReps, min = exp(-500), max = 3),
                                          list.Rmatrix = list.Rmatrix)

##################################################################
# simulate under delta model with STACKED 01 (delta + AncShift) #
##################################################################
Simulate.TraitTrain.delta_STACKED_01(numeric.NumberOfSpecies = length(handle.PrimatePhylogeny$tip.label),
                                      handle.Phylogeny = handle.PrimatePhylogeny,
                        numeric.Sig2 = list.SimulationModelSettings[[1]]$vector.Sig2[1],
                        numeric.AncestralState = list.SimulationModelSettings[[1]]$vector.AncestralState[1]
                        vector.STACK_AncShiftNode = list.SimulationModelSettings[[1]]$matrix.STACK_AncShif
                        vector.STACK_AncShiftValue = list.SimulationModelSettings[[1]]$matrix.STACK_AncShi
                        numeric.delta= list.SimulationModelSettings[[1]]$vector.delta[1],
                         matrix.R = list.SimulationModelSettings[[1]]$list.Rmatrix[[1]])
```

`Simulate.TraitTrain.delta_STACKED_10`

Simulate.TraitTrain.delta_STACKED_10: Function to simulate trait data under the STACK (+lrates) delta model

---

Description

This function returns a Matrix of simulated trait data. Species are rows, traits are columns in matrix.TraitData

Usage

```
Simulate.TraitTrain.delta_STACKED_10(
  handle.Phylogeny,
  numeric.Sig2,
  numeric.delta,
  numeric.AncestralState,
  vector.STACK_lrate_nodes,
  vector.STACK_lrate_rates,
  matrix.R
)
```

Arguments

`handle.Phylogeny`
: Phylogeny used to simulate training data under delta model

`numeric.Sig2`
: Numeric Value of the sigsg (evolutionary rate) parmater of the delta model

`numeric.delta`
: Numeric value of the delta parameter of the delta model

`numeric.AncestralState`
: Numerical value of the z0 (ancestral state) parameter of the delta model

`vector.STACK_lrate_nodes`
: Vector that includes all nodes for a STACKED lrates model (on top of delta)

`vector.STACK_lrate_rates`
: Vector that includes all rate values for each node in vector.STACK_lrate_nodes for a STACKED lrates model (on top of delta)

`matrix.R`
: Matrix specifying the among trait covariance for p traits. Can also be a single value (1) for a single trait

`numeric.NumberOfSpecies`
: Numeric counting the number of species

Value

matrix.TraitData Matrix of simulated trait data. Species are rows, traits are columns

Examples

```
#####################
# load dependencies #
#####################
library(geiger); library(phytools); library(TraitTrainR)

#########################
# get example phylogeny #
#########################
handle.PrimatePhylogeny <- read.tree(text = "((((((((human: 6, chimp:6): 1, gorilla: 7): 7, orangutan: 14): 11,

#############################
# Simulation Model Settings #
#############################
list.SimulationModelSettings <- list() # define an empty model list

##########################
# SET SIMULATION MODEL 1 #
##########################
numeric.NumberTrainingReps <- 10 # same number of replicates for all models in list.SimulationModelSettings
matrix.STACK_lrate_nodes <- matrix(replicate(n = numeric.NumberTrainingReps, sample(size = 1, x = 1:16, replac
matrix.STACK_lrate_rates <- matrix(replicate(n = numeric.NumberTrainingReps , runif(n = 1, min = 0, max = 100))
list.Rmatrix <- list(); for (i in 1:numeric.NumberTrainingReps){list.Rmatrix[[i]] <- matrix(c(1, 0, 0, 0, 1, 0,
#list.Rmatrix <- list(); for (i in 1:numeric.NumberTrainingReps){list.Rmatrix[[i]] <- 1} # single trait

#############################
# append to simulation list #
#############################
list.SimulationModelSettings[[1]] <- list(string.SimulationModel = "delta",
                             vector.Sig2 = rexp(n = numeric.NumberTrainingReps, rate = 1),
                            vector.AncestralState = runif(n = numeric.NumberTrainingReps, min = 0, max = 100),
                                    matrix.STACK_lrate_nodes = matrix.STACK_lrate_nodes,
                                    matrix.STACK_lrate_rates = matrix.STACK_lrate_rates,
                            vector.delta = runif(n = numeric.NumberTrainingReps, min = exp(-500), max = 3),
                                          list.Rmatrix = list.Rmatrix)

#################################################################
# simulate under delta model with STACKED 10 (delta + lrates ) #
#################################################################
Simulate.TraitTrain.delta_STACKED_10(handle.Phylogeny = handle.PrimatePhylogeny,
                        numeric.Sig2 = list.SimulationModelSettings[[1]]$vector.Sig2[1],
                        numeric.AncestralState = list.SimulationModelSettings[[1]]$vector.AncestralState[1]
                        vector.STACK_lrate_nodes = list.SimulationModelSettings[[1]]$matrix.STACK_lrate_noc
```

```
vector.STACK_lrate_rates = list.SimulationModelSettings[[1]]$matrix.STACK_lrate_ra
numeric.delta= list.SimulationModelSettings[[1]]$vector.delta[1],
 matrix.R = list.SimulationModelSettings[[1]]$list.Rmatrix[[1]])
```

Simulate.TraitTrain.delta_STACKED_11

Simulate.TraitTrain.delta_STACKED_11: Function to simulate trait data under the STACK (lrates + shifted) delta model

---

Description

This function returns a Matrix of simulated trait data. Species are rows, traits are columns in matrix.TraitData

Usage

```
Simulate.TraitTrain.delta_STACKED_11(
  numeric.NumberOfSpecies,
  handle.Phylogeny,
  numeric.Sig2,
  numeric.delta,
  numeric.AncestralState,
  vector.STACK_lrate_nodes,
  vector.STACK_lrate_rates,
  vector.STACK_AncShiftNode,
  vector.STACK_AncShiftValue,
  matrix.R
)
```

Arguments

`numeric.NumberOfSpecies`
: Numeric counting the number of species

`handle.Phylogeny`
: Phylogeny used to simulate training data under delta model

`numeric.Sig2`
: Numeric Value of the sigsg (evolutionary rate) parmater of the delta model

`numeric.delta`
: Numeric value of the delta parameter of the delta model

`numeric.AncestralState`
: Numerical value of the z0 (ancestral state) parameter of the delta model

`vector.STACK_lrate_nodes`
: Vector that includes all nodes for a STACKED lrates model (on top of delta)

`vector.STACK_lrate_rates`
: Vector that includes all rate values for each node in vector.STACK_lrate_nodes for a STACKED lrates model (on top of delta)

`vector.STACK_AncShiftNode`
: Vector that includes all nodes for a STACKED AncShift model (on top of delta)

`vector.STACK_AncShiftValue`
: Vector that includes all shift values for each node in vector.STACK_AncShiftNode for a STACKED AncShift model (on top of delta)

`matrix.R`
: Matrix specifying the among trait covariance for p traits. Can also be a single value (1) for a single trait

Value

matrix.TraitData Matrix of simulated trait data. Species are rows, traits are columns

Examples

```
#####################
# load dependencies #
#####################
library(geiger); library(phytools); library(TraitTrainR)

#########################
# get example phylogeny #
#########################
handle.PrimatePhylogeny <- read.tree(text = "((((((((human: 6, chimp:6): 1, gorilla: 7): 7, orangutan: 14): 11,

##############################
# Simulation Model Settings #
##############################
list.SimulationModelSettings <- list() # define an empty model list

##########################
# SET SIMULATION MODEL 1 #
##########################
numeric.NumberTrainingReps <- 10 # same number of replicates for all models in list.SimulationModelSettings
matrix.STACK_lrate_nodes <- matrix(replicate(n = numeric.NumberTrainingReps, sample(size = 1, x = 1:16, replac
matrix.STACK_lrate_rates <- matrix(replicate(n = numeric.NumberTrainingReps , runif(n = 1, min = 0, max = 100))
matrix.STACK_AncShiftNode <- replicate(n = numeric.NumberTrainingReps, sample(size = 2, x = 1:16, replace = F))
matrix.STACK_AncShiftValue <- replicate(n = numeric.NumberTrainingReps , rnorm(n = 2, mean = 10000))
list.Rmatrix <- list(); for (i in 1:numeric.NumberTrainingReps){list.Rmatrix[[i]] <- matrix(c(1, 0, 0, 0, 1, 0,
#list.Rmatrix <- list(); for (i in 1:numeric.NumberTrainingReps){list.Rmatrix[[i]] <- 1} # single trait

##############################
# append to simulation list #
##############################
list.SimulationModelSettings[[1]] <- list(string.SimulationModel = "delta",
                             vector.Sig2 = rexp(n = numeric.NumberTrainingReps, rate = 1),
                            vector.AncestralState = runif(n = numeric.NumberTrainingReps, min = 0, max = 100),
                                    matrix.STACK_lrate_nodes = matrix.STACK_lrate_nodes,
                                    matrix.STACK_lrate_rates = matrix.STACK_lrate_rates,
                                  matrix.STACK_AncShiftNode = matrix.STACK_AncShiftNode,
                                matrix.STACK_AncShiftValue = matrix.STACK_AncShiftValue,
                            vector.delta = runif(n = numeric.NumberTrainingReps, min = exp(-500), max = 3),
                                          list.Rmatrix = list.Rmatrix)

############################################################################
# simulate under delta model with STACKED 11 (delta + lrates + AncShift) #
############################################################################
Simulate.TraitTrain.delta_STACKED_11(numeric.NumberOfSpecies = length(handle.PrimatePhylogeny$tip.label),
                                     handle.Phylogeny = handle.PrimatePhylogeny,
                         numeric.Sig2 = list.SimulationModelSettings[[1]]$vector.Sig2[1],
                         numeric.AncestralState = list.SimulationModelSettings[[1]]$vector.AncestralState[1]
                         vector.STACK_lrate_nodes = list.SimulationModelSettings[[1]]$matrix.STACK_lrate_noc
                         vector.STACK_lrate_rates = list.SimulationModelSettings[[1]]$matrix.STACK_lrate_rat
                         vector.STACK_AncShiftNode = list.SimulationModelSettings[[1]]$matrix.STACK_AncShift
                         vector.STACK_AncShiftValue = list.SimulationModelSettings[[1]]$matrix.STACK_AncShif
                         numeric.delta= list.SimulationModelSettings[[1]]$vector.delta[1],
```

```
matrix.R = list.SimulationModelSettings[[1]]$list.Rmatrix[[1]])
```

`Simulate.TraitTrain.depth_00` *Simulate.TraitTrain.depth_00: Function to simulate trait data under the depth model*

Description

This function returns a Matrix of simulated trait data. Species are rows, traits are columns in matrix.TraitData

Usage

```
Simulate.TraitTrain.depth_00(
  handle.Phylogeny,
  numeric.depth,
  numeric.AncestralState,
  matrix.R
)
```

Arguments

`handle.Phylogeny`
: Phylogeny used to simulate training data under depth model

`numeric.AncestralState`
: Numerical value of the z0 (ancestral state) parameter of the depth model

`matrix.R`
: Matrix specifying the among trait covariance for p traits. Can also be a single value (1) for a single trait

`vector.STACK_lrate_nodes`
: Vector that includes all nodes for a STACKED lrates model (on top of depth)

`vector.STACK_lrate_rates`
: Vector that includes all rate values for each node in vector.STACK_lrate_nodes for a STACKED lrates model (on top of depth)

`vector.STACK_AncShiftNode`
: Vector that includes all nodes for a STACKED AncShift model (on top of depth)

`vector.STACK_AncShiftValue`
: Vector that includes all shift values for each node in vector.STACK_AncShiftNode for a STACKED AncShift model (on top of depth)

Value

matrix.TraitData Matrix of simulated trait data. Species are rows, traits are columns

Examples

```
#####################
# load dependencies #
#####################
library(geiger); library(phytools); library(TraitTrainR)

#########################
# get example phylogeny #
#########################
handle.PrimatePhylogeny <- read.tree(text = "((((((((human: 6, chimp:6): 1, gorilla: 7): 7, orangutan: 14): 11,

#############################
# Simulation Model Settings #
#############################
list.SimulationModelSettings <- list() # define an empty model list

##########################
# SET SIMULATION MODEL 1 #
##########################
numeric.NumberTrainingReps <- 10 # same number of replicates for all models in list.SimulationModelSettings
list.Rmatrix <- list(); for (i in 1:numeric.NumberTrainingReps){list.Rmatrix[[i]] <- matrix(c(1, 0, 0, 0, 1, 0,
#list.Rmatrix <- list(); for (i in 1:numeric.NumberTrainingReps){list.Rmatrix[[i]] <- 1} # single trait

#############################
# append to simulation list #
#############################
list.SimulationModelSettings[[1]] <- list(string.SimulationModel = "depth",
                            vector.AncestralState = runif(n = numeric.NumberTrainingReps, min = 0, max = 100),
                            vector.Depth = runif(n = numeric.NumberTrainingReps, min = 0, max = 10),
                                          list.Rmatrix = list.Rmatrix)

##############################
# simulate under depth model #
##############################
Simulate.TraitTrain.depth_00(numeric.NumberOfSpecies = length(handle.PrimatePhylogeny$tip.label),
                             handle.Phylogeny = handle.PrimatePhylogeny,
                   numeric.AncestralState = list.SimulationModelSettings[[1]]$vector.AncestralState[1],
                      numeric.depth = list.SimulationModelSettings[[1]]$vector.Depth[1],
                         matrix.R = list.SimulationModelSettings[[1]]$list.Rmatrix[[1]])
```

`Simulate.TraitTrain.depth_STACKED_01`
Simulate.TraitTrain.depth_STACKED_01: Function to simulate trait data under the STACK (+AncShifted) depth model

---

Description

This function returns a Matrix of simulated trait data. Species are rows, traits are columns in matrix.TraitData

Usage

```
Simulate.TraitTrain.depth_STACKED_01(
  numeric.NumberOfSpecies,
```

```
    handle.Phylogeny,
    numeric.depth,
    numeric.AncestralState,
    vector.STACK_AncShiftNode,
    vector.STACK_AncShiftValue,
    matrix.R
  )
```

Arguments

`numeric.NumberOfSpecies`
: Numeric counting the number of species

`handle.Phylogeny`
: Phylogeny used to simulate training data under depth model

`numeric.AncestralState`
: Numerical value of the z0 (ancestral state) parameter of the depth model

`vector.STACK_AncShiftNode`
: Vector that includes all nodes for a STACKED AncShift model (on top of depth)

`vector.STACK_AncShiftValue`
: Vector that includes all shift values for each node in vector.STACK_AncShiftNode for a STACKED AncShift model (on top of depth)

`matrix.R`
: Matrix specifying the among trait covariance for p traits. Can also be a single value (1) for a single trait

Value

matrix.TraitData Matrix of simulated trait data. Species are rows, traits are columns

Examples

```
#####################
# load dependencies #
#####################
library(geiger); library(phytools); library(TraitTrainR)

#########################
# get example phylogeny #
#########################
handle.PrimatePhylogeny <- read.tree(text = "((((((((human: 6, chimp:6): 1, gorilla: 7): 7, orangutan: 14): 11,

#############################
# Simulation Model Settings #
#############################
list.SimulationModelSettings <- list() # define an empty model list

##########################
# SET SIMULATION MODEL 1 #
##########################
numeric.NumberTrainingReps <- 10 # same number of replicates for all models in list.SimulationModelSettings
matrix.STACK_AncShiftNode <- replicate(n = numeric.NumberTrainingReps, sample(size = 2, x = 1:16, replace = F))
matrix.STACK_AncShiftValue <- replicate(n = numeric.NumberTrainingReps , rnorm(n = 2, mean = 10000))
list.Rmatrix <- list(); for (i in 1:numeric.NumberTrainingReps){list.Rmatrix[[i]] <- matrix(c(1, 0, 0, 0, 1, 0,
#list.Rmatrix <- list(); for (i in 1:numeric.NumberTrainingReps){list.Rmatrix[[i]] <- 1} # single trait
```

```
##############################
# append to simulation list #
##############################
list.SimulationModelSettings[[1]] <- list(string.SimulationModel = "depth",
                            vector.AncestralState = runif(n = numeric.NumberTrainingReps, min = 0, max = 100),
                            matrix.STACK_AncShiftNode = matrix.STACK_AncShiftNode, matrix.STACK_AncShiftValu
                            vector.Depth = runif(n = numeric.NumberTrainingReps, min = 0, max = 10),
                                          list.Rmatrix = list.Rmatrix)

#################################################################
# simulate under depth model with STACKED 01 (Depth + AncShift) #
#################################################################
Simulate.TraitTrain.depth_STACKED_01(numeric.NumberOfSpecies = length(handle.PrimatePhylogeny$tip.label),
                                     handle.Phylogeny = handle.PrimatePhylogeny,
                         numeric.AncestralState = list.SimulationModelSettings[[1]]$vector.AncestralState[1]
                         numeric.depth = list.SimulationModelSettings[[1]]$vector.Depth[1],
                         vector.STACK_AncShiftNode = list.SimulationModelSettings[[1]]$matrix.STACK_AncShift
                         vector.STACK_AncShiftValue = list.SimulationModelSettings[[1]]$matrix.STACK_AncShi
                         matrix.R =  list.SimulationModelSettings[[1]]$list.Rmatrix[[1]])
```

Simulate.TraitTrain.depth_STACKED_10

Simulate.TraitTrain.depth_STACKED_10: Function to simulate trait data under the STACK (+lrates) depth model

---

Description

This function returns a Matrix of simulated trait data. Species are rows, traits are columns in matrix.TraitData

Usage

```
Simulate.TraitTrain.depth_STACKED_10(
  handle.Phylogeny,
  numeric.depth,
  numeric.AncestralState,
  vector.STACK_lrate_nodes,
  vector.STACK_lrate_rates,
  matrix.R
)
```

Arguments

`handle.Phylogeny`
: Phylogeny used to simulate training data under depth model

`numeric.AncestralState`
: Numerical value of the z0 (ancestral state) parameter of the depth model

`vector.STACK_lrate_nodes`
: Vector that includes all nodes for a STACKED lrates model (on top of depth)

`vector.STACK_lrate_rates`
: Vector that includes all rate values for each node in vector.STACK_lrate_nodes for a STACKED lrates model (on top of depth)

`matrix.R`
: Matrix specifying the among trait covariance for p traits. Can also be a single value (1) for a single trait

Value

matrix.TraitData Matrix of simulated trait data. Species are rows, traits are columns

Examples

```
#####################
# load dependencies #
#####################
library(geiger); library(phytools); library(TraitTrainR)

#########################
# get example phylogeny #
#########################
handle.PrimatePhylogeny <- read.tree(text = "((((((((human: 6, chimp:6): 1, gorilla: 7): 7, orangutan: 14): 11,

#############################
# Simulation Model Settings #
#############################
list.SimulationModelSettings <- list() # define an empty model list

##########################
# SET SIMULATION MODEL 1 #
##########################
numeric.NumberTrainingReps <- 10 # same number of replicates for all models in list.SimulationModelSettings
matrix.STACK_lrate_nodes <- matrix(replicate(n = numeric.NumberTrainingReps, sample(size = 1, x = 1:16, replac
matrix.STACK_lrate_rates <- matrix(replicate(n = numeric.NumberTrainingReps , runif(n = 1, min = 0, max = 100))
list.Rmatrix <- list(); for (i in 1:numeric.NumberTrainingReps){list.Rmatrix[[i]] <- matrix(c(1, 0, 0, 0, 1, 0,
#list.Rmatrix <- list(); for (i in 1:numeric.NumberTrainingReps){list.Rmatrix[[i]] <- 1} # single trait

#############################
# append to simulation list #
#############################
list.SimulationModelSettings[[1]] <- list(string.SimulationModel = "depth",
                            vector.AncestralState = runif(n = numeric.NumberTrainingReps, min = 0, max = 100),
                            matrix.STACK_lrate_nodes = matrix.STACK_lrate_nodes, matrix.STACK_lrate_rates =
                            vector.Depth = runif(n = numeric.NumberTrainingReps, min = 0, max = 10),
                                          list.Rmatrix = list.Rmatrix)

############################################################
# simulate under depth model with STACKED 10 (BM + lrates) #
############################################################
Simulate.TraitTrain.depth_STACKED_10(handle.Phylogeny = handle.PrimatePhylogeny,
                         numeric.AncestralState = list.SimulationModelSettings[[1]]$vector.AncestralState[1
                         numeric.depth = list.SimulationModelSettings[[1]]$vector.Depth[1],
                         vector.STACK_lrate_nodes = list.SimulationModelSettings[[1]]$matrix.STACK_lrate_noc
                         vector.STACK_lrate_rates = list.SimulationModelSettings[[1]]$matrix.STACK_lrate_ra
                          matrix.R = list.SimulationModelSettings[[1]]$list.Rmatrix[[1]])
```

`Simulate.TraitTrain.depth_STACKED_11`

Simulate.TraitTrain.depth_STACKED_11: Function to simulate trait data under the STACK (lrates + shifted) depth model

---

Description

This function returns a Matrix of simulated trait data. Species are rows, traits are columns in matrix.TraitData

Usage

```
Simulate.TraitTrain.depth_STACKED_11(
  numeric.NumberOfSpecies,
  handle.Phylogeny,
  numeric.depth,
  numeric.AncestralState,
  vector.STACK_lrate_nodes,
  vector.STACK_lrate_rates,
  vector.STACK_AncShiftNode,
  vector.STACK_AncShiftValue,
  matrix.R
)
```

Arguments

`numeric.NumberOfSpecies`
: Numeric counting the number of species

`handle.Phylogeny`
: Phylogeny used to simulate training data under depth model

`numeric.AncestralState`
: Numerical value of the z0 (ancestral state) parameter of the depth model

`vector.STACK_lrate_nodes`
: Vector that includes all nodes for a STACKED lrates model (on top of depth)

`vector.STACK_lrate_rates`
: Vector that includes all rate values for each node in vector.STACK_lrate_nodes for a STACKED lrates model (on top of depth)

`vector.STACK_AncShiftNode`
: Vector that includes all nodes for a STACKED AncShift model (on top of depth)

`vector.STACK_AncShiftValue`
: Vector that includes all shift values for each node in vector.STACK_AncShiftNode for a STACKED AncShift model (on top of depth)

`matrix.R`
: Matrix specifying the among trait covariance for p traits. Can also be a single value (1) for a single trait

Value

matrix.TraitData Matrix of simulated trait data. Species are rows, traits are columns

Examples

```
#####################
# load dependencies #
#####################
library(geiger); library(phytools); library(TraitTrainR)

#########################
# get example phylogeny #
```

```
##########################
handle.PrimatePhylogeny <- read.tree(text = "((((((((human: 6, chimp:6): 1, gorilla: 7): 7, orangutan: 14): 11,

##############################
# Simulation Model Settings #
##############################
list.SimulationModelSettings <- list() # define an empty model list

##########################
# SET SIMULATION MODEL 1 #
##########################
numeric.NumberTrainingReps <- 10 # same number of replicates for all models in list.SimulationModelSettings
matrix.STACK_lrate_nodes <- matrix(replicate(n = numeric.NumberTrainingReps, sample(size = 1, x = 1:16, replac
matrix.STACK_lrate_rates <- matrix(replicate(n = numeric.NumberTrainingReps , runif(n = 1, min = 0, max = 100))
matrix.STACK_AncShiftNode <- replicate(n = numeric.NumberTrainingReps, sample(size = 2, x = 1:16, replace = F))
matrix.STACK_AncShiftValue <- replicate(n = numeric.NumberTrainingReps , rnorm(n = 2, mean = 10000))
list.Rmatrix <- list(); for (i in 1:numeric.NumberTrainingReps){list.Rmatrix[[i]] <- matrix(c(1, 0, 0, 0, 1, 0,
#list.Rmatrix <- list(); for (i in 1:numeric.NumberTrainingReps){list.Rmatrix[[i]] <- 1} # single trait

##############################
# append to simulation list #
##############################
list.SimulationModelSettings[[1]] <- list(string.SimulationModel = "depth",
                            vector.AncestralState = runif(n = numeric.NumberTrainingReps, min = 0, max = 100),
                                    matrix.STACK_lrate_nodes = matrix.STACK_lrate_nodes,
                                    matrix.STACK_lrate_rates = matrix.STACK_lrate_rates,
                                  matrix.STACK_AncShiftNode = matrix.STACK_AncShiftNode,
                                matrix.STACK_AncShiftValue = matrix.STACK_AncShiftValue,
                            vector.Depth = runif(n = numeric.NumberTrainingReps, min = 0, max = 10),
                                          list.Rmatrix = list.Rmatrix)

######################################################################
# simulate under depth model with STACKED 11 (BM + lrates + AncShift) #
######################################################################
Simulate.TraitTrain.depth_STACKED_11(numeric.NumberOfSpecies = length(handle.PrimatePhylogeny$tip.label),
                                     handle.Phylogeny = handle.PrimatePhylogeny,
                         numeric.AncestralState = list.SimulationModelSettings[[1]]$vector.AncestralState[1]
                         numeric.depth = list.SimulationModelSettings[[1]]$vector.Depth[1],
                         vector.STACK_lrate_nodes = list.SimulationModelSettings[[1]]$matrix.STACK_lrate_noc
                         vector.STACK_lrate_rates = list.SimulationModelSettings[[1]]$matrix.STACK_lrate_rat
                         vector.STACK_AncShiftNode = list.SimulationModelSettings[[1]]$matrix.STACK_AncShift
                         vector.STACK_AncShiftValue = list.SimulationModelSettings[[1]]$matrix.STACK_AncShif
                          matrix.R = list.SimulationModelSettings[[1]]$list.Rmatrix[[1]])
```

`Simulate.TraitTrain.EB_00`

Simulate.TraitTrain.EB_00: Function to simulate trait data under the EB model

---

Description

This function returns a Matrix of simulated trait data. Species are rows, traits are columns in matrix.TraitData

Usage

```
Simulate.TraitTrain.EB_00(
  handle.Phylogeny,
  numeric.Sig2,
  numeric.A,
  numeric.AncestralState,
  matrix.R
)
```

Arguments

`handle.Phylogeny`
: Phylogeny used to simulate training data under EB model

`numeric.Sig2`
: Numeric Value of the sigsg (evolutionary rate) parmater of the EB model

`numeric.AncestralState`
: Numerical value of the z0 (ancestral state) parameter of the EB model

`matrix.R`
: Matrix specifying the among trait covariance for p traits. Can also be a single value (1) for a single trait

Value

matrix.TraitData Matrix of simulated trait data. Species are rows, traits are columns

Examples

```
#####################
# load dependencies #
#####################
library(geiger); library(phytools); library(TraitTrainR)

#########################
# get example phylogeny #
#########################
handle.PrimatePhylogeny <- read.tree(text = "((((((((human: 6, chimp:6): 1, gorilla: 7): 7, orangutan: 14): 11,

#############################
# Simulation Model Settings #
#############################
list.SimulationModelSettings <- list() # define an empty model list

##########################
# SET SIMULATION MODEL 1 #
##########################
numeric.NumberTrainingReps <- 10 # same number of replicates for all models in list.SimulationModelSettings
list.Rmatrix <- list(); for (i in 1:numeric.NumberTrainingReps){list.Rmatrix[[i]] <- matrix(c(1, 0, 0, 0, 1, 0,
#list.Rmatrix <- list(); for (i in 1:numeric.NumberTrainingReps){list.Rmatrix[[i]] <- 1} # single trait

#############################
# append to simulation list #
#############################
list.SimulationModelSettings[[1]] <- list(string.SimulationModel = "EB",
                             vector.Sig2 = rexp(n = numeric.NumberTrainingReps, rate = 1),
                            vector.AncestralState = runif(n = numeric.NumberTrainingReps, min = 0, max = 100),
                            vector.A = runif(n = numeric.NumberTrainingReps, min = log(10^-5)/310  , max = -0.0
```

```
                                                list.Rmatrix = list.Rmatrix)

###########################
# simulate under EB model #
###########################
Simulate.TraitTrain.EB_00(handle.Phylogeny = handle.PrimatePhylogeny,
                      numeric.Sig2 = list.SimulationModelSettings[[1]]$vector.Sig2[1],
                numeric.AncestralState = list.SimulationModelSettings[[1]]$vector.AncestralState[1],
                        numeric.A = list.SimulationModelSettings[[1]]$vector.A[1],
                       matrix.R = list.SimulationModelSettings[[1]]$list.Rmatrix[[1]])
```

Simulate.TraitTrain.EB_STACKED_01

Simulate.TraitTrain.EB_STACKED_01: Function to simulate trait data under the STACK (+AncShift) EB model

---

Description

This function returns a Matrix of simulated trait data. Species are rows, traits are columns in matrix.TraitData

Usage

```
Simulate.TraitTrain.EB_STACKED_01(
  numeric.NumberOfSpecies,
  handle.Phylogeny,
  numeric.Sig2,
  numeric.A,
  numeric.AncestralState,
  vector.STACK_AncShiftNode,
  vector.STACK_AncShiftValue,
  matrix.R
)
```

Arguments

`numeric.NumberOfSpecies`
: Numeric counting the number of species

`handle.Phylogeny`
: Phylogeny used to simulate training data under EB model

`numeric.Sig2`
: Numeric Value of the sigsg (evolutionary rate) parmater of the EB model

`numeric.AncestralState`
: Numerical value of the z0 (ancestral state) parameter of the EB model

`vector.STACK_AncShiftNode`
: Vector that includes all nodes for a STACKED AncShift model (on top of EB)

`vector.STACK_AncShiftValue`
: Vector that includes all shift values for each node in vector.STACK_AncShiftNode for a STACKED AncShift model (on top of EB)

`matrix.R`
: Matrix specifying the among trait covariance for p traits. Can also be a single value (1) for a single trait

Value

matrix.TraitData Matrix of simulated trait data. Species are rows, traits are columns

Examples

```
#####################
# load dependencies #
#####################
library(geiger); library(phytools); library(TraitTrainR)

#########################
# get example phylogeny #
#########################
handle.PrimatePhylogeny <- read.tree(text = "((((((((human: 6, chimp:6): 1, gorilla: 7): 7, orangutan: 14): 11,

#############################
# Simulation Model Settings #
#############################
list.SimulationModelSettings <- list() # define an empty model list

##########################
# SET SIMULATION MODEL 1 #
##########################
numeric.NumberTrainingReps <- 10 # same number of replicates for all models in list.SimulationModelSettings
matrix.STACK_AncShiftNode <- replicate(n = numeric.NumberTrainingReps, sample(size = 2, x = 1:16, replace = F))
matrix.STACK_AncShiftValue <- replicate(n = numeric.NumberTrainingReps , rnorm(n = 2, mean = 10000))
list.Rmatrix <- list(); for (i in 1:numeric.NumberTrainingReps){list.Rmatrix[[i]] <- matrix(c(1, 0, 0, 0, 1, 0,
#list.Rmatrix <- list(); for (i in 1:numeric.NumberTrainingReps){list.Rmatrix[[i]] <- 1} # single trait

#############################
# append to simulation list #
#############################
list.SimulationModelSettings[[1]] <- list(string.SimulationModel = "EB",
                             vector.Sig2 = rexp(n = numeric.NumberTrainingReps, rate = 1),
                            vector.AncestralState = runif(n = numeric.NumberTrainingReps, min = 0, max = 100),
                                  matrix.STACK_AncShiftNode = matrix.STACK_AncShiftNode,
                                matrix.STACK_AncShiftValue = matrix.STACK_AncShiftValue,
                            vector.A = runif(n = numeric.NumberTrainingReps, min = log(10^-5)/310  , max = -0.0
                                          list.Rmatrix = list.Rmatrix)

###########################################################
# simulate under EB model with STACKED 01 (EB + AncShift) #
###########################################################
Simulate.TraitTrain.EB_STACKED_01(numeric.NumberOfSpecies = length(handle.PrimatePhylogeny$tip.label),
                                  handle.Phylogeny = handle.PrimatePhylogeny,
                         numeric.Sig2 = list.SimulationModelSettings[[1]]$vector.Sig2[1],
                       numeric.AncestralState = list.SimulationModelSettings[[1]]$vector.AncestralState[1],
                       vector.STACK_AncShiftNode = list.SimulationModelSettings[[1]]$matrix.STACK_AncShiftNo
                       vector.STACK_AncShiftValue = list.SimulationModelSettings[[1]]$matrix.STACK_AncShiftV
                              numeric.A = list.SimulationModelSettings[[1]]$vector.A[1],
                          matrix.R = list.SimulationModelSettings[[1]]$list.Rmatrix[[1]])
```

`Simulate.TraitTrain.EB_STACKED_10`

Simulate.TraitTrain.EB_STACKED_10: Function to simulate trait data under the STACK (+lrates) EB model

Description

This function returns a Matrix of simulated trait data. Species are rows, traits are columns in matrix.TraitData

Usage

```
Simulate.TraitTrain.EB_STACKED_10(
  handle.Phylogeny,
  numeric.Sig2,
  numeric.A,
  numeric.AncestralState,
  vector.STACK_lrate_nodes,
  vector.STACK_lrate_rates,
  matrix.R
)
```

Arguments

`handle.Phylogeny`
: Phylogeny used to simulate training data under EB model

`numeric.Sig2`
: Numeric Value of the sigsg (evolutionary rate) parmater of the EB model

`numeric.AncestralState`
: Numerical value of the z0 (ancestral state) parameter of the EB model

`vector.STACK_lrate_nodes`
: Vector that includes all nodes for a STACKED lrates model (on top of EB)

`vector.STACK_lrate_rates`
: Vector that includes all rate values for each node in vector.STACK_lrate_nodes for a STACKED lrates model (on top of EB)

`matrix.R`
: Matrix specifying the among trait covariance for p traits. Can also be a single value (1) for a single trait

Value

matrix.TraitData Matrix of simulated trait data. Species are rows, traits are columns

Examples

```
#####################
# load dependencies #
#####################
library(geiger); library(phytools); library(TraitTrainR)

#########################
# get example phylogeny #
#########################
```

```
handle.PrimatePhylogeny <- read.tree(text = "((((((((human: 6, chimp:6): 1, gorilla: 7): 7, orangutan: 14): 11,

##############################
# Simulation Model Settings #
##############################
list.SimulationModelSettings <- list() # define an empty model list

###########################
# SET SIMULATION MODEL 1 #
###########################
numeric.NumberTrainingReps <- 10 # same number of replicates for all models in list.SimulationModelSettings
matrix.STACK_lrate_nodes <- matrix(replicate(n = numeric.NumberTrainingReps, sample(size = 1, x = 1:16, replac
matrix.STACK_lrate_rates <- matrix(replicate(n = numeric.NumberTrainingReps , runif(n = 1, min = 0, max = 100))
list.Rmatrix <- list(); for (i in 1:numeric.NumberTrainingReps){list.Rmatrix[[i]] <- matrix(c(1, 0, 0, 0, 1, 0,
#list.Rmatrix <- list(); for (i in 1:numeric.NumberTrainingReps){list.Rmatrix[[i]] <- 1} # single trait

##############################
# append to simulation list #
##############################
list.SimulationModelSettings[[1]] <- list(string.SimulationModel = "EB",
                             vector.Sig2 = rexp(n = numeric.NumberTrainingReps, rate = 1),
                            vector.AncestralState = runif(n = numeric.NumberTrainingReps, min = 0, max = 100),
                                    matrix.STACK_lrate_nodes = matrix.STACK_lrate_nodes,
                                    matrix.STACK_lrate_rates = matrix.STACK_lrate_rates,
                            vector.A = runif(n = numeric.NumberTrainingReps, min = log(10^-5)/310  , max = -0.0
                                          list.Rmatrix = list.Rmatrix)

#########################################################
# simulate under EB model with STACKED 10 (EB + lrates) #
#########################################################
Simulate.TraitTrain.EB_STACKED_10(handle.Phylogeny = handle.PrimatePhylogeny,
                          numeric.Sig2 = list.SimulationModelSettings[[1]]$vector.Sig2[1],
                        numeric.AncestralState = list.SimulationModelSettings[[1]]$vector.AncestralState[1],
                        vector.STACK_lrate_nodes = list.SimulationModelSettings[[1]]$matrix.STACK_lrate_nodes
                        vector.STACK_lrate_rates = list.SimulationModelSettings[[1]]$matrix.STACK_lrate_rates
                               numeric.A = list.SimulationModelSettings[[1]]$vector.A[1],
                           matrix.R = list.SimulationModelSettings[[1]]$list.Rmatrix[[1]])
```

`Simulate.TraitTrain.EB_STACKED_11`
Simulate.TraitTrain.EB_STACKED_11: Function to simulate trait data under the STACK (lrates + shifted) EB model

---

Description

This function returns a Matrix of simulated trait data. Species are rows, traits are columns in matrix.TraitData

Usage

```
Simulate.TraitTrain.EB_STACKED_11(
  numeric.NumberOfSpecies,
  handle.Phylogeny,
  numeric.Sig2,
```

```
  numeric.A,
  numeric.AncestralState,
  vector.STACK_lrate_nodes,
  vector.STACK_lrate_rates,
  vector.STACK_AncShiftNode,
  vector.STACK_AncShiftValue,
  matrix.R
)
```

Arguments

`numeric.NumberOfSpecies`
: Numeric counting the number of species

`handle.Phylogeny`
: Phylogeny used to simulate training data under EB model

`numeric.Sig2`
: Numeric Value of the sigsg (evolutionary rate) parmater of the EB model

`numeric.AncestralState`
: Numerical value of the z0 (ancestral state) parameter of the EB model

`vector.STACK_lrate_nodes`
: Vector that includes all nodes for a STACKED lrates model (on top of EB)

`vector.STACK_lrate_rates`
: Vector that includes all rate values for each node in vector.STACK_lrate_nodes for a STACKED lrates model (on top of EB)

`vector.STACK_AncShiftNode`
: Vector that includes all nodes for a STACKED AncShift model (on top of EB)

`vector.STACK_AncShiftValue`
: Vector that includes all shift values for each node in vector.STACK_AncShiftNode for a STACKED AncShift model (on top of EB)

`matrix.R`
: Matrix specifying the among trait covariance for p traits. Can also be a single value (1) for a single trait

Value

matrix.TraitData Matrix of simulated trait data. Species are rows, traits are columns

Examples

```
#####################
# load dependencies #
#####################
library(geiger); library(phytools); library(TraitTrainR)

#########################
# get example phylogeny #
#########################
handle.PrimatePhylogeny <- read.tree(text = "((((((((human: 6, chimp:6): 1, gorilla: 7): 7, orangutan: 14): 11,

#############################
# Simulation Model Settings #
#############################
list.SimulationModelSettings <- list() # define an empty model list

##########################
```

```
# SET SIMULATION MODEL 1 #
##########################
numeric.NumberTrainingReps <- 10 # same number of replicates for all models in list.SimulationModelSettings
matrix.STACK_lrate_nodes <- matrix(replicate(n = numeric.NumberTrainingReps, sample(size = 1, x = 1:16, replac
matrix.STACK_lrate_rates <- matrix(replicate(n = numeric.NumberTrainingReps , runif(n = 1, min = 0, max = 100))
matrix.STACK_AncShiftNode <- replicate(n = numeric.NumberTrainingReps, sample(size = 2, x = 1:16, replace = F))
matrix.STACK_AncShiftValue <- replicate(n = numeric.NumberTrainingReps , rnorm(n = 2, mean = 10000))
list.Rmatrix <- list(); for (i in 1:numeric.NumberTrainingReps){list.Rmatrix[[i]] <- matrix(c(1, 0, 0, 0, 1, 0,
#list.Rmatrix <- list(); for (i in 1:numeric.NumberTrainingReps){list.Rmatrix[[i]] <- 1} # single trait

#############################
# append to simulation list #
#############################
list.SimulationModelSettings[[1]] <- list(string.SimulationModel = "EB",
                              vector.Sig2 = rexp(n = numeric.NumberTrainingReps, rate = 1),
                             vector.AncestralState = runif(n = numeric.NumberTrainingReps, min = 0, max = 100),
                                     matrix.STACK_lrate_nodes = matrix.STACK_lrate_nodes,
                                     matrix.STACK_lrate_rates = matrix.STACK_lrate_rates,
                                   matrix.STACK_AncShiftNode = matrix.STACK_AncShiftNode,
                                 matrix.STACK_AncShiftValue = matrix.STACK_AncShiftValue,
                             vector.A = runif(n = numeric.NumberTrainingReps, min = log(10^-5)/310  , max = -0.0
                                           list.Rmatrix = list.Rmatrix)

#####################################################################
# simulate under EB model with STACKED 11 (EB + lrates + AncShift) #
#####################################################################
Simulate.TraitTrain.EB_STACKED_11(numeric.NumberOfSpecies = length(handle.PrimatePhylogeny$tip.label),
                                  handle.Phylogeny = handle.PrimatePhylogeny,
                        numeric.Sig2 = list.SimulationModelSettings[[1]]$vector.Sig2[1],
                      numeric.AncestralState = list.SimulationModelSettings[[1]]$vector.AncestralState[1],
                      vector.STACK_lrate_nodes = list.SimulationModelSettings[[1]]$matrix.STACK_lrate_nodes
                      vector.STACK_lrate_rates = list.SimulationModelSettings[[1]]$matrix.STACK_lrate_rates
                      vector.STACK_AncShiftNode = list.SimulationModelSettings[[1]]$matrix.STACK_AncShiftNo
                      vector.STACK_AncShiftValue = list.SimulationModelSettings[[1]]$matrix.STACK_AncShiftV
                             numeric.A = list.SimulationModelSettings[[1]]$vector.A[1],
                         matrix.R = list.SimulationModelSettings[[1]]$list.Rmatrix[[1]])
```

Simulate.TraitTrain.kappa_00

Simulate.TraitTrain.kappa_00: Function to simulate trait data under the kappa model

---

Description

This function returns a Matrix of simulated trait data. Species are rows, traits are columns in matrix.TraitData

Usage

```
Simulate.TraitTrain.kappa_00(
  handle.Phylogeny,
  numeric.Sig2,
  numeric.kappa,
  numeric.AncestralState,
```

```
  matrix.R
)
```

Arguments

handle.Phylogeny
: Phylogeny used to simulate training data under kappa model

numeric.Sig2
: Numeric Value of the sigsg (evolutionary rate) parmater of the kappa model

numeric.kappa
: Numeric value of the kappa parameter of the kappa model

numeric.AncestralState
: Numerical value of the z0 (ancestral state) parameter of the kappa model

matrix.R
: Matrix specifying the among trait covariance for p traits. Can also be a single value (1) for a single trait

numeric.NumberOfSpecies
: Numeric counting the number of species

Value

matrix.TraitData Matrix of simulated trait data. Species are rows, traits are columns

Examples

```
#####################
# load dependencies #
#####################
library(geiger); library(phytools); library(TraitTrainR)

#########################
# get example phylogeny #
#########################
handle.PrimatePhylogeny <- read.tree(text = "((((((((human: 6, chimp:6): 1, gorilla: 7): 7, orangutan: 14): 11,

#############################
# Simulation Model Settings #
#############################
list.SimulationModelSettings <- list() # define an empty model list

##########################
# SET SIMULATION MODEL 1 #
##########################
numeric.NumberTrainingReps <- 10 # same number of replicates for all models in list.SimulationModelSettings
list.Rmatrix <- list(); for (i in 1:numeric.NumberTrainingReps){list.Rmatrix[[i]] <- matrix(c(1, 0, 0, 0, 1, 0,
#list.Rmatrix <- list(); for (i in 1:numeric.NumberTrainingReps){list.Rmatrix[[i]] <- 1} # single trait

#############################
# append to simulation list #
#############################
list.SimulationModelSettings[[1]] <- list(string.SimulationModel = "kappa",
                             vector.Sig2 = rexp(n = numeric.NumberTrainingReps, rate = 1),
                            vector.AncestralState = runif(n = numeric.NumberTrainingReps, min = 0, max = 100),
                            vector.kappa = runif(n = numeric.NumberTrainingReps, min = exp(-500), max = 1),
                                          list.Rmatrix = list.Rmatrix)

#########################################
## simulate under kappa model with kappa #
```

```
##########################################
Simulate.TraitTrain.kappa_00(handle.Phylogeny = handle.PrimatePhylogeny,
                        numeric.Sig2 = list.SimulationModelSettings[[1]]$vector.Sig2[1],
                   numeric.AncestralState = list.SimulationModelSettings[[1]]$vector.AncestralState[1],
                      numeric.kappa = list.SimulationModelSettings[[1]]$vector.kappa[1],
                         matrix.R = list.SimulationModelSettings[[1]]$list.Rmatrix[[1]])
```

Simulate.TraitTrain.kappa_STACKED_01

Simulate.TraitTrain.kappa_STACKED_01: Function to simulate trait data under the STACK (+ AncShift) kappa model

---

Description

This function returns a Matrix of simulated trait data. Species are rows, traits are columns in matrix.TraitData

Usage

```
Simulate.TraitTrain.kappa_STACKED_01(
  numeric.NumberOfSpecies,
  handle.Phylogeny,
  numeric.Sig2,
  numeric.kappa,
  numeric.AncestralState,
  vector.STACK_AncShiftNode,
  vector.STACK_AncShiftValue,
  matrix.R
)
```

Arguments

`numeric.NumberOfSpecies`
: Numeric counting the number of species

`handle.Phylogeny`
: Phylogeny used to simulate training data under kappa model

`numeric.Sig2`
: Numeric Value of the sigsg (evolutionary rate) parmater of the kappa model

`numeric.kappa`
: Numerical value of the kappa parameter of the kappa model

`numeric.AncestralState`
: Numerical value of the z0 (ancestral state) parameter of the kappa model

`vector.STACK_AncShiftNode`
: Vector that includes all nodes for a STACKED AncShift model (on top of kappa)

`vector.STACK_AncShiftValue`
: Vector that includes all shift values for each node in vector.STACK_AncShiftNode for a STACKED AncShift model (on top of kappa)

`matrix.R`
: Matrix specifying the among trait covariance for p traits. Can also be a single value (1) for a single trait

Value

matrix.TraitData Matrix of simulated trait data. Species are rows, traits are columns

Examples

```
#####################
# load dependencies #
#####################
library(geiger); library(phytools); library(TraitTrainR)

#########################
# get example phylogeny #
#########################
handle.PrimatePhylogeny <- read.tree(text = "((((((((human: 6, chimp:6): 1, gorilla: 7): 7, orangutan: 14): 11,

#############################
# Simulation Model Settings #
#############################
list.SimulationModelSettings <- list() # define an empty model list

##########################
# SET SIMULATION MODEL 1 #
##########################
numeric.NumberTrainingReps <- 10 # same number of replicates for all models in list.SimulationModelSettings
matrix.STACK_AncShiftNode <- replicate(n = numeric.NumberTrainingReps, sample(size = 2, x = 1:16, replace = F))
matrix.STACK_AncShiftValue <- replicate(n = numeric.NumberTrainingReps , rnorm(n = 2, mean = 10000))
list.Rmatrix <- list(); for (i in 1:numeric.NumberTrainingReps){list.Rmatrix[[i]] <- matrix(c(1, 0, 0, 0, 1, 0,
#list.Rmatrix <- list(); for (i in 1:numeric.NumberTrainingReps){list.Rmatrix[[i]] <- 1} # single trait

#############################
# append to simulation list #
#############################
list.SimulationModelSettings[[1]] <- list(string.SimulationModel = "kappa",
                             vector.Sig2 = rexp(n = numeric.NumberTrainingReps, rate = 1),
                            vector.AncestralState = runif(n = numeric.NumberTrainingReps, min = 0, max = 100),
                                  matrix.STACK_AncShiftNode = matrix.STACK_AncShiftNode,
                                matrix.STACK_AncShiftValue = matrix.STACK_AncShiftValue,
                            vector.kappa = runif(n = numeric.NumberTrainingReps, min = exp(-500), max = 1),
                                          list.Rmatrix = list.Rmatrix)

#################################################################
# simulate under kappa model with STACKED 01 (kappa + AncShift) #
#################################################################
Simulate.TraitTrain.kappa_STACKED_01(numeric.NumberOfSpecies = length(handle.PrimatePhylogeny$tip.label),
                                     handle.Phylogeny = handle.PrimatePhylogeny,
                         numeric.Sig2 = list.SimulationModelSettings[[1]]$vector.Sig2[1],
                         numeric.AncestralState = list.SimulationModelSettings[[1]]$vector.AncestralState[1]
                         vector.STACK_AncShiftNode = list.SimulationModelSettings[[1]]$matrix.STACK_AncShift
                         vector.STACK_AncShiftValue = list.SimulationModelSettings[[1]]$matrix.STACK_AncShi
                         numeric.kappa = list.SimulationModelSettings[[1]]$vector.kappa[1],
                          matrix.R = list.SimulationModelSettings[[1]]$list.Rmatrix[[1]])
```

`Simulate.TraitTrain.kappa_STACKED_10`

Simulate.TraitTrain.kappa_STACKED_10: Function to simulate trait data under the STACK (lrates) kappa model

---

Description

This function returns a Matrix of simulated trait data. Species are rows, traits are columns in matrix.TraitData

Usage

```
Simulate.TraitTrain.kappa_STACKED_10(
  handle.Phylogeny,
  numeric.Sig2,
  numeric.kappa,
  numeric.AncestralState,
  vector.STACK_lrate_nodes,
  vector.STACK_lrate_rates,
  matrix.R
)
```

Arguments

`handle.Phylogeny`	Phylogeny used to simulate training data under kappa model
`numeric.Sig2`	Numeric Value of the sigsg (evolutionary rate) parmater of the kappa model
`numeric.kappa`	Numerical value of the kappa parameter of the kappa model
`numeric.AncestralState`	Numerical value of the z0 (ancestral state) parameter of the kappa model
`vector.STACK_lrate_nodes`	Vector that includes all nodes for a STACKED lrates model (on top of kappa)
`vector.STACK_lrate_rates`	Vector that includes all rate values for each node in vector.STACK_lrate_nodes for a STACKED lrates model (on top of kappa)
`matrix.R`	Matrix specifying the among trait covariance for p traits. Can also be a single value (1) for a single trait
`numeric.NumberOfSpecies`	Numeric counting the number of species

Value

matrix.TraitData Matrix of simulated trait data. Species are rows, traits are columns

Examples

```
#####################
# load dependencies #
#####################
library(geiger); library(phytools); library(TraitTrainR)

#########################
# get example phylogeny #
#########################
handle.PrimatePhylogeny <- read.tree(text = "((((((((human: 6, chimp:6): 1, gorilla: 7): 7, orangutan: 14): 11,

#############################
# Simulation Model Settings #
```

```
##############################
list.SimulationModelSettings <- list() # define an empty model list

##########################
# SET SIMULATION MODEL 1 #
##########################
numeric.NumberTrainingReps <- 10 # same number of replicates for all models in list.SimulationModelSettings
matrix.STACK_lrate_nodes <- matrix(replicate(n = numeric.NumberTrainingReps, sample(size = 1, x = 1:16, replac
matrix.STACK_lrate_rates <- matrix(replicate(n = numeric.NumberTrainingReps , runif(n = 1, min = 0, max = 100))
list.Rmatrix <- list(); for (i in 1:numeric.NumberTrainingReps){list.Rmatrix[[i]] <- matrix(c(1, 0, 0, 0, 1, 0,
#list.Rmatrix <- list(); for (i in 1:numeric.NumberTrainingReps){list.Rmatrix[[i]] <- 1} # single trait

##############################
# append to simulation list #
##############################
list.SimulationModelSettings[[1]] <- list(string.SimulationModel = "kappa",
                             vector.Sig2 = rexp(n = numeric.NumberTrainingReps, rate = 1),
                            vector.AncestralState = runif(n = numeric.NumberTrainingReps, min = 0, max = 100),
                                    matrix.STACK_lrate_nodes = matrix.STACK_lrate_nodes,
                                    matrix.STACK_lrate_rates = matrix.STACK_lrate_rates,
                            vector.kappa = runif(n = numeric.NumberTrainingReps, min = exp(-500), max = 1),
                                          list.Rmatrix = list.Rmatrix)

##################################################################
## simulate under kappa model with STACKED 10 (kappa + lrates ) #
##################################################################
Simulate.TraitTrain.kappa_STACKED_10(handle.Phylogeny = handle.PrimatePhylogeny,
                         numeric.Sig2 = list.SimulationModelSettings[[1]]$vector.Sig2[1],
                         numeric.AncestralState = list.SimulationModelSettings[[1]]$vector.AncestralState[1]
                         vector.STACK_lrate_nodes = list.SimulationModelSettings[[1]]$matrix.STACK_lrate_noc
                         vector.STACK_lrate_rates = list.SimulationModelSettings[[1]]$matrix.STACK_lrate_rat
                         numeric.kappa = list.SimulationModelSettings[[1]]$vector.kappa[1],
                          matrix.R = list.SimulationModelSettings[[1]]$list.Rmatrix[[1]])
```

Simulate.TraitTrain.kappa_STACKED_11

Simulate.TraitTrain.kappa_STACKED_11: Function to simulate trait data under the STACK (lrates + shifted) kappa model

---

Description

This function returns a Matrix of simulated trait data. Species are rows, traits are columns in matrix.TraitData

Usage

```
Simulate.TraitTrain.kappa_STACKED_11(
  numeric.NumberOfSpecies,
  handle.Phylogeny,
  numeric.Sig2,
  numeric.kappa,
  numeric.AncestralState,
  vector.STACK_lrate_nodes,
  vector.STACK_lrate_rates,
```

```
  vector.STACK_AncShiftNode,
  vector.STACK_AncShiftValue,
  matrix.R
)
```

Arguments

`numeric.NumberOfSpecies`
: Numeric counting the number of species

`handle.Phylogeny`
: Phylogeny used to simulate training data under kappa model

`numeric.Sig2`
: Numeric Value of the sigsg (evolutionary rate) parmater of the kappa model

`numeric.kappa`
: Numerical value of the kappa parameter of the kappa model

`numeric.AncestralState`
: Numerical value of the z0 (ancestral state) parameter of the kappa model

`vector.STACK_lrate_nodes`
: Vector that includes all nodes for a STACKED lrates model (on top of kappa)

`vector.STACK_lrate_rates`
: Vector that includes all rate values for each node in vector.STACK_lrate_nodes for a STACKED lrates model (on top of kappa)

`vector.STACK_AncShiftNode`
: Vector that includes all nodes for a STACKED AncShift model (on top of kappa)

`vector.STACK_AncShiftValue`
: Vector that includes all shift values for each node in vector.STACK_AncShiftNode for a STACKED AncShift model (on top of kappa)

`matrix.R`
: Matrix specifying the among trait covariance for p traits. Can also be a single value (1) for a single trait

Value

matrix.TraitData Matrix of simulated trait data. Species are rows, traits are columns

Examples

```
#####################
# load dependencies #
#####################
library(geiger); library(phytools); library(TraitTrainR)

#########################
# get example phylogeny #
#########################
handle.PrimatePhylogeny <- read.tree(text = "((((((((human: 6, chimp:6): 1, gorilla: 7): 7, orangutan: 14): 11,

#############################
# Simulation Model Settings #
#############################
list.SimulationModelSettings <- list() # define an empty model list

##########################
# SET SIMULATION MODEL 1 #
##########################
numeric.NumberTrainingReps <- 10 # same number of replicates for all models in list.SimulationModelSettings
```

```
matrix.STACK_lrate_nodes <- matrix(replicate(n = numeric.NumberTrainingReps, sample(size = 1, x = 1:16, replac
matrix.STACK_lrate_rates <- matrix(replicate(n = numeric.NumberTrainingReps , runif(n = 1, min = 0, max = 100))
matrix.STACK_AncShiftNode <- replicate(n = numeric.NumberTrainingReps, sample(size = 2, x = 1:16, replace = F))
matrix.STACK_AncShiftValue <- replicate(n = numeric.NumberTrainingReps , rnorm(n = 2, mean = 10000))
list.Rmatrix <- list(); for (i in 1:numeric.NumberTrainingReps){list.Rmatrix[[i]] <- matrix(c(1, 0, 0, 0, 1, 0,
#list.Rmatrix <- list(); for (i in 1:numeric.NumberTrainingReps){list.Rmatrix[[i]] <- 1} # single trait

#############################
# append to simulation list #
#############################
list.SimulationModelSettings[[1]] <- list(string.SimulationModel = "kappa",
                              vector.Sig2 = rexp(n = numeric.NumberTrainingReps, rate = 1),
                             vector.AncestralState = runif(n = numeric.NumberTrainingReps, min = 0, max = 100),
                                     matrix.STACK_lrate_nodes = matrix.STACK_lrate_nodes,
                                     matrix.STACK_lrate_rates = matrix.STACK_lrate_rates,
                                   matrix.STACK_AncShiftNode = matrix.STACK_AncShiftNode,
                                 matrix.STACK_AncShiftValue = matrix.STACK_AncShiftValue,
                             vector.kappa = runif(n = numeric.NumberTrainingReps, min = exp(-500), max = 1),
                                           list.Rmatrix = list.Rmatrix)
############################################################################
## simulate under kappa model with STACKED 11 (kappa + lrates + AncShift) #
############################################################################
Simulate.TraitTrain.kappa_STACKED_11(numeric.NumberOfSpecies = length(handle.PrimatePhylogeny$tip.label),
                                     handle.Phylogeny = handle.PrimatePhylogeny,
                         numeric.Sig2 = list.SimulationModelSettings[[1]]$vector.Sig2[1],
                         numeric.AncestralState = list.SimulationModelSettings[[1]]$vector.AncestralState[1]
                         vector.STACK_lrate_nodes = list.SimulationModelSettings[[1]]$matrix.STACK_lrate_noc
                         vector.STACK_lrate_rates = list.SimulationModelSettings[[1]]$matrix.STACK_lrate_rat
                         vector.STACK_AncShiftNode = list.SimulationModelSettings[[1]]$matrix.STACK_AncShift
                         vector.STACK_AncShiftValue = list.SimulationModelSettings[[1]]$matrix.STACK_AncShif
                         numeric.kappa = list.SimulationModelSettings[[1]]$vector.kappa[1],
                          matrix.R = list.SimulationModelSettings[[1]]$list.Rmatrix[[1]])
```

Simulate.TraitTrain.lambda_00 *Simulate.TraitTrain.lambda_00: Function to simulate trait data under the lambda model*

Description

This function returns a Matrix of simulated trait data. Species are rows, traits are columns in matrix.TraitData

Usage

```
Simulate.TraitTrain.lambda_00(
  handle.Phylogeny,
  numeric.Sig2,
  numeric.lambda,
  numeric.AncestralState,
  matrix.R
)
```

Arguments

`handle.Phylogeny`
: Phylogeny used to simulate training data under lambda model

`numeric.Sig2`
: Numeric Value of the sigsg (evolutionary rate) parmater of the lambda model

`numeric.lambda`
: Numerical value of the lambda parameter of the lambda model

`numeric.AncestralState`
: Numerical value of the z0 (ancestral state) parameter of the lambda model

`matrix.R`
: Matrix specifying the among trait covariance for p traits. Can also be a single value (1) for a single trait

`numeric.NumberOfSpecies`
: Numeric counting the number of species

Value

matrix.TraitData Matrix of simulated trait data. Species are rows, traits are columns

Examples

```
#####################
# load dependencies #
#####################
library(geiger); library(phytools); library(TraitTrainR)

#########################
# get example phylogeny #
#########################
handle.PrimatePhylogeny <- read.tree(text = "((((((((human: 6, chimp:6): 1, gorilla: 7): 7, orangutan: 14): 11,

#############################
# Simulation Model Settings #
#############################
list.SimulationModelSettings <- list() # define an empty model list

##########################
# SET SIMULATION MODEL 1 #
##########################
numeric.NumberTrainingReps <- 10 # same number of replicates for all models in list.SimulationModelSettings
list.Rmatrix <- list(); for (i in 1:numeric.NumberTrainingReps){list.Rmatrix[[i]] <- matrix(c(1, 0, 0, 0, 1, 0,
#list.Rmatrix <- list(); for (i in 1:numeric.NumberTrainingReps){list.Rmatrix[[i]] <- 1} # single trait

#############################
# append to simulation list #
#############################
list.SimulationModelSettings[[1]] <- list(string.SimulationModel = "lambda",
                             vector.Sig2 = rexp(n = numeric.NumberTrainingReps, rate = 1),
                            vector.AncestralState = runif(n = numeric.NumberTrainingReps, min = 0, max = 100),
                            vector.lambda = runif(n = numeric.NumberTrainingReps, min = exp(-500), max = 1),
                                          list.Rmatrix = list.Rmatrix)

###############################
# simulate under lambda model #
###############################
Simulate.TraitTrain.lambda_00(handle.Phylogeny = handle.PrimatePhylogeny,
                        numeric.Sig2 = list.SimulationModelSettings[[1]]$vector.Sig2[1],
```

```
                        numeric.AncestralState = list.SimulationModelSettings[[1]]$vector.AncestralState[1],
                        numeric.lambda = list.SimulationModelSettings[[1]]$vector.lambda[1],
                            matrix.R = list.SimulationModelSettings[[1]]$list.Rmatrix[[1]])
```

Simulate.TraitTrain.lambda_STACKED_01
Simulate.TraitTrain.lambda_STACKED_01: Function to simulate trait data under the STACK (+ AncShift) lambda model

---

Description

This function returns a Matrix of simulated trait data. Species are rows, traits are columns in matrix.TraitData

Usage

```
Simulate.TraitTrain.lambda_STACKED_01(
  numeric.NumberOfSpecies,
  handle.Phylogeny,
  numeric.Sig2,
  numeric.lambda,
  numeric.AncestralState,
  vector.STACK_AncShiftNode,
  vector.STACK_AncShiftValue,
  matrix.R
)
```

Arguments

`numeric.NumberOfSpecies`
: Numeric counting the number of species

`handle.Phylogeny`
: Phylogeny used to simulate training data under lambda model

`numeric.Sig2`
: Numeric Value of the sigsg (evolutionary rate) parmater of the lambda model

`numeric.lambda`
: Numerical value of the lambda parameter of the lambda model

`numeric.AncestralState`
: Numerical value of the z0 (ancestral state) parameter of the lambda model

`vector.STACK_AncShiftNode`
: Vector that includes all nodes for a STACKED AncShift model (on top of lambda)

`vector.STACK_AncShiftValue`
: Vector that includes all shift values for each node in vector.STACK_AncShiftNode for a STACKED AncShift model (on top of lambda)

`matrix.R`
: Matrix specifying the among trait covariance for p traits. Can also be a single value (1) for a single trait

Value

matrix.TraitData Matrix of simulated trait data. Species are rows, traits are columns

Examples

```
#####################
# load dependencies #
#####################
library(geiger); library(phytools); library(TraitTrainR)

#########################
# get example phylogeny #
#########################
handle.PrimatePhylogeny <- read.tree(text = "((((((((human: 6, chimp:6): 1, gorilla: 7): 7, orangutan: 14): 11,

#############################
# Simulation Model Settings #
#############################
list.SimulationModelSettings <- list() # define an empty model list

##########################
# SET SIMULATION MODEL 1 #
##########################
numeric.NumberTrainingReps <- 10 # same number of replicates for all models in list.SimulationModelSettings
matrix.STACK_AncShiftNode <- replicate(n = numeric.NumberTrainingReps, sample(size = 2, x = 1:16, replace = F))
matrix.STACK_AncShiftValue <- replicate(n = numeric.NumberTrainingReps , rnorm(n = 2, mean = 10000))
list.Rmatrix <- list(); for (i in 1:numeric.NumberTrainingReps){list.Rmatrix[[i]] <- matrix(c(1, 0, 0, 0, 1, 0,
#list.Rmatrix <- list(); for (i in 1:numeric.NumberTrainingReps){list.Rmatrix[[i]] <- 1} # single trait

#############################
# append to simulation list #
#############################
list.SimulationModelSettings[[1]] <- list(string.SimulationModel = "lambda",
                             vector.Sig2 = rexp(n = numeric.NumberTrainingReps, rate = 1),
                            vector.AncestralState = runif(n = numeric.NumberTrainingReps, min = 0, max = 100),
                                  matrix.STACK_AncShiftNode = matrix.STACK_AncShiftNode,
                                matrix.STACK_AncShiftValue = matrix.STACK_AncShiftValue,
                            vector.lambda = runif(n = numeric.NumberTrainingReps, min = exp(-500), max = 1),
                                          list.Rmatrix = list.Rmatrix)

###################################################################
# simulate under lambda model with STACKED 01 (lambda + AncShift) #
###################################################################
Simulate.TraitTrain.lambda_STACKED_01(numeric.NumberOfSpecies = length(handle.PrimatePhylogeny$tip.label),
                                      handle.Phylogeny = handle.PrimatePhylogeny,
                          numeric.Sig2 = list.SimulationModelSettings[[1]]$vector.Sig2[1],
                          numeric.AncestralState = list.SimulationModelSettings[[1]]$vector.AncestralState[1
                          vector.STACK_AncShiftNode = list.SimulationModelSettings[[1]]$matrix.STACK_AncShif
                          vector.STACK_AncShiftValue = list.SimulationModelSettings[[1]]$matrix.STACK_AncShi
                          numeric.lambda = list.SimulationModelSettings[[1]]$vector.lambda[1],
                          matrix.R = list.SimulationModelSettings[[1]]$list.Rmatrix[[1]])
```

`Simulate.TraitTrain.lambda_STACKED_10`
Simulate.TraitTrain.lambda_STACKED_10: Function to simulate trait data under the STACK (+lrates) lambda model

---

Description

This function returns a Matrix of simulated trait data. Species are rows, traits are columns in matrix.TraitData

Usage

```
Simulate.TraitTrain.lambda_STACKED_10(
  handle.Phylogeny,
  numeric.Sig2,
  numeric.lambda,
  numeric.AncestralState,
  vector.STACK_lrate_nodes,
  vector.STACK_lrate_rates,
  matrix.R
)
```

Arguments

`handle.Phylogeny`
: Phylogeny used to simulate training data under lambda model

`numeric.Sig2`
: Numeric Value of the sigsg (evolutionary rate) parmater of the lambda model

`numeric.lambda`
: Numerical value of the lambda parameter of the lambda model

`numeric.AncestralState`
: Numerical value of the z0 (ancestral state) parameter of the lambda model

`vector.STACK_lrate_nodes`
: Vector that includes all nodes for a STACKED lrates model (on top of lambda)

`vector.STACK_lrate_rates`
: Vector that includes all rate values for each node in vector.STACK_lrate_nodes for a STACKED lrates model (on top of lambda)

`matrix.R`
: Matrix specifying the among trait covariance for p traits. Can also be a single value (1) for a single trait

`numeric.NumberOfSpecies`
: Numeric counting the number of species

Value

matrix.TraitData Matrix of simulated trait data. Species are rows, traits are columns

Examples

```
#####################
# load dependencies #
#####################
library(geiger); library(phytools); library(TraitTrainR)

#########################
# get example phylogeny #
#########################
handle.PrimatePhylogeny <- read.tree(text = "((((((((human: 6, chimp:6): 1, gorilla: 7): 7, orangutan: 14): 11,

#############################
# Simulation Model Settings #
```

```
##############################
list.SimulationModelSettings <- list() # define an empty model list

##########################
# SET SIMULATION MODEL 1 #
##########################
numeric.NumberTrainingReps <- 10 # same number of replicates for all models in list.SimulationModelSettings
matrix.STACK_lrate_nodes <- matrix(replicate(n = numeric.NumberTrainingReps, sample(size = 1, x = 1:16, replac
matrix.STACK_lrate_rates <- matrix(replicate(n = numeric.NumberTrainingReps , runif(n = 1, min = 0, max = 100))
list.Rmatrix <- list(); for (i in 1:numeric.NumberTrainingReps){list.Rmatrix[[i]] <- matrix(c(1, 0, 0, 0, 1, 0,
#list.Rmatrix <- list(); for (i in 1:numeric.NumberTrainingReps){list.Rmatrix[[i]] <- 1} # single trait

##############################
# append to simulation list #
##############################
list.SimulationModelSettings[[1]] <- list(string.SimulationModel = "lambda",
                             vector.Sig2 = rexp(n = numeric.NumberTrainingReps, rate = 1),
                            vector.AncestralState = runif(n = numeric.NumberTrainingReps, min = 0, max = 100),
                                    matrix.STACK_lrate_nodes = matrix.STACK_lrate_nodes,
                                    matrix.STACK_lrate_rates = matrix.STACK_lrate_rates,
                            vector.lambda = runif(n = numeric.NumberTrainingReps, min = exp(-500), max = 1),
                                          list.Rmatrix = list.Rmatrix)

##################################################################
# simulate under lambda model with STACKED 10 (lambda + lrates ) #
##################################################################
Simulate.TraitTrain.lambda_STACKED_10(handle.Phylogeny = handle.PrimatePhylogeny,
                          numeric.Sig2 = list.SimulationModelSettings[[1]]$vector.Sig2[1],
                          numeric.AncestralState = list.SimulationModelSettings[[1]]$vector.AncestralState[1
                          vector.STACK_lrate_nodes = list.SimulationModelSettings[[1]]$matrix.STACK_lrate_nc
                          vector.STACK_lrate_rates = list.SimulationModelSettings[[1]]$matrix.STACK_lrate_ra
                          numeric.lambda = list.SimulationModelSettings[[1]]$vector.lambda[1],
                          matrix.R = list.SimulationModelSettings[[1]]$list.Rmatrix[[1]])
```

`Simulate.TraitTrain.lambda_STACKED_11`
Simulate.TraitTrain.lambda_STACKED_11: Function to simulate trait data under the STACK (lrates + shifted) lambda model

---

Description

This function returns a Matrix of simulated trait data. Species are rows, traits are columns in matrix.TraitData

Usage

```
Simulate.TraitTrain.lambda_STACKED_11(
  numeric.NumberOfSpecies,
  handle.Phylogeny,
  numeric.Sig2,
  numeric.lambda,
  numeric.AncestralState,
  vector.STACK_lrate_nodes,
```

```
  vector.STACK_lrate_rates,
  vector.STACK_AncShiftNode,
  vector.STACK_AncShiftValue,
  matrix.R
)
```

Arguments

numeric.NumberOfSpecies
: Numeric counting the number of species

handle.Phylogeny
: Phylogeny used to simulate training data under lambda model

numeric.Sig2
: Numeric Value of the sigsg (evolutionary rate) parmater of the lambda model

numeric.lambda
: Numerical value of the lambda parameter of the lambda model

numeric.AncestralState
: Numerical value of the z0 (ancestral state) parameter of the lambda model

vector.STACK_lrate_nodes
: Vector that includes all nodes for a STACKED lrates model (on top of lambda)

vector.STACK_lrate_rates
: Vector that includes all rate values for each node in vector.STACK_lrate_nodes for a STACKED lrates model (on top of lambda)

vector.STACK_AncShiftNode
: Vector that includes all nodes for a STACKED AncShift model (on top of lambda)

vector.STACK_AncShiftValue
: Vector that includes all shift values for each node in vector.STACK_AncShiftNode for a STACKED AncShift model (on top of lambda)

matrix.R
: Matrix specifying the among trait covariance for p traits. Can also be a single value (1) for a single trait

Value

matrix.TraitData Matrix of simulated trait data. Species are rows, traits are columns

Examples

```
#####################
# load dependencies #
#####################
library(geiger); library(phytools); library(TraitTrainR)

#########################
# get example phylogeny #
#########################
handle.PrimatePhylogeny <- read.tree(text = "((((((((human: 6, chimp:6): 1, gorilla: 7): 7, orangutan: 14): 11,

#############################
# Simulation Model Settings #
#############################
list.SimulationModelSettings <- list() # define an empty model list

##########################
# SET SIMULATION MODEL 1 #
##########################
```

```
numeric.NumberTrainingReps <- 10 # same number of replicates for all models in list.SimulationModelSettings
matrix.STACK_lrate_nodes <- matrix(replicate(n = numeric.NumberTrainingReps, sample(size = 1, x = 1:16, replac
matrix.STACK_lrate_rates <- matrix(replicate(n = numeric.NumberTrainingReps , runif(n = 1, min = 0, max = 100))
matrix.STACK_AncShiftNode <- replicate(n = numeric.NumberTrainingReps, sample(size = 2, x = 1:16, replace = F))
matrix.STACK_AncShiftValue <- replicate(n = numeric.NumberTrainingReps , rnorm(n = 2, mean = 10000))
list.Rmatrix <- list(); for (i in 1:numeric.NumberTrainingReps){list.Rmatrix[[i]] <- matrix(c(1, 0, 0, 0, 1, 0,
#list.Rmatrix <- list(); for (i in 1:numeric.NumberTrainingReps){list.Rmatrix[[i]] <- 1} # single trait

##############################
# append to simulation list #
##############################
list.SimulationModelSettings[[1]] <- list(string.SimulationModel = "lambda",
                              vector.Sig2 = rexp(n = numeric.NumberTrainingReps, rate = 1),
                             vector.AncestralState = runif(n = numeric.NumberTrainingReps, min = 0, max = 100),
                                    matrix.STACK_lrate_nodes = matrix.STACK_lrate_nodes,
                                    matrix.STACK_lrate_rates = matrix.STACK_lrate_rates,
                                  matrix.STACK_AncShiftNode = matrix.STACK_AncShiftNode,
                                matrix.STACK_AncShiftValue = matrix.STACK_AncShiftValue,
                             vector.lambda = runif(n = numeric.NumberTrainingReps, min = exp(-500), max = 1),
                                          list.Rmatrix = list.Rmatrix)

###########################################################################
# simulate under lambda model with STACKED 11 (lambda + lrates + AncShift) #
###########################################################################
Simulate.TraitTrain.lambda_STACKED_11(numeric.NumberOfSpecies = length(handle.PrimatePhylogeny$tip.label),
                                     handle.Phylogeny = handle.PrimatePhylogeny,
                         numeric.Sig2 = list.SimulationModelSettings[[1]]$vector.Sig2[1],
                         numeric.AncestralState = list.SimulationModelSettings[[1]]$vector.AncestralState[1
                         vector.STACK_lrate_nodes = list.SimulationModelSettings[[1]]$matrix.STACK_lrate_nc
                         vector.STACK_lrate_rates = list.SimulationModelSettings[[1]]$matrix.STACK_lrate_ra
                         vector.STACK_AncShiftNode = list.SimulationModelSettings[[1]]$matrix.STACK_AncShif
                         vector.STACK_AncShiftValue = list.SimulationModelSettings[[1]]$matrix.STACK_AncShi
                         numeric.lambda = list.SimulationModelSettings[[1]]$vector.lambda[1],
                         matrix.R = list.SimulationModelSettings[[1]]$list.Rmatrix[[1]])
```

Simulate.TraitTrain.lrate_00 *Simulate.TraitTrain.lrate_00: Function to simulate trait data under the lrate model*

Description

This function returns a Matrix of simulated trait data. Species are rows, traits are columns in matrix.TraitData

Usage

```
Simulate.TraitTrain.lrate_00(
  handle.Phylogeny,
  numeric.Sig2,
  numeric.AncestralState,
  vector.lrate_node,
  vector.lrate_rate,
  matrix.R
)
```

Arguments

`handle.Phylogeny`
: Phylogeny used to simulate training data under lrate model

`numeric.Sig2`
: Numeric Value of the sigsg (evolutionary rate) parmater of the lrate model

`numeric.AncestralState`
: Numerical value of the z0 (ancestral state) parameter of the lrate model

`vector.lrate_node`
: Vector that includes all nodes (can be multiple) for the lrate model

`vector.lrate_rate`
: Vector that includes all rates (can be multiple) for the lrate model

`matrix.R`
: Matrix specifying the among trait covariance for p traits. Can also be a single value (1) for a single trait

`numeric.NumberOfSpecies`
: Numeric counting the number of species

Value

matrix.TraitData Matrix of simulated trait data. Species are rows, traits are columns

Examples

```
#####################
# load dependencies #
#####################
library(geiger); library(phytools); library(TraitTrainR)

#########################
# get example phylogeny #
#########################
handle.PrimatePhylogeny <- read.tree(text = "((((((((human: 6, chimp:6): 1, gorilla: 7): 7, orangutan: 14): 11,

#############################
# Simulation Model Settings #
#############################
list.SimulationModelSettings <- list() # define an empty model list

##########################
# SET SIMULATION MODEL 1 #
##########################
numeric.NumberTrainingReps <- 10 # same number of replicates for all models in list.SimulationModelSettings
matrix.lrate_node <- matrix(replicate(n = numeric.NumberTrainingReps, sample(size = 1, x = 1:16, replace = F)),
matrix.lrate_rate <- matrix(replicate(n = numeric.NumberTrainingReps , runif(n = 1, min = 0, max = 100)), nrow =
list.Rmatrix <- list(); for (i in 1:numeric.NumberTrainingReps){list.Rmatrix[[i]] <- matrix(c(1, 0, 0, 0, 1, 0,
#list.Rmatrix <- list(); for (i in 1:numeric.NumberTrainingReps){list.Rmatrix[[i]] <- 1} # single trait

#############################
# append to simulation list #
#############################
list.SimulationModelSettings[[1]] <- list(string.SimulationModel = "lrate",
                              vector.Sig2 = rexp(n = numeric.NumberTrainingReps, rate = 1),
                             vector.AncestralState = runif(n = numeric.NumberTrainingReps, min = 0, max = 100),
                                            matrix.lrate_node = matrix.lrate_node,
                                            matrix.lrate_rate = matrix.lrate_rate,
                                            list.Rmatrix = list.Rmatrix)
```

```
###############################
# simulate under lrate model #
###############################
Simulate.TraitTrain.lrate_00(handle.Phylogeny = handle.PrimatePhylogeny,
                         numeric.Sig2 = list.SimulationModelSettings[[1]]$vector.Sig2[1],
                    numeric.AncestralState = list.SimulationModelSettings[[1]]$vector.AncestralState[1],
                    vector.lrate_node = list.SimulationModelSettings[[1]]$matrix.lrate_node[,1],
                    vector.lrate_rate = list.SimulationModelSettings[[1]]$matrix.lrate_rate[,1],
                          matrix.R = list.SimulationModelSettings[[1]]$list.Rmatrix[[1]])
```

Simulate.TraitTrain.lrate_STACKED_01

Simulate.TraitTrain.lrate_STACKED_11: Function to simulate trait data under the STACK (+AncShifted)) lrate model

---

Description

This function returns a Matrix of simulated trait data. Species are rows, traits are columns in matrix.TraitData

Usage

```
Simulate.TraitTrain.lrate_STACKED_01(
  numeric.NumberOfSpecies,
  handle.Phylogeny,
  numeric.Sig2,
  numeric.AncestralState,
  vector.lrate_node,
  vector.lrate_rate,
  vector.STACK_AncShiftNode,
  vector.STACK_AncShiftValue,
  matrix.R
)
```

Arguments

`numeric.NumberOfSpecies`	Numeric counting the number of species
`handle.Phylogeny`	Phylogeny used to simulate training data under lrate model
`numeric.Sig2`	Numeric Value of the sigsg (evolutionary rate) parmater of the lrate model
`numeric.AncestralState`	Numerical value of the z0 (ancestral state) parameter of the lrate model
`vector.lrate_node`	Vector that includes all nodes (can be multiple) for the lrate model
`vector.lrate_rate`	Vector that includes all rates (can be multiple) for the lrate model
`vector.STACK_AncShiftNode`	Vector that includes all nodes for a STACKED AncShift model (on top of lrate)

vector.STACK_AncShiftValue
: Vector that includes all shift values for each node in vector.STACK_AncShiftNode for a STACKED AncShift model (on top of lrate)

matrix.R
: Matrix specifying the among trait covariance for p traits. Can also be a single value (1) for a single trait

Value

matrix.TraitData Matrix of simulated trait data. Species are rows, traits are columns

Examples

```
#####################
# load dependencies #
#####################
library(geiger); library(phytools); library(TraitTrainR)

#########################
# get example phylogeny #
#########################
handle.PrimatePhylogeny <- read.tree(text = "((((((((human: 6, chimp:6): 1, gorilla: 7): 7, orangutan: 14): 11,

#############################
# Simulation Model Settings #
#############################
list.SimulationModelSettings <- list() # define an empty model list

##########################
# SET SIMULATION MODEL 1 #
##########################
numeric.NumberTrainingReps <- 10 # same number of replicates for all models in list.SimulationModelSettings
matrix.lrate_node <- matrix(replicate(n = numeric.NumberTrainingReps, sample(size = 1, x = 1:16, replace = F)),
matrix.lrate_rate <- matrix(replicate(n = numeric.NumberTrainingReps , runif(n = 1, min = 0, max = 100)), nrow =
matrix.STACK_AncShiftNode <- replicate(n = numeric.NumberTrainingReps, sample(size = 2, x = 1:16, replace = F))
matrix.STACK_AncShiftValue <- replicate(n = numeric.NumberTrainingReps , rnorm(n = 2, mean = 10000))
list.Rmatrix <- list(); for (i in 1:numeric.NumberTrainingReps){list.Rmatrix[[i]] <- matrix(c(1, 0, 0, 0, 1, 0,
#list.Rmatrix <- list(); for (i in 1:numeric.NumberTrainingReps){list.Rmatrix[[i]] <- 1} # single trait

#############################
# append to simulation list #
#############################
list.SimulationModelSettings[[1]] <- list(string.SimulationModel = "lrate",
                             vector.Sig2 = rexp(n = numeric.NumberTrainingReps, rate = 1),
                            vector.AncestralState = runif(n = numeric.NumberTrainingReps, min = 0, max = 100),
                                  matrix.STACK_AncShiftNode = matrix.STACK_AncShiftNode,
                              matrix.STACK_AncShiftValue = matrix.STACK_AncShiftValue,
                                        matrix.lrate_node = matrix.lrate_node,
                                        matrix.lrate_rate = matrix.lrate_rate,
                                        list.Rmatrix = list.Rmatrix)

#################################################################
# simulate under lrate model with STACKED 01 (lrate + AncShift) #
#################################################################
Simulate.TraitTrain.lrate_STACKED_01(numeric.NumberOfSpecies = length(handle.PrimatePhylogeny$tip.label),
                                     handle.Phylogeny = handle.PrimatePhylogeny,
                        numeric.Sig2 = list.SimulationModelSettings[[1]]$vector.Sig2[1],
                        numeric.AncestralState = list.SimulationModelSettings[[1]]$vector.AncestralState[1]
```

```
vector.lrate_node = list.SimulationModelSettings[[1]]$matrix.lrate_node[,1],
vector.lrate_rate = list.SimulationModelSettings[[1]]$matrix.lrate_rate[,1],
vector.STACK_AncShiftNode = list.SimulationModelSettings[[1]]$matrix.STACK_AncShift
vector.STACK_AncShiftValue = list.SimulationModelSettings[[1]]$matrix.STACK_AncShi
 matrix.R = list.SimulationModelSettings[[1]]$list.Rmatrix[[1]])
```

`Simulate.TraitTrain.lrate_STACKED_10`
Simulate.TraitTrain.lrate_STACKED_11: Function to simulate trait data under the STACK (+lrates) lrate model

---

Description

This function returns a Matrix of simulated trait data. Species are rows, traits are columns in matrix.TraitData

Usage

```
Simulate.TraitTrain.lrate_STACKED_10(
  handle.Phylogeny,
  numeric.Sig2,
  numeric.AncestralState,
  vector.lrate_node,
  vector.lrate_rate,
  vector.STACK_lrate_nodes,
  vector.STACK_lrate_rates,
  matrix.R
)
```

Arguments

`handle.Phylogeny`
: Phylogeny used to simulate training data under lrate model

`numeric.Sig2`
: Numeric Value of the sigsg (evolutionary rate) parmater of the lrate model

`numeric.AncestralState`
: Numerical value of the z0 (ancestral state) parameter of the lrate model

`vector.lrate_node`
: Vector that includes all nodes (can be multiple) for the lrate model

`vector.lrate_rate`
: Vector that includes all rates (can be multiple) for the lrate model

`vector.STACK_lrate_nodes`
: Vector that includes all nodes for a STACKED lrates model (on top of lrate)

`vector.STACK_lrate_rates`
: Vector that includes all rate values for each node in vector.STACK_lrate_nodes for a STACKED lrates model (on top of lrate)

`matrix.R`
: Matrix specifying the among trait covariance for p traits. Can also be a single value (1) for a single trait

`numeric.NumberOfSpecies`
: Numeric counting the number of species

Value

matrix.TraitData Matrix of simulated trait data. Species are rows, traits are columns

Examples

```
######################
# load dependencies #
######################
library(geiger); library(phytools); library(TraitTrainR)

##########################
# get example phylogeny #
##########################
handle.PrimatePhylogeny <- read.tree(text = "((((((((human: 6, chimp:6): 1, gorilla: 7): 7, orangutan: 14): 11,

##############################
# Simulation Model Settings #
##############################
list.SimulationModelSettings <- list() # define an empty model list

##########################
# SET SIMULATION MODEL 1 #
##########################
numeric.NumberTrainingReps <- 10 # same number of replicates for all models in list.SimulationModelSettings
matrix.lrate_node <- matrix(replicate(n = numeric.NumberTrainingReps, sample(size = 1, x = 1:16, replace = F)),
matrix.lrate_rate <- matrix(replicate(n = numeric.NumberTrainingReps , runif(n = 1, min = 0, max = 100)), nrow =
matrix.STACK_lrate_nodes <- matrix(replicate(n = numeric.NumberTrainingReps, sample(size = 1, x = 1:16, replac
matrix.STACK_lrate_rates <- matrix(replicate(n = numeric.NumberTrainingReps , runif(n = 1, min = 0, max = 100))
list.Rmatrix <- list(); for (i in 1:numeric.NumberTrainingReps){list.Rmatrix[[i]] <- matrix(c(1, 0, 0, 0, 1, 0,
#list.Rmatrix <- list(); for (i in 1:numeric.NumberTrainingReps){list.Rmatrix[[i]] <- 1} # single trait

##############################
# append to simulation list #
##############################
list.SimulationModelSettings[[1]] <- list(string.SimulationModel = "lrate",
                             vector.Sig2 = rexp(n = numeric.NumberTrainingReps, rate = 1),
                            vector.AncestralState = runif(n = numeric.NumberTrainingReps, min = 0, max = 100),
                                    matrix.STACK_lrate_nodes = matrix.STACK_lrate_nodes,
                                    matrix.STACK_lrate_rates = matrix.STACK_lrate_rates,
                                          matrix.lrate_node = matrix.lrate_node,
                                          matrix.lrate_rate = matrix.lrate_rate,
                                          list.Rmatrix = list.Rmatrix)

################################################################
# simulate under lrate model with STACKED 10 (lrate + lrates) #
################################################################
Simulate.TraitTrain.lrate_STACKED_10(handle.Phylogeny = handle.PrimatePhylogeny,
                         numeric.Sig2 = list.SimulationModelSettings[[1]]$vector.Sig2[1],
                         numeric.AncestralState = list.SimulationModelSettings[[1]]$vector.AncestralState[1]
                         vector.lrate_node = list.SimulationModelSettings[[1]]$matrix.lrate_node[,1],
                         vector.lrate_rate = list.SimulationModelSettings[[1]]$matrix.lrate_rate[,1],
                         vector.STACK_lrate_nodes = list.SimulationModelSettings[[1]]$matrix.STACK_lrate_noc
                         vector.STACK_lrate_rates = list.SimulationModelSettings[[1]]$matrix.STACK_lrate_rat
                          matrix.R = list.SimulationModelSettings[[1]]$list.Rmatrix[[1]])
```

`Simulate.TraitTrain.lrate_STACKED_11`
Simulate.TraitTrain.lrate_STACKED_11: Function to simulate trait data under the STACK (lrates + shifted) lrate model

Description

This function returns a Matrix of simulated trait data. Species are rows, traits are columns in matrix.TraitData

Usage

```
Simulate.TraitTrain.lrate_STACKED_11(
  numeric.NumberOfSpecies,
  handle.Phylogeny,
  numeric.Sig2,
  numeric.AncestralState,
  vector.lrate_node,
  vector.lrate_rate,
  vector.STACK_lrate_nodes,
  vector.STACK_lrate_rates,
  vector.STACK_AncShiftNode,
  vector.STACK_AncShiftValue,
  matrix.R
)
```

Arguments

`numeric.NumberOfSpecies`
: Numeric counting the number of species

`handle.Phylogeny`
: Phylogeny used to simulate training data under lrate model

`numeric.Sig2`
: Numeric Value of the sigsg (evolutionary rate) parmater of the lrate model

`numeric.AncestralState`
: Numerical value of the z0 (ancestral state) parameter of the lrate model

`vector.lrate_node`
: Vector that includes all nodes (can be multiple) for the lrate model

`vector.lrate_rate`
: Vector that includes all rates (can be multiple) for the lrate model

`vector.STACK_lrate_nodes`
: Vector that includes all nodes for a STACKED lrates model (on top of lrate)

`vector.STACK_lrate_rates`
: Vector that includes all rate values for each node in vector.STACK_lrate_nodes for a STACKED lrates model (on top of lrate)

`vector.STACK_AncShiftNode`
: Vector that includes all nodes for a STACKED AncShift model (on top of lrate)

`vector.STACK_AncShiftValue`
: Vector that includes all shift values for each node in vector.STACK_AncShiftNode for a STACKED AncShift model (on top of lrate)

`matrix.R`
: Matrix specifying the among trait covariance for p traits. Can also be a single value (1) for a single trait

Value

matrix.TraitData Matrix of simulated trait data. Species are rows, traits are columns

Examples

```
#####################
# load dependencies #
#####################
library(geiger); library(phytools); library(TraitTrainR)

#########################
# get example phylogeny #
#########################
handle.PrimatePhylogeny <- read.tree(text = "((((((((human: 6, chimp:6): 1, gorilla: 7): 7, orangutan: 14): 11,

##############################
# Simulation Model Settings #
##############################
list.SimulationModelSettings <- list() # define an empty model list

##########################
# SET SIMULATION MODEL 1 #
##########################
numeric.NumberTrainingReps <- 10 # same number of replicates for all models in list.SimulationModelSettings
matrix.lrate_node <- matrix(replicate(n = numeric.NumberTrainingReps, sample(size = 1, x = 1:16, replace = F)),
matrix.lrate_rate <- matrix(replicate(n = numeric.NumberTrainingReps , runif(n = 1, min = 0, max = 100)), nrow =
matrix.STACK_lrate_nodes <- matrix(replicate(n = numeric.NumberTrainingReps, sample(size = 1, x = 1:16, replac
matrix.STACK_lrate_rates <- matrix(replicate(n = numeric.NumberTrainingReps , runif(n = 1, min = 0, max = 100))
matrix.STACK_AncShiftNode <- replicate(n = numeric.NumberTrainingReps, sample(size = 2, x = 1:16, replace = F))
matrix.STACK_AncShiftValue <- replicate(n = numeric.NumberTrainingReps , rnorm(n = 2, mean = 10000))
list.Rmatrix <- list(); for (i in 1:numeric.NumberTrainingReps){list.Rmatrix[[i]] <- matrix(c(1, 0, 0, 0, 1, 0,
#list.Rmatrix <- list(); for (i in 1:numeric.NumberTrainingReps){list.Rmatrix[[i]] <- 1} # single trait

#############################
# append to simulation list #
#############################
list.SimulationModelSettings[[1]] <- list(string.SimulationModel = "lrate",
                             vector.Sig2 = rexp(n = numeric.NumberTrainingReps, rate = 1),
                            vector.AncestralState = runif(n = numeric.NumberTrainingReps, min = 0, max = 100),
                                    matrix.STACK_lrate_nodes = matrix.STACK_lrate_nodes,
                                    matrix.STACK_lrate_rates = matrix.STACK_lrate_rates,
                                  matrix.STACK_AncShiftNode = matrix.STACK_AncShiftNode,
                                matrix.STACK_AncShiftValue = matrix.STACK_AncShiftValue,
                                          matrix.lrate_node = matrix.lrate_node,
                                          matrix.lrate_rate = matrix.lrate_rate,
                                          list.Rmatrix = list.Rmatrix)

##########################################################################
# simulate under lrate model with STACKED 11 (lrate + lrates + AncShift) #
#########################################################################
Simulate.TraitTrain.lrate_STACKED_11(numeric.NumberOfSpecies = length(handle.PrimatePhylogeny$tip.label),
                                     handle.Phylogeny = handle.PrimatePhylogeny,
                        numeric.Sig2 = list.SimulationModelSettings[[1]]$vector.Sig2[1],
                        numeric.AncestralState = list.SimulationModelSettings[[1]]$vector.AncestralState[1]
                        vector.lrate_node = list.SimulationModelSettings[[1]]$matrix.lrate_node[,1],
                        vector.lrate_rate = list.SimulationModelSettings[[1]]$matrix.lrate_rate[,1],
```

```
vector.STACK_lrate_nodes = list.SimulationModelSettings[[1]]$matrix.STACK_lrate_noc
vector.STACK_lrate_rates = list.SimulationModelSettings[[1]]$matrix.STACK_lrate_rat
vector.STACK_AncShiftNode = list.SimulationModelSettings[[1]]$matrix.STACK_AncShift
vector.STACK_AncShiftValue = list.SimulationModelSettings[[1]]$matrix.STACK_AncShi
 matrix.R = list.SimulationModelSettings[[1]]$list.Rmatrix[[1]])
```

`Simulate.TraitTrain.nrate_00`
Simulate.TraitTrain.nrate_00: Function to simulate trait data under the nrate model

---

Description

This function returns a Matrix of simulated trait data. Species are rows, traits are columns in matrix.TraitData

Usage

```
Simulate.TraitTrain.nrate_00(
  handle.Phylogeny,
  numeric.Sig2,
  numeric.AncestralState,
  vector.nrate_time,
  vector.nrate_rate,
  matrix.R
)
```

Arguments

`handle.Phylogeny`
: Phylogeny used to simulate training data under nrate model

`numeric.Sig2`
: Numeric Value of the sigsg (evolutionary rate) parmater of the nrate model

`numeric.AncestralState`
: Numerical value of the z0 (ancestral state) parameter of the nrate model

`vector.nrate_time`
: Vector that includes all times (can be multiple) for the nrate model

`vector.nrate_rate`
: Vector that includes all rates (can be multiple) for the nrate model

`matrix.R`
: Matrix specifying the among trait covariance for p traits. Can also be a single value (1) for a single trait

`numeric.NumberOfSpecies`
: Numeric counting the number of species

Value

matrix.TraitData Matrix of simulated trait data. Species are rows, traits are columns

Examples

```
#####################
# load dependencies #
#####################
library(geiger); library(phytools); library(TraitTrainR)

#########################
# get example phylogeny #
#########################
handle.PrimatePhylogeny <- read.tree(text = "((((((((human: 6, chimp:6): 1, gorilla: 7): 7, orangutan: 14): 11,

#############################
# Simulation Model Settings #
#############################
list.SimulationModelSettings <- list() # define an empty model list

##########################
# SET SIMULATION MODEL 1 #
##########################
numeric.NumberTrainingReps <- 10 # same number of replicates for all models in list.SimulationModelSettings
matrix.nrate_time <- matrix(replicate(n = numeric.NumberTrainingReps, runif(n = 1, min = 0, max = 1)), nrow = T)
matrix.nrate_rate <- matrix(replicate(n = numeric.NumberTrainingReps , runif(n = 1, min = 0, max = 100)), nrow =
list.Rmatrix <- list(); for (i in 1:numeric.NumberTrainingReps){list.Rmatrix[[i]] <- matrix(c(1, 0, 0, 0, 1, 0,
#list.Rmatrix <- list(); for (i in 1:numeric.NumberTrainingReps){list.Rmatrix[[i]] <- 1} # single trait

#############################
# append to simulation list #
#############################
list.SimulationModelSettings[[1]] <- list(string.SimulationModel = "nrate",
                             vector.Sig2 = rexp(n = numeric.NumberTrainingReps, rate = 1),
                            vector.AncestralState = runif(n = numeric.NumberTrainingReps, min = 0, max = 100),
                                          matrix.nrate_rate = matrix.nrate_rate,
                                          matrix.nrate_time = matrix.nrate_time,
                                          list.Rmatrix = list.Rmatrix)

##############################
# simulate under nrate model #
##############################
Simulate.TraitTrain.nrate_00(handle.Phylogeny = handle.PrimatePhylogeny,
                        numeric.Sig2 = list.SimulationModelSettings[[1]]$vector.Sig2[1],
                   numeric.AncestralState = list.SimulationModelSettings[[1]]$vector.AncestralState[1],
                   vector.nrate_time = list.SimulationModelSettings[[1]]$matrix.nrate_time[,1],
                   vector.nrate_rate = list.SimulationModelSettings[[1]]$matrix.nrate_rate[,1],
                         matrix.R = list.SimulationModelSettings[[1]]$list.Rmatrix[[1]])
```

`Simulate.TraitTrain.nrate_STACKED_01`

Simulate.TraitTrain.nrate_STACKED_01: Function to simulate trait data under the STACK (+AncShift) nrate model

---

Description

This function returns a Matrix of simulated trait data. Species are rows, traits are columns in matrix.TraitData

Usage

```
Simulate.TraitTrain.nrate_STACKED_01(
  numeric.NumberOfSpecies,
  handle.Phylogeny,
  numeric.Sig2,
  numeric.AncestralState,
  vector.nrate_time,
  vector.nrate_rate,
  vector.STACK_AncShiftNode,
  vector.STACK_AncShiftValue,
  matrix.R
)
```

Arguments

`numeric.NumberOfSpecies`
: Numeric counting the number of species

`handle.Phylogeny`
: Phylogeny used to simulate training data under nrate model

`numeric.Sig2`
: Numeric Value of the sigsg (evolutionary rate) parmater of the nrate model

`numeric.AncestralState`
: Numerical value of the z0 (ancestral state) parameter of the nrate model

`vector.nrate_time`
: Vector that includes all times (can be multiple) for the nrate model

`vector.nrate_rate`
: Vector that includes all rates (can be multiple) for the nrate model

`vector.STACK_AncShiftNode`
: Vector that includes all nodes for a STACKED AncShift model (on top of nrate)

`vector.STACK_AncShiftValue`
: Vector that includes all shift values for each node in vector.STACK_AncShiftNode for a STACKED AncShift model (on top of nrate)

`matrix.R`
: Matrix specifying the among trait covariance for p traits. Can also be a single value (1) for a single trait

Value

matrix.TraitData Matrix of simulated trait data. Species are rows, traits are columns

Examples

```
#####################
# load dependencies #
#####################
library(geiger); library(phytools); library(TraitTrainR)

#########################
# get example phylogeny #
#########################
handle.PrimatePhylogeny <- read.tree(text = "((((((((human: 6, chimp:6): 1, gorilla: 7): 7, orangutan: 14): 11,

#############################
# Simulation Model Settings #
```

```
##############################
list.SimulationModelSettings <- list() # define an empty model list

###########################
# SET SIMULATION MODEL 1 #
###########################
numeric.NumberTrainingReps <- 10 # same number of replicates for all models in list.SimulationModelSettings
matrix.nrate_time <- matrix(replicate(n = numeric.NumberTrainingReps, runif(n = 1, min = 0, max = 1)), nrow = T)
matrix.nrate_rate <- matrix(replicate(n = numeric.NumberTrainingReps , runif(n = 1, min = 0, max = 100)), nrow =
matrix.STACK_AncShiftNode <- replicate(n = numeric.NumberTrainingReps, sample(size = 2, x = 1:16, replace = F))
matrix.STACK_AncShiftValue <- replicate(n = numeric.NumberTrainingReps , rnorm(n = 2, mean = 10000))
list.Rmatrix <- list(); for (i in 1:numeric.NumberTrainingReps){list.Rmatrix[[i]] <- matrix(c(1, 0, 0, 0, 1, 0,
#list.Rmatrix <- list(); for (i in 1:numeric.NumberTrainingReps){list.Rmatrix[[i]] <- 1} # single trait

##############################
# append to simulation list #
##############################
list.SimulationModelSettings[[1]] <- list(string.SimulationModel = "nrate",
                             vector.Sig2 = rexp(n = numeric.NumberTrainingReps, rate = 1),
                            vector.AncestralState = runif(n = numeric.NumberTrainingReps, min = 0, max = 100),
                                  matrix.STACK_AncShiftNode = matrix.STACK_AncShiftNode,
                                matrix.STACK_AncShiftValue = matrix.STACK_AncShiftValue,
                                          matrix.nrate_rate = matrix.nrate_rate,
                                          matrix.nrate_time = matrix.nrate_time,
                                          list.Rmatrix = list.Rmatrix)

#################################################################
# simulate under nrate model with STACKED 01 (nrate + AncShift) #
#################################################################
Simulate.TraitTrain.nrate_STACKED_01(numeric.NumberOfSpecies = length(handle.PrimatePhylogeny$tip.label),
                                     handle.Phylogeny = handle.PrimatePhylogeny,
                        numeric.Sig2 = list.SimulationModelSettings[[1]]$vector.Sig2[1],
                        numeric.AncestralState = list.SimulationModelSettings[[1]]$vector.AncestralState[1]
                        vector.nrate_time = list.SimulationModelSettings[[1]]$matrix.nrate_time[,1],
                        vector.nrate_rate = list.SimulationModelSettings[[1]]$matrix.nrate_rate[,1],
                        vector.STACK_AncShiftNode = list.SimulationModelSettings[[1]]$matrix.STACK_AncShif
                        vector.STACK_AncShiftValue = list.SimulationModelSettings[[1]]$matrix.STACK_AncShi
                         matrix.R = list.SimulationModelSettings[[1]]$list.Rmatrix[[1]])
```

Simulate.TraitTrain.nrate_STACKED_10

Simulate.TraitTrain.nrate_STACKED_10: Function to simulate trait data under the STACK (+lrates) nrate model

---

Description

This function returns a Matrix of simulated trait data. Species are rows, traits are columns in matrix.TraitData

Usage

```
Simulate.TraitTrain.nrate_STACKED_10(
  handle.Phylogeny,
  numeric.Sig2,
```

```
  numeric.AncestralState,
  vector.nrate_time,
  vector.nrate_rate,
  vector.STACK_lrate_nodes,
  vector.STACK_lrate_rates,
  matrix.R
)
```

Arguments

`handle.Phylogeny`
: Phylogeny used to simulate training data under nrate model

`numeric.Sig2`
: Numeric Value of the sigsg (evolutionary rate) parmater of the nrate model

`numeric.AncestralState`
: Numerical value of the z0 (ancestral state) parameter of the nrate model

`vector.nrate_time`
: Vector that includes all times (can be multiple) for the nrate model

`vector.nrate_rate`
: Vector that includes all rates (can be multiple) for the nrate model

`vector.STACK_lrate_nodes`
: Vector that includes all nodes for a STACKED lrates model (on top of nrate)

`vector.STACK_lrate_rates`
: Vector that includes all rate values for each node in vector.STACK_lrate_nodes for a STACKED lrates model (on top of nrate)

`matrix.R`
: Matrix specifying the among trait covariance for p traits. Can also be a single value (1) for a single trait

`numeric.NumberOfSpecies`
: Numeric counting the number of species

Value

matrix.TraitData Matrix of simulated trait data. Species are rows, traits are columns

Examples

```
#####################
# load dependencies #
#####################
library(geiger); library(phytools); library(TraitTrainR)

#########################
# get example phylogeny #
#########################
handle.PrimatePhylogeny <- read.tree(text = "((((((((human: 6, chimp:6): 1, gorilla: 7): 7, orangutan: 14): 11,

#############################
# Simulation Model Settings #
#############################
list.SimulationModelSettings <- list() # define an empty model list

##########################
# SET SIMULATION MODEL 1 #
##########################
```

```
numeric.NumberTrainingReps <- 10 # same number of replicates for all models in list.SimulationModelSettings
matrix.nrate_time <- matrix(replicate(n = numeric.NumberTrainingReps, runif(n = 1, min = 0, max = 1)), nrow = T)
matrix.nrate_rate <- matrix(replicate(n = numeric.NumberTrainingReps , runif(n = 1, min = 0, max = 100)), nrow =
matrix.STACK_lrate_nodes <- matrix(replicate(n = numeric.NumberTrainingReps, sample(size = 1, x = 1:16, replac
matrix.STACK_lrate_rates <- matrix(replicate(n = numeric.NumberTrainingReps , runif(n = 1, min = 0, max = 100))
list.Rmatrix <- list(); for (i in 1:numeric.NumberTrainingReps){list.Rmatrix[[i]] <- matrix(c(1, 0, 0, 0, 1, 0,
#list.Rmatrix <- list(); for (i in 1:numeric.NumberTrainingReps){list.Rmatrix[[i]] <- 1} # single trait

##############################
# append to simulation list #
##############################
list.SimulationModelSettings[[1]] <- list(string.SimulationModel = "nrate",
                             vector.Sig2 = rexp(n = numeric.NumberTrainingReps, rate = 1),
                            vector.AncestralState = runif(n = numeric.NumberTrainingReps, min = 0, max = 100),
                                    matrix.STACK_lrate_nodes = matrix.STACK_lrate_nodes,
                                    matrix.STACK_lrate_rates = matrix.STACK_lrate_rates,
                                          matrix.nrate_rate = matrix.nrate_rate,
                                          matrix.nrate_time = matrix.nrate_time,
                                          list.Rmatrix = list.Rmatrix)

################################################################
# simulate under nrate model with STACKED 10 (nrate + lrates ) #
################################################################
Simulate.TraitTrain.nrate_STACKED_10(handle.Phylogeny = handle.PrimatePhylogeny,
                        numeric.Sig2 = list.SimulationModelSettings[[1]]$vector.Sig2[1],
                        numeric.AncestralState = list.SimulationModelSettings[[1]]$vector.AncestralState[1]
                        vector.nrate_time = list.SimulationModelSettings[[1]]$matrix.nrate_time[,1],
                        vector.nrate_rate = list.SimulationModelSettings[[1]]$matrix.nrate_rate[,1],
                        vector.STACK_lrate_nodes = list.SimulationModelSettings[[1]]$matrix.STACK_lrate_noc
                        vector.STACK_lrate_rates = list.SimulationModelSettings[[1]]$matrix.STACK_lrate_rat
                         matrix.R = list.SimulationModelSettings[[1]]$list.Rmatrix[[1]])
```

`Simulate.TraitTrain.nrate_STACKED_11`

Simulate.TraitTrain.nrate_STACKED_11: Function to simulate trait data under the STACK (lrates + shifted) nrate model

---

Description

This function returns a Matrix of simulated trait data. Species are rows, traits are columns in matrix.TraitData

Usage

```
Simulate.TraitTrain.nrate_STACKED_11(
  numeric.NumberOfSpecies,
  handle.Phylogeny,
  numeric.Sig2,
  numeric.AncestralState,
  vector.nrate_time,
  vector.nrate_rate,
  vector.STACK_lrate_nodes,
  vector.STACK_lrate_rates,
```

```
  vector.STACK_AncShiftNode,
  vector.STACK_AncShiftValue,
  matrix.R
)
```

Arguments

`numeric.NumberOfSpecies`
: Numeric counting the number of species

`handle.Phylogeny`
: Phylogeny used to simulate training data under nrate model

`numeric.Sig2`
: Numeric Value of the sigsg (evolutionary rate) parmater of the nrate model

`numeric.AncestralState`
: Numerical value of the z0 (ancestral state) parameter of the nrate model

`vector.nrate_time`
: Vector that includes all times (can be multiple) for the nrate model

`vector.nrate_rate`
: Vector that includes all rates (can be multiple) for the nrate model

`vector.STACK_lrate_nodes`
: Vector that includes all nodes for a STACKED lrates model (on top of nrate)

`vector.STACK_lrate_rates`
: Vector that includes all rate values for each node in vector.STACK_lrate_nodes for a STACKED lrates model (on top of nrate)

`vector.STACK_AncShiftNode`
: Vector that includes all nodes for a STACKED AncShift model (on top of nrate)

`vector.STACK_AncShiftValue`
: Vector that includes all shift values for each node in vector.STACK_AncShiftNode for a STACKED AncShift model (on top of nrate)

`matrix.R`
: Matrix specifying the among trait covariance for p traits. Can also be a single value (1) for a single trait

Value

matrix.TraitData Matrix of simulated trait data. Species are rows, traits are columns

Examples

```
#####################
# load dependencies #
#####################
library(geiger); library(phytools); library(TraitTrainR)

#########################
# get example phylogeny #
#########################
handle.PrimatePhylogeny <- read.tree(text = "((((((((human: 6, chimp:6): 1, gorilla: 7): 7, orangutan: 14): 11,

#############################
# Simulation Model Settings #
#############################
list.SimulationModelSettings <- list() # define an empty model list
```

```
##########################
# SET SIMULATION MODEL 1 #
##########################
numeric.NumberTrainingReps <- 10 # same number of replicates for all models in list.SimulationModelSettings
matrix.nrate_time <- matrix(replicate(n = numeric.NumberTrainingReps, runif(n = 1, min = 0, max = 1)), nrow = T)
matrix.nrate_rate <- matrix(replicate(n = numeric.NumberTrainingReps , runif(n = 1, min = 0, max = 100)), nrow =
matrix.STACK_lrate_nodes <- matrix(replicate(n = numeric.NumberTrainingReps, sample(size = 1, x = 1:16, replac
matrix.STACK_lrate_rates <- matrix(replicate(n = numeric.NumberTrainingReps , runif(n = 1, min = 0, max = 100))
matrix.STACK_AncShiftNode <- replicate(n = numeric.NumberTrainingReps, sample(size = 2, x = 1:16, replace = F))
matrix.STACK_AncShiftValue <- replicate(n = numeric.NumberTrainingReps , rnorm(n = 2, mean = 10000))
list.Rmatrix <- list(); for (i in 1:numeric.NumberTrainingReps){list.Rmatrix[[i]] <- matrix(c(1, 0, 0, 0, 1, 0,
#list.Rmatrix <- list(); for (i in 1:numeric.NumberTrainingReps){list.Rmatrix[[i]] <- 1} # single trait

#############################
# append to simulation list #
#############################
list.SimulationModelSettings[[1]] <- list(string.SimulationModel = "nrate",
                             vector.Sig2 = rexp(n = numeric.NumberTrainingReps, rate = 1),
                            vector.AncestralState = runif(n = numeric.NumberTrainingReps, min = 0, max = 100),
                                    matrix.STACK_lrate_nodes = matrix.STACK_lrate_nodes,
                                    matrix.STACK_lrate_rates = matrix.STACK_lrate_rates,
                                  matrix.STACK_AncShiftNode = matrix.STACK_AncShiftNode,
                                matrix.STACK_AncShiftValue = matrix.STACK_AncShiftValue,
                                          matrix.nrate_rate = matrix.nrate_rate,
                                          matrix.nrate_time = matrix.nrate_time,
                                          list.Rmatrix = list.Rmatrix)

########################################################################
# simulate under nrate model with STACKED 11 (nrate + lrates + AncShift) #
########################################################################
Simulate.TraitTrain.nrate_STACKED_11(numeric.NumberOfSpecies = length(handle.PrimatePhylogeny$tip.label),
                                         handle.Phylogeny = handle.PrimatePhylogeny,
                        numeric.Sig2 = list.SimulationModelSettings[[1]]$vector.Sig2[1],
                        numeric.AncestralState = list.SimulationModelSettings[[1]]$vector.AncestralState[1]
                        vector.nrate_time = list.SimulationModelSettings[[1]]$matrix.nrate_time[,1],
                        vector.nrate_rate = list.SimulationModelSettings[[1]]$matrix.nrate_rate[,1],
                        vector.STACK_lrate_nodes = list.SimulationModelSettings[[1]]$matrix.STACK_lrate_no
                        vector.STACK_lrate_rates = list.SimulationModelSettings[[1]]$matrix.STACK_lrate_ra
                        vector.STACK_AncShiftNode = list.SimulationModelSettings[[1]]$matrix.STACK_AncShif
                        vector.STACK_AncShiftValue = list.SimulationModelSettings[[1]]$matrix.STACK_AncShi
                         matrix.R = list.SimulationModelSettings[[1]]$list.Rmatrix[[1]])
```

`Simulate.TraitTrain.OU_00`

Simulate.TraitTrain.OU_00: Function to simulate trait data under OU model

---

Description

This function returns a Matrix of simulated trait data. Species are rows, traits are columns in matrix.TraitData

Usage

```
Simulate.TraitTrain.OU_00(
  handle.Phylogeny,
  numeric.Sig2,
  numeric.Alpha,
  numeric.AncestralState,
  matrix.R
)
```

Arguments

`handle.Phylogeny`
: Phylogeny used to simulate training data under OU model

`numeric.Sig2`
: Numeric Value of the sigsg (evolutionary rate) parmater of the OU model

`numeric.AncestralState`
: Numerical value of the z0 (ancestral state) parameter of the OU model

`matrix.R`
: Matrix specifying the among trait covariance for p traits. Can also be a single value (1) for a single trait

Value

matrix.TraitData Matrix of simulated trait data. Species are rows, traits are columns

Examples

```
#####################
# load dependencies #
#####################
library(geiger); library(phytools); library(TraitTrainR)

#########################
# get example phylogeny #
#########################
handle.PrimatePhylogeny <- read.tree(text = "((((((((human: 6, chimp:6): 1, gorilla: 7): 7, orangutan: 14): 11,

#############################
# Simulation Model Settings #
#############################
list.SimulationModelSettings <- list() # define an empty model list

##########################
# SET SIMULATION MODEL 1 #
##########################
numeric.NumberTrainingReps <- 10 # same number of replicates for all models in list.SimulationModelSettings
list.Rmatrix <- list(); for (i in 1:numeric.NumberTrainingReps){list.Rmatrix[[i]] <- matrix(c(1, 0, 0, 0, 1, 0,
list.Rmatrix <- list(); for (i in 1:numeric.NumberTrainingReps){list.Rmatrix[[i]] <- 1} # single trait

#############################
# append to simulation list #
#############################
list.SimulationModelSettings[[1]] <- list(string.SimulationModel = "OU",
                            vector.Sig2 = rexp(n = numeric.NumberTrainingReps, rate = 0.1),
                            vector.AncestralState = runif(n = numeric.NumberTrainingReps, min = 0, max = 100),
                            vector.Alpha <- runif(n = numeric.NumberTrainingReps, min = exp(-500), max = exp(1
```

```
                                              list.Rmatrix = list.Rmatrix)

###########################
# simulate under OU model #
###########################
Simulate.TraitTrain.OU_00(handle.Phylogeny = handle.PrimatePhylogeny,
                        numeric.Sig2 = list.SimulationModelSettings[[1]]$vector.Sig2[1],
                 numeric.AncestralState = list.SimulationModelSettings[[1]]$vector.AncestralState[1],
                      numeric.Alpha = list.SimulationModelSettings[[1]]$vector.Alpha[1],
                         matrix.R = list.SimulationModelSettings[[1]]$list.Rmatrix[[1]])
```

Simulate.TraitTrain.OU_STACKED_01
Simulate.TraitTrain.OU_STACKED_01: Function to simulate trait data under the STACK (shifted) OU model

---

Description

This function returns a Matrix of simulated trait data. Species are rows, traits are columns in matrix.TraitData

Usage

```
Simulate.TraitTrain.OU_STACKED_01(
  numeric.NumberOfSpecies,
  handle.Phylogeny,
  numeric.Sig2,
  numeric.Alpha,
  numeric.AncestralState,
  vector.STACK_AncShiftNode,
  vector.STACK_AncShiftValue,
  matrix.R
)
```

Arguments

`numeric.NumberOfSpecies`
: Numeric counting the number of species

`handle.Phylogeny`
: Phylogeny used to simulate training data under OU model

`numeric.Sig2`
: Numeric Value of the sigsg (evolutionary rate) parmater of the OU model

`numeric.AncestralState`
: Numerical value of the z0 (ancestral state) parameter of the OU model

`vector.STACK_AncShiftNode`
: Vector that includes all nodes for a STACKED AncShift model (on top of OU)

`vector.STACK_AncShiftValue`
: Vector that includes all shift values for each node in vector.STACK_AncShiftNode for a STACKED AncShift model (on top of OU)

`matrix.R`
: Matrix specifying the among trait covariance for p traits. Can also be a single value (1) for a single trait

Value

matrix.TraitData Matrix of simulated trait data. Species are rows, traits are columns

Examples

```
#####################
# load dependencies #
#####################
library(geiger); library(phytools); library(TraitTrainR)

#########################
# get example phylogeny #
#########################
handle.PrimatePhylogeny <- read.tree(text = "((((((((human: 6, chimp:6): 1, gorilla: 7): 7, orangutan: 14): 11,

#############################
# Simulation Model Settings #
#############################
list.SimulationModelSettings <- list() # define an empty model list

##########################
# SET SIMULATION MODEL 1 #
##########################
numeric.NumberTrainingReps <- 10 # same number of replicates for all models in list.SimulationModelSettings
matrix.STACK_AncShiftNode <- replicate(n = numeric.NumberTrainingReps, sample(size = 2, x = 1:16, replace = F))
matrix.STACK_AncShiftValue <- replicate(n = numeric.NumberTrainingReps , rnorm(n = 2, mean = 10000))
list.Rmatrix <- list(); for (i in 1:numeric.NumberTrainingReps){list.Rmatrix[[i]] <- matrix(c(1, 0, 0, 0, 1, 0,
#list.Rmatrix <- list(); for (i in 1:numeric.NumberTrainingReps){list.Rmatrix[[i]] <- 1} # single trait
vector.Alpha <- runif(n = numeric.NumberTrainingReps, min = exp(-500), max = exp(1))# vector of parameters for t

#############################
# append to simulation list #
#############################
list.SimulationModelSettings[[1]] <- list(string.SimulationModel = "OU",
                             vector.Sig2 = rexp(n = numeric.NumberTrainingReps, rate = 1),
                            vector.AncestralState = runif(n = numeric.NumberTrainingReps, min = 0, max = 100),
                                  matrix.STACK_AncShiftNode = matrix.STACK_AncShiftNode,
                                matrix.STACK_AncShiftValue = matrix.STACK_AncShiftValue,
                                          vector.Alpha = vector.Alpha,
                                          list.Rmatrix = list.Rmatrix)

###########################################################
# simulate under BM model with STACKED 01 (BM + AncShift) #
###########################################################
Simulate.TraitTrain.OU_STACKED_01(numeric.NumberOfSpecies = length(handle.PrimatePhylogeny$tip.label),
                                  handle.Phylogeny = handle.PrimatePhylogeny,
                        numeric.Sig2 = list.SimulationModelSettings[[1]]$vector.Sig2[1],
                      numeric.AncestralState = list.SimulationModelSettings[[1]]$vector.AncestralState[1],
                      vector.STACK_AncShiftNode = list.SimulationModelSettings[[1]]$matrix.STACK_AncShiftNo
                      vector.STACK_AncShiftValue = list.SimulationModelSettings[[1]]$matrix.STACK_AncShiftV
                      numeric.Alpha = list.SimulationModelSettings[[1]]$vector.Alpha[1],
                         matrix.R = list.SimulationModelSettings[[1]]$list.Rmatrix[[1]])
```

Simulate.TraitTrain.OU_STACKED_10

Simulate.TraitTrain.OU_STACKED_10: Function to simulate trait data under the STACK (lrates) OU model

Description

This function returns a Matrix of simulated trait data. Species are rows, traits are columns in matrix.TraitData

Usage

```
Simulate.TraitTrain.OU_STACKED_10(
  handle.Phylogeny,
  numeric.Sig2,
  numeric.Alpha,
  numeric.AncestralState,
  vector.STACK_lrate_nodes,
  vector.STACK_lrate_rates,
  matrix.R
)
```

Arguments

handle.Phylogeny	Phylogeny used to simulate training data under OU model
numeric.Sig2	Numeric Value of the sigsg (evolutionary rate) parmater of the OU model
numeric.AncestralState	Numerical value of the z0 (ancestral state) parameter of the OU model
vector.STACK_lrate_nodes	Vector that includes all nodes for a STACKED lrates model (on top of OU)
vector.STACK_lrate_rates	Vector that includes all rate values for each node in vector.STACK_lrate_nodes for a STACKED lrates model (on top of OU)
matrix.R	Matrix specifying the among trait covariance for p traits. Can also be a single value (1) for a single trait

Value

matrix.TraitData Matrix of simulated trait data. Species are rows, traits are columns

Examples

```
#####################
# load dependencies #
#####################
library(geiger); library(phytools); library(TraitTrainR)

#########################
# get example phylogeny #
#########################
```

```
handle.PrimatePhylogeny <- read.tree(text = "((((((((human: 6, chimp:6): 1, gorilla: 7): 7, orangutan: 14): 11,

##############################
# Simulation Model Settings #
##############################
list.SimulationModelSettings <- list() # define an empty model list

###########################
# SET SIMULATION MODEL 1 #
###########################
numeric.NumberTrainingReps <- 10 # same number of replicates for all models in list.SimulationModelSettings
matrix.STACK_lrate_nodes <- matrix(replicate(n = numeric.NumberTrainingReps, sample(size = 1, x = 1:16, replac
matrix.STACK_lrate_rates <- matrix(replicate(n = numeric.NumberTrainingReps , runif(n = 1, min = 0, max = 100)+
list.Rmatrix <- list(); for (i in 1:numeric.NumberTrainingReps){list.Rmatrix[[i]] <- matrix(c(1, 0, 0, 0, 1, 0,
list.Rmatrix <- list(); for (i in 1:numeric.NumberTrainingReps){list.Rmatrix[[i]] <- 1} # single trait

##############################
# append to simulation list #
##############################
list.SimulationModelSettings[[1]] <- list(string.SimulationModel = "OU",
                            vector.Sig2 = rexp(n = numeric.NumberTrainingReps, rate = 0.1),
                            vector.AncestralState = runif(n = numeric.NumberTrainingReps, min = 0, max = 100),
                            vector.Alpha <- runif(n = numeric.NumberTrainingReps, min = exp(-500), max = exp(1
                            matrix.STACK_lrate_nodes = matrix.STACK_lrate_nodes, matrix.STACK_lrate_rates =
                                          list.Rmatrix = list.Rmatrix)
#
######################################################################
# simulate under OU model with STACKED 10 (OU + lrates) #
######################################################################
Simulate.TraitTrain.OU_STACKED_10(handle.Phylogeny = handle.PrimatePhylogeny,
                        numeric.Sig2 = list.SimulationModelSettings[[1]]$vector.Sig2[1],
                      numeric.AncestralState = list.SimulationModelSettings[[1]]$vector.AncestralState[1],
                      vector.STACK_lrate_nodes = list.SimulationModelSettings[[1]]$matrix.STACK_lrate_nodes
                      vector.STACK_lrate_rates = list.SimulationModelSettings[[1]]$matrix.STACK_lrate_rates
                      numeric.Alpha = list.SimulationModelSettings[[1]]$vector.Alpha[1],
                         matrix.R = list.SimulationModelSettings[[1]]$list.Rmatrix[[1]])
```

Simulate.TraitTrain.OU_STACKED_11

Simulate.TraitTrain.OU_STACKED_11: Function to simulate trait data under the STACK (lrates + shifted) OU model

---

Description

This function returns a Matrix of simulated trait data. Species are rows, traits are columns in matrix.TraitData

Usage

```
Simulate.TraitTrain.OU_STACKED_11(
  numeric.NumberOfSpecies,
  handle.Phylogeny,
  numeric.Sig2,
  numeric.Alpha,
```

```
  numeric.AncestralState,
  vector.STACK_lrate_nodes,
  vector.STACK_lrate_rates,
  vector.STACK_AncShiftNode,
  vector.STACK_AncShiftValue,
  matrix.R
)
```

Arguments

`numeric.NumberOfSpecies`
: Numeric counting the number of species

`handle.Phylogeny`
: Phylogeny used to simulate training data under OU model

`numeric.Sig2`
: Numeric Value of the sigsg (evolutionary rate) parmater of the OU model

`numeric.AncestralState`
: Numerical value of the z0 (ancestral state) parameter of the OU model

`vector.STACK_lrate_nodes`
: Vector that includes all nodes for a STACKED lrates model (on top of OU)

`vector.STACK_lrate_rates`
: Vector that includes all rate values for each node in vector.STACK_lrate_nodes for a STACKED lrates model (on top of OU)

`vector.STACK_AncShiftNode`
: Vector that includes all nodes for a STACKED AncShift model (on top of OU)

`vector.STACK_AncShiftValue`
: Vector that includes all shift values for each node in vector.STACK_AncShiftNode for a STACKED AncShift model (on top of OU)

`matrix.R`
: Matrix specifying the among trait covariance for p traits. Can also be a single value (1) for a single trait

Value

matrix.TraitData Matrix of simulated trait data. Species are rows, traits are columns

Examples

```
#####################
# load dependencies #
#####################
library(geiger); library(phytools); library(TraitTrainR)

#########################
# get example phylogeny #
#########################
handle.PrimatePhylogeny <- read.tree(text = "((((((((human: 6, chimp:6): 1, gorilla: 7): 7, orangutan: 14): 11,

#############################
# Simulation Model Settings #
#############################
list.SimulationModelSettings <- list() # define an empty model list

##########################
# SET SIMULATION MODEL 1 #
```

```
##########################
numeric.NumberTrainingReps <- 10 # same number of replicates for all models in list.SimulationModelSettings
matrix.STACK_lrate_nodes <- matrix(replicate(n = numeric.NumberTrainingReps, sample(size = 1, x = 1:16, replac
matrix.STACK_lrate_rates <- matrix(replicate(n = numeric.NumberTrainingReps , runif(n = 1, min = 0, max = 100))
matrix.STACK_AncShiftNode <- replicate(n = numeric.NumberTrainingReps, sample(size = 2, x = 1:16, replace = F))
matrix.STACK_AncShiftValue <- replicate(n = numeric.NumberTrainingReps , rnorm(n = 2, mean = 10000))
list.Rmatrix <- list(); for (i in 1:numeric.NumberTrainingReps){list.Rmatrix[[i]] <- matrix(c(1, 0, 0, 0, 1, 0,
#list.Rmatrix <- list(); for (i in 1:numeric.NumberTrainingReps){list.Rmatrix[[i]] <- 1} # single trait
vector.Alpha <- runif(n = numeric.NumberTrainingReps, min = exp(-500), max = exp(1))# vector of parameters for t

#############################
# append to simulation list #
#############################
list.SimulationModelSettings[[1]] <- list(string.SimulationModel = "OU",
                              vector.Sig2 = rexp(n = numeric.NumberTrainingReps, rate = 1),
                             vector.AncestralState = runif(n = numeric.NumberTrainingReps, min = 0, max = 100),
                                     matrix.STACK_lrate_nodes = matrix.STACK_lrate_nodes,
                                     matrix.STACK_lrate_rates = matrix.STACK_lrate_rates,
                                   matrix.STACK_AncShiftNode = matrix.STACK_AncShiftNode,
                                 matrix.STACK_AncShiftValue = matrix.STACK_AncShiftValue,
                                            vector.Alpha = vector.Alpha,
                                            list.Rmatrix = list.Rmatrix)

#####################################################################
# simulate under OU model with STACKED 11 (OU + lrates + AncShift) #
#####################################################################
Simulate.TraitTrain.OU_STACKED_11(numeric.NumberOfSpecies = length(handle.PrimatePhylogeny$tip.label),
                                  handle.Phylogeny = handle.PrimatePhylogeny,
                        numeric.Sig2 = list.SimulationModelSettings[[1]]$vector.Sig2[1],
                      numeric.AncestralState = list.SimulationModelSettings[[1]]$vector.AncestralState[1],
                      vector.STACK_lrate_nodes = list.SimulationModelSettings[[1]]$matrix.STACK_lrate_nodes
                      vector.STACK_lrate_rates = list.SimulationModelSettings[[1]]$matrix.STACK_lrate_rates
                      vector.STACK_AncShiftNode = list.SimulationModelSettings[[1]]$matrix.STACK_AncShiftNo
                      vector.STACK_AncShiftValue = list.SimulationModelSettings[[1]]$matrix.STACK_AncShiftV
                      numeric.Alpha = list.SimulationModelSettings[[1]]$vector.Alpha[1],
                         matrix.R = list.SimulationModelSettings[[1]]$list.Rmatrix[[1]])
```

`Simulate.TraitTrain.trend_00` *Simulate.TraitTrain.trend_00: Function to simulate trait data under the trend model*

Description

This function returns a Matrix of simulated trait data. Species are rows, traits are columns in matrix.TraitData

Usage

```
Simulate.TraitTrain.trend_00(
  handle.Phylogeny,
  numeric.Sig2,
  numeric.AncestralState,
  numeric.slope,
```

```
  matrix.R
)
```

Arguments

handle.Phylogeny
: Phylogeny used to simulate training data under trend model

numeric.Sig2
: Numeric Value of the sigsg (evolutionary rate) parmater of the trend model

numeric.AncestralState
: Numerical value of the z0 (ancestral state) parameter of the trend model

matrix.R
: Matrix specifying the among trait covariance for p traits. Can also be a single value (1) for a single trait

Value

matrix.TraitData Matrix of simulated trait data. Species are rows, traits are columns

Examples

```
#############
# Reinstall #
#############
rm(list = ls()); setwd("~/OneDrive/ARCHIVE/_Software/"); uninstall("TraitTrainR/"); setwd("./TraitTrainR/");

#####################
# load dependencies #
#####################
library(geiger); library(phytools); library(TraitTrainR)

########################
# get example phylogeny #
########################
handle.PrimatePhylogeny <- read.tree(text = "((((((((human: 6, chimp:6): 1, gorilla: 7): 7, orangutan: 14): 11,

#############################
# Simulation Model Settings #
#############################
list.SimulationModelSettings <- list() # define an empty model list

##########################
# SET SIMULATION MODEL 1 #
##########################
numeric.NumberTrainingReps <- 10 # same number of replicates for all models in list.SimulationModelSettings
list.Rmatrix <- list(); for (i in 1:numeric.NumberTrainingReps){list.Rmatrix[[i]] <- matrix(c(1, 0, 0, 0, 1, 0,
#list.Rmatrix <- list(); for (i in 1:numeric.NumberTrainingReps){list.Rmatrix[[i]] <- 1} # single trait

#############################
# append to simulation list #
#############################
list.SimulationModelSettings[[1]] <- list(string.SimulationModel = "trend",
                              vector.Sig2 = rexp(n = numeric.NumberTrainingReps, rate = 1),
                            vector.AncestralState = runif(n = numeric.NumberTrainingReps, min = 0, max = 100),
                            vector.slope = rexp(n = numeric.NumberTrainingReps, rate = 1),
                                          list.Rmatrix = list.Rmatrix)

##########################################################################
```

```
# simulate under trend model with STACKED 11 (trend + lrates + AncShift) #
##########################################################################
Simulate.TraitTrain.trend_00(handle.Phylogeny = handle.PrimatePhylogeny,
                           numeric.Sig2 = list.SimulationModelSettings[[1]]$vector.Sig2[1],
                      numeric.AncestralState = list.SimulationModelSettings[[1]]$vector.AncestralState[1],
                         numeric.slope = list.SimulationModelSettings[[1]]$vector.slope[1],
                            matrix.R = list.SimulationModelSettings[[1]]$list.Rmatrix[[1]])
```

Simulate.TraitTrain.trend_STACKED_01
Simulate.TraitTrain.trend_STACKED_01: Function to simulate trait data under the STACK (+AncShift) trend model

---

Description

This function returns a Matrix of simulated trait data. Species are rows, traits are columns in matrix.TraitData

Usage

```
Simulate.TraitTrain.trend_STACKED_01(
  numeric.NumberOfSpecies,
  handle.Phylogeny,
  numeric.Sig2,
  numeric.AncestralState,
  numeric.slope,
  vector.STACK_AncShiftNode,
  vector.STACK_AncShiftValue,
  matrix.R
)
```

Arguments

`numeric.NumberOfSpecies`
: Numeric counting the number of species

`handle.Phylogeny`
: Phylogeny used to simulate training data under trend model

`numeric.Sig2`
: Numeric Value of the sigsg (evolutionary rate) parmater of the trend model

`numeric.AncestralState`
: Numerical value of the z0 (ancestral state) parameter of the trend model

`vector.STACK_AncShiftNode`
: Vector that includes all nodes for a STACKED AncShift model (on top of trend)

`vector.STACK_AncShiftValue`
: Vector that includes all shift values for each node in vector.STACK_AncShiftNode for a STACKED AncShift model (on top of trend)

`matrix.R`
: Matrix specifying the among trait covariance for p traits. Can also be a single value (1) for a single trait

Value

matrix.TraitData Matrix of simulated trait data. Species are rows, traits are columns

Examples

```
#####################
# load dependencies #
#####################
library(geiger); library(phytools); library(TraitTrainR)

#########################
# get example phylogeny #
#########################
handle.PrimatePhylogeny <- read.tree(text = "((((((((human: 6, chimp:6): 1, gorilla: 7): 7, orangutan: 14): 11,

#############################
# Simulation Model Settings #
#############################
list.SimulationModelSettings <- list() # define an empty model list

##########################
# SET SIMULATION MODEL 1 #
##########################
numeric.NumberTrainingReps <- 10 # same number of replicates for all models in list.SimulationModelSettings
matrix.STACK_AncShiftNode <- replicate(n = numeric.NumberTrainingReps, sample(size = 2, x = 1:16, replace = F))
matrix.STACK_AncShiftValue <- replicate(n = numeric.NumberTrainingReps , rnorm(n = 2, mean = 10000))
list.Rmatrix <- list(); for (i in 1:numeric.NumberTrainingReps){list.Rmatrix[[i]] <- matrix(c(1, 0, 0, 0, 1, 0,
#list.Rmatrix <- list(); for (i in 1:numeric.NumberTrainingReps){list.Rmatrix[[i]] <- 1} # single trait

#############################
# append to simulation list #
#############################
list.SimulationModelSettings[[1]] <- list(string.SimulationModel = "trend",
                             vector.Sig2 = rexp(n = numeric.NumberTrainingReps, rate = 1),
                            vector.AncestralState = runif(n = numeric.NumberTrainingReps, min = 0, max = 100),
                                  matrix.STACK_AncShiftNode = matrix.STACK_AncShiftNode,
                                matrix.STACK_AncShiftValue = matrix.STACK_AncShiftValue,
                            vector.slope = rexp(n = numeric.NumberTrainingReps, rate = 1),
                                        list.Rmatrix = list.Rmatrix)

##########################################################################
# simulate under trend model with STACKED 11 (trend + lrates + AncShift) #
##########################################################################
Simulate.TraitTrain.trend_STACKED_01(numeric.NumberOfSpecies = length(handle.PrimatePhylogeny$tip.label),
                                     handle.Phylogeny = handle.PrimatePhylogeny,
                         numeric.Sig2 = list.SimulationModelSettings[[1]]$vector.Sig2[1],
                         numeric.AncestralState = list.SimulationModelSettings[[1]]$vector.AncestralState[1]
                         vector.STACK_AncShiftNode = list.SimulationModelSettings[[1]]$matrix.STACK_AncShift
                         vector.STACK_AncShiftValue = list.SimulationModelSettings[[1]]$matrix.STACK_AncShi
                         numeric.slope = list.SimulationModelSettings[[1]]$vector.slope[1],
                          matrix.R = list.SimulationModelSettings[[1]]$list.Rmatrix[[1]])
```

`Simulate.TraitTrain.trend_STACKED_10`

Simulate.TraitTrain.trend_STACKED_10: Function to simulate trait data under the STACK (+lrates) trend model

---

Description

This function returns a Matrix of simulated trait data. Species are rows, traits are columns in matrix.TraitData

Usage

```
Simulate.TraitTrain.trend_STACKED_10(
  handle.Phylogeny,
  numeric.Sig2,
  numeric.AncestralState,
  numeric.slope,
  vector.STACK_lrate_nodes,
  vector.STACK_lrate_rates,
  matrix.R
)
```

Arguments

`handle.Phylogeny`
: Phylogeny used to simulate training data under trend model

`numeric.Sig2`
: Numeric Value of the sigsg (evolutionary rate) parmater of the trend model

`numeric.AncestralState`
: Numerical value of the z0 (ancestral state) parameter of the trend model

`vector.STACK_lrate_nodes`
: Vector that includes all nodes for a STACKED lrates model (on top of trend)

`vector.STACK_lrate_rates`
: Vector that includes all rate values for each node in vector.STACK_lrate_nodes for a STACKED lrates model (on top of trend)

`matrix.R`
: Matrix specifying the among trait covariance for p traits. Can also be a single value (1) for a single trait

Value

matrix.TraitData Matrix of simulated trait data. Species are rows, traits are columns

Examples

```
#####################
# load dependencies #
#####################
library(geiger); library(phytools); library(TraitTrainR)

#########################
# get example phylogeny #
#########################
handle.PrimatePhylogeny <- read.tree(text = "((((((((human: 6, chimp:6): 1, gorilla: 7): 7, orangutan: 14): 11,

#############################
# Simulation Model Settings #
#############################
list.SimulationModelSettings <- list() # define an empty model list

##########################
```

```
# SET SIMULATION MODEL 1 #
##########################
numeric.NumberTrainingReps <- 10 # same number of replicates for all models in list.SimulationModelSettings
matrix.STACK_lrate_nodes <- matrix(replicate(n = numeric.NumberTrainingReps, sample(size = 1, x = 1:16, replac
matrix.STACK_lrate_rates <- matrix(replicate(n = numeric.NumberTrainingReps , runif(n = 1, min = 0, max = 100))
matrix.STACK_AncShiftNode <- replicate(n = numeric.NumberTrainingReps, sample(size = 2, x = 1:16, replace = F))
matrix.STACK_AncShiftValue <- replicate(n = numeric.NumberTrainingReps , rnorm(n = 2, mean = 10000))
list.Rmatrix <- list(); for (i in 1:numeric.NumberTrainingReps){list.Rmatrix[[i]] <- matrix(c(1, 0, 0, 0, 1, 0,
#list.Rmatrix <- list(); for (i in 1:numeric.NumberTrainingReps){list.Rmatrix[[i]] <- 1} # single trait

#############################
# append to simulation list #
#############################
list.SimulationModelSettings[[1]] <- list(string.SimulationModel = "trend",
                             vector.Sig2 = rexp(n = numeric.NumberTrainingReps, rate = 1),
                            vector.AncestralState = runif(n = numeric.NumberTrainingReps, min = 0, max = 100),
                                    matrix.STACK_lrate_nodes = matrix.STACK_lrate_nodes,
                                    matrix.STACK_lrate_rates = matrix.STACK_lrate_rates,
                            vector.slope = rexp(n = numeric.NumberTrainingReps, rate = 1),
                                          list.Rmatrix = list.Rmatrix)

##########################################################################
# simulate under trend model with STACKED 11 (trend + lrates + AncShift) #
##########################################################################
Simulate.TraitTrain.trend_STACKED_10(handle.Phylogeny = handle.PrimatePhylogeny,
                         numeric.Sig2 = list.SimulationModelSettings[[1]]$vector.Sig2[1],
                         numeric.AncestralState = list.SimulationModelSettings[[1]]$vector.AncestralState[1]
                         vector.STACK_lrate_nodes = list.SimulationModelSettings[[1]]$matrix.STACK_lrate_noc
                         vector.STACK_lrate_rates = list.SimulationModelSettings[[1]]$matrix.STACK_lrate_ra
                         numeric.slope = list.SimulationModelSettings[[1]]$vector.slope[1],
                          matrix.R = list.SimulationModelSettings[[1]]$list.Rmatrix[[1]])
```

Simulate.TraitTrain.trend_STACKED_11

Simulate.TraitTrain.trend_STACKED_11: Function to simulate trait data under the STACK (lrates + shifted) trend model

---

Description

This function returns a Matrix of simulated trait data. Species are rows, traits are columns in matrix.TraitData

Usage

```
Simulate.TraitTrain.trend_STACKED_11(
  numeric.NumberOfSpecies,
  handle.Phylogeny,
  numeric.Sig2,
  numeric.AncestralState,
  numeric.slope,
  vector.STACK_lrate_nodes,
  vector.STACK_lrate_rates,
  vector.STACK_AncShiftNode,
```

```
  vector.STACK_AncShiftValue,
  matrix.R
)
```

Arguments

`numeric.NumberOfSpecies`
: Numeric counting the number of species

`handle.Phylogeny`
: Phylogeny used to simulate training data under trend model

`numeric.Sig2`
: Numeric Value of the sigsg (evolutionary rate) parmater of the trend model

`numeric.AncestralState`
: Numerical value of the z0 (ancestral state) parameter of the trend model

`vector.STACK_lrate_nodes`
: Vector that includes all nodes for a STACKED lrates model (on top of trend)

`vector.STACK_lrate_rates`
: Vector that includes all rate values for each node in vector.STACK_lrate_nodes for a STACKED lrates model (on top of trend)

`vector.STACK_AncShiftNode`
: Vector that includes all nodes for a STACKED AncShift model (on top of trend)

`vector.STACK_AncShiftValue`
: Vector that includes all shift values for each node in vector.STACK_AncShiftNode for a STACKED AncShift model (on top of trend)

`matrix.R`
: Matrix specifying the among trait covariance for p traits. Can also be a single value (1) for a single trait

Value

matrix.TraitData Matrix of simulated trait data. Species are rows, traits are columns

Examples

```
#####################
# load dependencies #
#####################
library(geiger); library(phytools); library(TraitTrainR)

#########################
# get example phylogeny #
#########################
handle.PrimatePhylogeny <- read.tree(text = "((((((((human: 6, chimp:6): 1, gorilla: 7): 7, orangutan: 14): 11,

#############################
# Simulation Model Settings #
#############################
list.SimulationModelSettings <- list() # define an empty model list

##########################
# SET SIMULATION MODEL 1 #
##########################
numeric.NumberTrainingReps <- 10 # same number of replicates for all models in list.SimulationModelSettings
matrix.STACK_lrate_nodes <- matrix(replicate(n = numeric.NumberTrainingReps, sample(size = 1, x = 1:16, replac
matrix.STACK_lrate_rates <- matrix(replicate(n = numeric.NumberTrainingReps , runif(n = 1, min = 0, max = 100))
```

```
matrix.STACK_AncShiftNode <- replicate(n = numeric.NumberTrainingReps, sample(size = 2, x = 1:16, replace = F))
matrix.STACK_AncShiftValue <- replicate(n = numeric.NumberTrainingReps , rnorm(n = 2, mean = 10000))
list.Rmatrix <- list(); for (i in 1:numeric.NumberTrainingReps){list.Rmatrix[[i]] <- matrix(c(1, 0, 0, 0, 1, 0,
#list.Rmatrix <- list(); for (i in 1:numeric.NumberTrainingReps){list.Rmatrix[[i]] <- 1} # single trait

##############################
# append to simulation list #
##############################
list.SimulationModelSettings[[1]] <- list(string.SimulationModel = "trend",
                              vector.Sig2 = rexp(n = numeric.NumberTrainingReps, rate = 1),
                             vector.AncestralState = runif(n = numeric.NumberTrainingReps, min = 0, max = 100),
                                     matrix.STACK_lrate_nodes = matrix.STACK_lrate_nodes,
                                     matrix.STACK_lrate_rates = matrix.STACK_lrate_rates,
                                   matrix.STACK_AncShiftNode = matrix.STACK_AncShiftNode,
                                 matrix.STACK_AncShiftValue = matrix.STACK_AncShiftValue,
                             vector.slope = rexp(n = numeric.NumberTrainingReps, rate = 1),
                                           list.Rmatrix = list.Rmatrix)

##########################################################################
# simulate under trend model with STACKED 11 (trend + lrates + AncShift) #
##########################################################################
Simulate.TraitTrain.trend_STACKED_11(numeric.NumberOfSpecies = length(handle.PrimatePhylogeny$tip.label),
                                      handle.Phylogeny = handle.PrimatePhylogeny,
                         numeric.Sig2 = list.SimulationModelSettings[[1]]$vector.Sig2[1],
                         numeric.AncestralState = list.SimulationModelSettings[[1]]$vector.AncestralState[1]
                         vector.STACK_lrate_nodes = list.SimulationModelSettings[[1]]$matrix.STACK_lrate_noc
                         vector.STACK_lrate_rates = list.SimulationModelSettings[[1]]$matrix.STACK_lrate_rat
                         vector.STACK_AncShiftNode = list.SimulationModelSettings[[1]]$matrix.STACK_AncShift
                         vector.STACK_AncShiftValue = list.SimulationModelSettings[[1]]$matrix.STACK_AncShit
                         numeric.slope = list.SimulationModelSettings[[1]]$vector.slope[1],
                          matrix.R = list.SimulationModelSettings[[1]]$list.Rmatrix[[1]])
```

Simulate.TraitTrain.white_00 *Simulate.TraitTrain.white_00: Function to simulate trait data under the white noise model*

Description

This function returns a Matrix of simulated trait data. Species are rows, traits are columns in matrix.TraitData

Usage

```
Simulate.TraitTrain.white_00(
  handle.Phylogeny,
  numeric.Sig2,
  numeric.AncestralState,
  matrix.R
)
```

Arguments

handle.Phylogeny
: Phylogeny used to simulate training data under white model

numeric.Sig2
: Numeric Value of the sigsg (evolutionary rate) parmater of the white model

numeric.AncestralState
: Numerical value of the z0 (ancestral state) parameter of the white model

matrix.R
: Matrix specifying the among trait covariance for p traits. Can also be a single value (1) for a single trait

Value

matrix.TraitData Matrix of simulated trait data. Species are rows, traits are columns

Examples

```
#####################
# load dependencies #
#####################
library(geiger); library(phytools); library(TraitTrainR)

#########################
# get example phylogeny #
#########################
handle.PrimatePhylogeny <- read.tree(text = "((((((((human: 6, chimp:6): 1, gorilla: 7): 7, orangutan: 14): 11,

#############################
# Simulation Model Settings #
#############################
list.SimulationModelSettings <- list() # define an empty model list

##########################
# SET SIMULATION MODEL 1 #
##########################
numeric.NumberTrainingReps <- 10 # same number of replicates for all models in list.SimulationModelSettings
list.Rmatrix <- list(); for (i in 1:numeric.NumberTrainingReps){list.Rmatrix[[i]] <- matrix(c(1, 0, 0, 0, 1, 0,
#list.Rmatrix <- list(); for (i in 1:numeric.NumberTrainingReps){list.Rmatrix[[i]] <- 1} # single trait

#############################
# append to simulation list #
#############################
list.SimulationModelSettings[[1]] <- list(string.SimulationModel = "white",
                             vector.Sig2 = rexp(n = numeric.NumberTrainingReps, rate = 1),
                            vector.AncestralState = runif(n = numeric.NumberTrainingReps, min = 0, max = 100),
                                          list.Rmatrix = list.Rmatrix)

####################################
# simulate under white noise model #
####################################
Simulate.TraitTrain.white_00(numeric.NumberOfSpecies = length(handle.PrimatePhylogeny$tip.label),
                    handle.Phylogeny = handle.PrimatePhylogeny, numeric.Sig2 = list.SimulationModelSettings[[
                    numeric.AncestralState = list.SimulationModelSettings[[1]]$vector.AncestralState[1],
                          matrix.R = list.SimulationModelSettings[[1]]$list.Rmatrix[[1]])
```

`Simulate.TraitTrain.white_STACKED_01`

Simulate.TraitTrain.white_STACKED_01: Function to simulate trait data under the STACK (+AncShift) white model

Description

This function returns a Matrix of simulated trait data. Species are rows, traits are columns in matrix.TraitData

Usage

```
Simulate.TraitTrain.white_STACKED_01(
  numeric.NumberOfSpecies,
  handle.Phylogeny,
  numeric.Sig2,
  numeric.AncestralState,
  vector.STACK_AncShiftNode,
  vector.STACK_AncShiftValue,
  matrix.R
)
```

Arguments

`numeric.NumberOfSpecies`
: Numeric counting the number of species

`handle.Phylogeny`
: Phylogeny used to simulate training data under white model

`numeric.Sig2`
: Numeric Value of the sigsg (evolutionary rate) parmater of the white model

`numeric.AncestralState`
: Numerical value of the z0 (ancestral state) parameter of the white model

`vector.STACK_AncShiftNode`
: Vector that includes all nodes for a STACKED AncShift model (on top of white)

`vector.STACK_AncShiftValue`
: Vector that includes all shift values for each node in vector.STACK_AncShiftNode for a STACKED AncShift model (on top of white)

`matrix.R`
: Matrix specifying the among trait covariance for p traits. Can also be a single value (1) for a single trait

Value

matrix.TraitData Matrix of simulated trait data. Species are rows, traits are columns

Examples

```
#####################
# load dependencies #
#####################
library(geiger); library(phytools); library(TraitTrainR)
```

```
##########################
# get example phylogeny #
##########################
handle.PrimatePhylogeny <- read.tree(text = "((((((((human: 6, chimp:6): 1, gorilla: 7): 7, orangutan: 14): 11,

##############################
# Simulation Model Settings #
##############################
list.SimulationModelSettings <- list() # define an empty model list

###########################
# SET SIMULATION MODEL 1 #
###########################
numeric.NumberTrainingReps <- 10 # same number of replicates for all models in list.SimulationModelSettings
matrix.STACK_AncShiftNode <- replicate(n = numeric.NumberTrainingReps, sample(size = 2, x = 1:16, replace = F))
matrix.STACK_AncShiftValue <- replicate(n = numeric.NumberTrainingReps , rnorm(n = 2, mean = 10000))
list.Rmatrix <- list(); for (i in 1:numeric.NumberTrainingReps){list.Rmatrix[[i]] <- matrix(c(1, 0, 0, 0, 1, 0,
#list.Rmatrix <- list(); for (i in 1:numeric.NumberTrainingReps){list.Rmatrix[[i]] <- 1} # single trait

##############################
# append to simulation list #
##############################
list.SimulationModelSettings[[1]] <- list(string.SimulationModel = "white", vector.Sig2 = rexp(n = numeric.Nur
                            matrix.STACK_AncShiftNode = matrix.STACK_AncShiftNode, matrix.STACK_AncShiftValu
                                          list.Rmatrix = list.Rmatrix)

###############################################################################
# simulate under white model with STACKED 01 (whitenoise + AncShift) #
###############################################################################
Simulate.TraitTrain.white_STACKED_01(numeric.NumberOfSpecies = length(handle.PrimatePhylogeny$tip.label), ha
                         vector.STACK_AncShiftNode = list.SimulationModelSettings[[1]]$matrix.STACK_AncShif
                          matrix.R = list.SimulationModelSettings[[1]]$list.Rmatrix[[1]])
```

Simulate.TraitTrain.white_STACKED_10

Simulate.TraitTrain.white_STACKED_10: Function to simulate trait data under the STACK (+lrates) white model

---

Description

This function returns a Matrix of simulated trait data. Species are rows, traits are columns in matrix.TraitData

Usage

```
Simulate.TraitTrain.white_STACKED_10(
  handle.Phylogeny,
  numeric.Sig2,
  numeric.AncestralState,
  vector.STACK_lrate_nodes,
  vector.STACK_lrate_rates,
  matrix.R
)
```

Arguments

`handle.Phylogeny`
: Phylogeny used to simulate training data under white model

`numeric.Sig2`
: Numeric Value of the sigsg (evolutionary rate) parmater of the white model

`numeric.AncestralState`
: Numerical value of the z0 (ancestral state) parameter of the white model

`vector.STACK_lrate_nodes`
: Vector that includes all nodes for a STACKED lrates model (on top of white)

`vector.STACK_lrate_rates`
: Vector that includes all rate values for each node in vector.STACK_lrate_nodes for a STACKED lrates model (on top of white)

`matrix.R`
: Matrix specifying the among trait covariance for p traits. Can also be a single value (1) for a single trait

Value

matrix.TraitData Matrix of simulated trait data. Species are rows, traits are columns

Examples

```
#####################
# load dependencies #
#####################
library(geiger); library(phytools); library(TraitTrainR)

#########################
# get example phylogeny #
#########################
handle.PrimatePhylogeny <- read.tree(text = "((((((((human: 6, chimp:6): 1, gorilla: 7): 7, orangutan: 14): 11,

#############################
# Simulation Model Settings #
#############################
list.SimulationModelSettings <- list() # define an empty model list

##########################
# SET SIMULATION MODEL 1 #
##########################
numeric.NumberTrainingReps <- 10 # same number of replicates for all models in list.SimulationModelSettings
matrix.STACK_lrate_nodes <- matrix(replicate(n = numeric.NumberTrainingReps, sample(size = 1, x = 1:16, replac
matrix.STACK_lrate_rates <- matrix(replicate(n = numeric.NumberTrainingReps , runif(n = 1, min = 0, max = 100))
list.Rmatrix <- list(); for (i in 1:numeric.NumberTrainingReps){list.Rmatrix[[i]] <- matrix(c(1, 0, 0, 0, 1, 0,
#list.Rmatrix <- list(); for (i in 1:numeric.NumberTrainingReps){list.Rmatrix[[i]] <- 1} # single trait

#############################
# append to simulation list #
#############################
list.SimulationModelSettings[[1]] <- list(string.SimulationModel = "white", vector.Sig2 = rexp(n = numeric.Nur
                            matrix.STACK_lrate_nodes = matrix.STACK_lrate_nodes, matrix.STACK_lrate_rates = 
                                          list.Rmatrix = list.Rmatrix)

###################################################################
# simulate under white model with STACKED 10 (whitenoise + lrates #
###################################################################
```

```
Simulate.TraitTrain.white_STACKED_10(handle.Phylogeny = handle.PrimatePhylogeny, numeric.Sig2 = list.Simulat
                              vector.STACK_lrate_nodes = list.SimulationModelSettings[[1]]$matrix.STACK_lrate_noc
                               matrix.R = list.SimulationModelSettings[[1]]$list.Rmatrix[[1]])
```

Simulate.TraitTrain.white_STACKED_11

Simulate.TraitTrain.white_STACKED_11: Function to simulate trait data under the STACK (lrates + shifted) white model

---

Description

This function returns a Matrix of simulated trait data. Species are rows, traits are columns in matrix.TraitData

Usage

```
Simulate.TraitTrain.white_STACKED_11(
  numeric.NumberOfSpecies,
  handle.Phylogeny,
  numeric.Sig2,
  numeric.AncestralState,
  vector.STACK_lrate_nodes,
  vector.STACK_lrate_rates,
  vector.STACK_AncShiftNode,
  vector.STACK_AncShiftValue,
  matrix.R
)
```

Arguments

`numeric.NumberOfSpecies`
: Numeric counting the number of species

`handle.Phylogeny`
: Phylogeny used to simulate training data under white model

`numeric.Sig2`
: Numeric Value of the sigsg (evolutionary rate) parmater of the white model

`numeric.AncestralState`
: Numerical value of the z0 (ancestral state) parameter of the white model

`vector.STACK_lrate_nodes`
: Vector that includes all nodes for a STACKED lrates model (on top of white)

`vector.STACK_lrate_rates`
: Vector that includes all rate values for each node in vector.STACK_lrate_nodes for a STACKED lrates model (on top of white)

`vector.STACK_AncShiftNode`
: Vector that includes all nodes for a STACKED AncShift model (on top of white)

`vector.STACK_AncShiftValue`
: Vector that includes all shift values for each node in vector.STACK_AncShiftNode for a STACKED AncShift model (on top of white)

`matrix.R`
: Matrix specifying the among trait covariance for p traits. Can also be a single value (1) for a single trait

Value

matrix.TraitData Matrix of simulated trait data. Species are rows, traits are columns

Examples

```
#####################
# load dependencies #
#####################
library(geiger); library(phytools); library(TraitTrainR)

#########################
# get example phylogeny #
#########################
handle.PrimatePhylogeny <- read.tree(text = "((((((((human: 6, chimp:6): 1, gorilla: 7): 7, orangutan: 14): 11,

#############################
# Simulation Model Settings #
#############################
list.SimulationModelSettings <- list() # define an empty model list

##########################
# SET SIMULATION MODEL 1 #
##########################
numeric.NumberTrainingReps <- 10 # same number of replicates for all models in list.SimulationModelSettings
matrix.STACK_lrate_nodes <- matrix(replicate(n = numeric.NumberTrainingReps, sample(size = 1, x = 1:16, replac
matrix.STACK_lrate_rates <- matrix(replicate(n = numeric.NumberTrainingReps , runif(n = 1, min = 0, max = 100))
matrix.STACK_AncShiftNode <- replicate(n = numeric.NumberTrainingReps, sample(size = 2, x = 1:16, replace = F))
matrix.STACK_AncShiftValue <- replicate(n = numeric.NumberTrainingReps , rnorm(n = 2, mean = 10000))
list.Rmatrix <- list(); for (i in 1:numeric.NumberTrainingReps){list.Rmatrix[[i]] <- matrix(c(1, 0, 0, 0, 1, 0,
#list.Rmatrix <- list(); for (i in 1:numeric.NumberTrainingReps){list.Rmatrix[[i]] <- 1} # single trait

#############################
# append to simulation list #
#############################
list.SimulationModelSettings[[1]] <- list(string.SimulationModel = "white", vector.Sig2 = rexp(n = numeric.Nur
                            matrix.STACK_lrate_nodes = matrix.STACK_lrate_nodes, matrix.STACK_lrate_rates =
                            matrix.STACK_AncShiftNode = matrix.STACK_AncShiftNode, matrix.STACK_AncShiftValu
                                          list.Rmatrix = list.Rmatrix)

##################################################################################
# simulate under white model with STACKED 11 (whitenoise + lrates + AncShift) #
##################################################################################
Simulate.TraitTrain.white_STACKED_11(numeric.NumberOfSpecies = length(handle.PrimatePhylogeny$tip.label), ha
                         vector.STACK_lrate_nodes = list.SimulationModelSettings[[1]]$matrix.STACK_lrate_noc
                         vector.STACK_AncShiftNode = list.SimulationModelSettings[[1]]$matrix.STACK_AncShif
                          matrix.R = list.SimulationModelSettings[[1]]$list.Rmatrix[[1]])
```

`TraitTrain` *TraitTrain: Function to conduct large-scale, flexible simulations of trait data given a particular tree.*

Description

This function returns a list of data.frames that include: RESULTS_TRAIT (raw trait values across simulations); RESULTS_PIC (compute PICS across simulations); RESULTS_PIC_DEPTH (computed PICS across simulation using target tree scaled to depth); RESULTS_PROJECT (projected trait using input tree); RESULTS_PROJECT_DEPTH (projected traits using input tree scaled to unit depth = 1)

Usage

```
TraitTrain(
  handle.Phylogeny,
  list.SimulationModelSettings,
  numeric.MeasurementError = 0,
  logical.PIC = F,
  logical.PROJECT = F
)
```

Arguments

`handle.Phylogeny`
: Phylogeny used to simulate traits with TrainTrainR

`list.SimulationModelSettings`
: List containing the model values and parameters to be used during simulation. Must contain (at least) the following for each entry in the list: string.SimulationModel (string defines the model; must be one of c("BM", "OU", "EB", "nrate", "lrate", "trend", "lambda", "kappa", "delta", "white", "depth")); vector.Sig2 (value of the evolutionary rate parameter for each replicate); vector.AncestralState (value of the ancestral state for each replicate)

`numeric.MeasurementError`
: Numerical value representing the variance of the sample error (assumed Normally distributed with mean = 0 , sd = sqrt(numeric.MeasurementError))

`logical.PIC`
: True/False logical indicating whether to compute PICs (or not).

`logical.PROJECT`
: True/False logical indicating whether to compute phylogenetic projections (or not)

Value

LIST A list of data.frames that include: RESULTS_TRAIT (raw trait values across simulations); RESULTS_PIC (compute PICS across simulations); RESULTS_PIC_DEPTH (computed PICS across simulation using target tree scaled to depth); RESULTS_PROJECT (projected trait using input tree); RESULTS_PROJECT_DEPTH (projected traits using input tree scaled to unit depth = 1)

Examples

```
#####################
# load dependencies #
#####################
library(geiger); library(phytools); library(TraitTrainR)

#########################
# get example phylogeny #
#########################
```

```
handle.PrimatePhylogeny <- read.tree(text = "((((((((human: 6, chimp:6): 1, gorilla: 7): 7, orangutan: 14): 11,

##############################
# Simulation Model Settings #
##############################
list.SimulationModelSettings <- list() # define an empty model list

###########################
# SET SIMULATION MODEL 1 #
###########################
numeric.NumberTrainingReps <- 2 # same number of replicates for all models in list.SimulationModelSettings
list.Rmatrix <- list(); for (i in 1:numeric.NumberTrainingReps){list.Rmatrix[[i]] <- matrix(c(1, 0, 0, 0, 1, 0,
#list.Rmatrix <- list(); for (i in 1:numeric.NumberTrainingReps){list.Rmatrix[[i]] <- matrix(1, nrow = 1, ncol

######################
# First model is BM  #
######################
list.SimulationModelSettings[[1]] <- list(string.SimulationModel = "BM",
                             vector.Sig2 = rexp(n = numeric.NumberTrainingReps, rate = 1),
                            vector.AncestralState = runif(n = numeric.NumberTrainingReps, min = 0, max = 100),
                            matrix.STACK_lrate_nodes = matrix(replicate(n = numeric.NumberTrainingReps, samp
                            matrix.STACK_lrate_rates = matrix(replicate(n = numeric.NumberTrainingReps , run
                            matrix.STACK_AncShiftNode = replicate(n = numeric.NumberTrainingReps, sample(siz
                            matrix.STACK_AncShiftValue = replicate(n = numeric.NumberTrainingReps , rnorm(n =
                                          list.Rmatrix = list.Rmatrix)

#######################
# Second model is OU  #
#######################
list.SimulationModelSettings[[2]] <- list(string.SimulationModel = "OU",
                             vector.Sig2 = rexp(n = numeric.NumberTrainingReps, rate = 1),
                            vector.AncestralState = runif(n = numeric.NumberTrainingReps, min = 0, max = 100),
                            matrix.STACK_lrate_nodes = matrix(replicate(n = numeric.NumberTrainingReps, samp
                            matrix.STACK_lrate_rates = matrix(replicate(n = numeric.NumberTrainingReps , run
                            matrix.STACK_AncShiftNode = replicate(n = numeric.NumberTrainingReps, sample(siz
                            matrix.STACK_AncShiftValue = replicate(n = numeric.NumberTrainingReps , rnorm(n =
                            vector.Alpha = runif(n = numeric.NumberTrainingReps, min = exp(-500), max = exp(1)
                                          list.Rmatrix = list.Rmatrix)


######################
# third model is EB  #
######################
list.SimulationModelSettings[[3]] <- list(string.SimulationModel = "EB",
                             vector.Sig2 = rexp(n = numeric.NumberTrainingReps, rate = 1),
                            vector.AncestralState = runif(n = numeric.NumberTrainingReps, min = 0, max = 100),
                            matrix.STACK_lrate_nodes = matrix(replicate(n = numeric.NumberTrainingReps, samp
                            matrix.STACK_lrate_rates = matrix(replicate(n = numeric.NumberTrainingReps , run
                            matrix.STACK_AncShiftNode = replicate(n = numeric.NumberTrainingReps, sample(siz
                            matrix.STACK_AncShiftValue = replicate(n = numeric.NumberTrainingReps , rnorm(n =
                            vector.A = runif(n = numeric.NumberTrainingReps, min = log(10^-5)/310, max = -0.00
                                          list.Rmatrix = list.Rmatrix)

##########################
# fourth model is kappa  #
##########################
list.SimulationModelSettings[[4]] <- list(string.SimulationModel = "kappa",
```

```
                             vector.Sig2 = rexp(n = numeric.NumberTrainingReps, rate = 1),
                            vector.AncestralState = runif(n = numeric.NumberTrainingReps, min = 0, max = 100),
                            matrix.STACK_lrate_nodes = matrix(replicate(n = numeric.NumberTrainingReps, samp
                            matrix.STACK_lrate_rates = matrix(replicate(n = numeric.NumberTrainingReps , run
                            matrix.STACK_AncShiftNode = replicate(n = numeric.NumberTrainingReps, sample(siz
                            matrix.STACK_AncShiftValue = replicate(n = numeric.NumberTrainingReps , rnorm(n =
                            vector.kappa = runif(n = numeric.NumberTrainingReps, min = exp(-500), max = 1),
                                          list.Rmatrix = list.Rmatrix)
##########################
# fifth model is lambda  #
##########################
list.SimulationModelSettings[[5]] <- list(string.SimulationModel = "lambda",
                             vector.Sig2 = rexp(n = numeric.NumberTrainingReps, rate = 1),
                            vector.AncestralState = runif(n = numeric.NumberTrainingReps, min = 0, max = 100),
                            matrix.STACK_lrate_nodes = matrix(replicate(n = numeric.NumberTrainingReps, samp
                            matrix.STACK_lrate_rates = matrix(replicate(n = numeric.NumberTrainingReps , run
                            matrix.STACK_AncShiftNode = replicate(n = numeric.NumberTrainingReps, sample(siz
                            matrix.STACK_AncShiftValue = replicate(n = numeric.NumberTrainingReps , rnorm(n =
                            vector.lambda = runif(n = numeric.NumberTrainingReps, min = exp(-500), max = 1),
                                          list.Rmatrix = list.Rmatrix)

#######################
# six model is delta  #
#######################
list.SimulationModelSettings[[6]] <- list(string.SimulationModel = "delta",
                             vector.Sig2 = rexp(n = numeric.NumberTrainingReps, rate = 1),
                            vector.AncestralState = runif(n = numeric.NumberTrainingReps, min = 0, max = 100),
                            matrix.STACK_lrate_nodes = matrix(replicate(n = numeric.NumberTrainingReps, samp
                            matrix.STACK_lrate_rates = matrix(replicate(n = numeric.NumberTrainingReps , run
                            matrix.STACK_AncShiftNode = replicate(n = numeric.NumberTrainingReps, sample(siz
                            matrix.STACK_AncShiftValue = replicate(n = numeric.NumberTrainingReps , rnorm(n =
                            vector.delta = runif(n = numeric.NumberTrainingReps, min = exp(-500), max = 3),
                                          list.Rmatrix = list.Rmatrix)


###########################
# seventh model is trend  #
###########################
list.SimulationModelSettings[[7]] <- list(string.SimulationModel = "trend",
                             vector.Sig2 = rexp(n = numeric.NumberTrainingReps, rate = 1),
                            vector.AncestralState = runif(n = numeric.NumberTrainingReps, min = 0, max = 100),
                            matrix.STACK_lrate_nodes = matrix(replicate(n = numeric.NumberTrainingReps, samp
                            matrix.STACK_lrate_rates = matrix(replicate(n = numeric.NumberTrainingReps , run
                            matrix.STACK_AncShiftNode = replicate(n = numeric.NumberTrainingReps, sample(siz
                            matrix.STACK_AncShiftValue = replicate(n = numeric.NumberTrainingReps , rnorm(n =
                             vector.slope = rexp(n = numeric.NumberTrainingReps, rate = 1),
                                          list.Rmatrix = list.Rmatrix)
#########################
# eight model is white  #
#########################
list.SimulationModelSettings[[8]] <- list(string.SimulationModel = "white",
                             vector.Sig2 = rexp(n = numeric.NumberTrainingReps, rate = 1),
                            vector.AncestralState = runif(n = numeric.NumberTrainingReps, min = 0, max = 100),
                            matrix.STACK_lrate_nodes = matrix(replicate(n = numeric.NumberTrainingReps, samp
                            matrix.STACK_lrate_rates = matrix(replicate(n = numeric.NumberTrainingReps , run
                            matrix.STACK_AncShiftNode = replicate(n = numeric.NumberTrainingReps, sample(siz
                            matrix.STACK_AncShiftValue = replicate(n = numeric.NumberTrainingReps , rnorm(n =
```

```
                                         list.Rmatrix = list.Rmatrix)


##########################
# nineth model is depth  #
##########################
list.SimulationModelSettings[[9]] <- list(string.SimulationModel = "depth",
                            vector.AncestralState = runif(n = numeric.NumberTrainingReps, min = 0, max = 100),
                            matrix.STACK_lrate_nodes = matrix(replicate(n = numeric.NumberTrainingReps, samp
                            matrix.STACK_lrate_rates = matrix(replicate(n = numeric.NumberTrainingReps , runi
                            matrix.STACK_AncShiftNode = replicate(n = numeric.NumberTrainingReps, sample(siz
                            matrix.STACK_AncShiftValue = replicate(n = numeric.NumberTrainingReps , rnorm(n =
                                         list.Rmatrix = list.Rmatrix,
                            vector.depth = runif(n = numeric.NumberTrainingReps, min = 0, max = 10))

##########################
# tenth model is lrate  #
##########################
list.SimulationModelSettings[[10]] <- list(string.SimulationModel = "lrate",
                             vector.Sig2 = rexp(n = numeric.NumberTrainingReps, rate = 1),
                             vector.AncestralState = runif(n = numeric.NumberTrainingReps, min = 0, max = 100)
                             matrix.STACK_lrate_nodes = matrix(replicate(n = numeric.NumberTrainingReps, samp
                             matrix.STACK_lrate_rates = matrix(replicate(n = numeric.NumberTrainingReps , run
                             matrix.STACK_AncShiftNode = replicate(n = numeric.NumberTrainingReps, sample(siz
                             matrix.STACK_AncShiftValue = replicate(n = numeric.NumberTrainingReps , rnorm(n
                                          list.Rmatrix = list.Rmatrix,
                             matrix.lrate_node = matrix(replicate(n = numeric.NumberTrainingReps, runif(n = 1
                             matrix.lrate_rate = matrix(replicate(n = numeric.NumberTrainingReps , runif(n =

############################
# eleventh model is nrate  #
############################
list.SimulationModelSettings[[11]] <- list(string.SimulationModel = "nrate",
                             vector.Sig2 = rexp(n = numeric.NumberTrainingReps, rate = 1),
                             vector.AncestralState = runif(n = numeric.NumberTrainingReps, min = 0, max = 100)
                             matrix.STACK_lrate_nodes = matrix(replicate(n = numeric.NumberTrainingReps, samp
                             matrix.STACK_lrate_rates = matrix(replicate(n = numeric.NumberTrainingReps , run
                             matrix.STACK_AncShiftNode = replicate(n = numeric.NumberTrainingReps, sample(siz
                             matrix.STACK_AncShiftValue = replicate(n = numeric.NumberTrainingReps , rnorm(n
                                          list.Rmatrix = list.Rmatrix,
                             matrix.nrate_time = matrix(replicate(n = numeric.NumberTrainingReps, runif(n = 1
                             matrix.nrate_rate = matrix(replicate(n = numeric.NumberTrainingReps , runif(n =

####################
# SIMULATE TRAITS! #
####################
handle.RESULTS_TEST <- TraitTrain(handle.Phylogeny = handle.PrimatePhylogeny,
                            list.SimulationModelSettings = list.SimulationModelSettings,
                                       logical.PIC = T, logical.PROJECT = T)
```

Index

Simulate.TraitTrain.BM_00, 2
Simulate.TraitTrain.BM_STACKED_01, 3
Simulate.TraitTrain.BM_STACKED_10, 5
Simulate.TraitTrain.BM_STACKED_11, 7
Simulate.TraitTrain.delta_00, 9
Simulate.TraitTrain.delta_STACKED_01, 10
Simulate.TraitTrain.delta_STACKED_10, 12
Simulate.TraitTrain.delta_STACKED_11, 14
Simulate.TraitTrain.depth_00, 16
Simulate.TraitTrain.depth_STACKED_01, 17
Simulate.TraitTrain.depth_STACKED_10, 19
Simulate.TraitTrain.depth_STACKED_11, 20
Simulate.TraitTrain.EB_00, 22
Simulate.TraitTrain.EB_STACKED_01, 24
Simulate.TraitTrain.EB_STACKED_10, 26
Simulate.TraitTrain.EB_STACKED_11, 27
Simulate.TraitTrain.kappa_00, 29
Simulate.TraitTrain.kappa_STACKED_01, 31
Simulate.TraitTrain.kappa_STACKED_10, 32
Simulate.TraitTrain.kappa_STACKED_11, 34
Simulate.TraitTrain.lambda_00, 36
Simulate.TraitTrain.lambda_STACKED_01, 38
Simulate.TraitTrain.lambda_STACKED_10, 39
Simulate.TraitTrain.lambda_STACKED_11, 41
Simulate.TraitTrain.lrate_00, 43
Simulate.TraitTrain.lrate_STACKED_01, 45
Simulate.TraitTrain.lrate_STACKED_10, 47
Simulate.TraitTrain.lrate_STACKED_11, 49
Simulate.TraitTrain.nrate_00, 51
Simulate.TraitTrain.nrate_STACKED_01, 52
Simulate.TraitTrain.nrate_STACKED_10, 54
Simulate.TraitTrain.nrate_STACKED_11, 56
Simulate.TraitTrain.OU_00, 58
Simulate.TraitTrain.OU_STACKED_01, 60
Simulate.TraitTrain.OU_STACKED_10, 62
Simulate.TraitTrain.OU_STACKED_11, 63
Simulate.TraitTrain.trend_00, 65
Simulate.TraitTrain.trend_STACKED_01, 67
Simulate.TraitTrain.trend_STACKED_10, 68
Simulate.TraitTrain.trend_STACKED_11, 70
Simulate.TraitTrain.white_00, 72
Simulate.TraitTrain.white_STACKED_01, 74
Simulate.TraitTrain.white_STACKED_10, 75
Simulate.TraitTrain.white_STACKED_11, 77

TraitTrain, 78
